# French recommendations for the management of systemic necrotizing vasculitides (polyarteritis nodosa and ANCA-associated vasculitides)

**DOI:** 10.1186/s13023-020-01621-3

**Published:** 2020-12-29

**Authors:** Benjamin Terrier, Raphaël Darbon, Cécile-Audrey Durel, Eric Hachulla, Alexandre Karras, Hélène Maillard, Thomas Papo, Xavier Puechal, Grégory Pugnet, Thomas Quemeneur, Maxime Samson, Camille Taille, Loïc Guillevin, Collaborators, Vincent Audard, Olivier Aumaitre, Karine Briot, Patrice Cacoub, Pascal Cathebras, Dominique Chauveau, Olivier Chosidow, Laurent Chouchana, Vincent Cottin, Divi Cornec, Eric Daugas, Elisabeth Diot, Nicolas Dupin, Khalil El Karoui, Olivier Fain, Pierre Gobert, Philippe Guilpain, Mohamed Hamidou, Aurélie Hummel, Marie Jachiet, Stéphane Jouneau, Noémie Jourde Chiche, Cédric Landron, Claire Le Jeunne, Jean-Christophe Lega, Xavier Mariette, Nathalie Morel, Christian Pagnoux, Philippe Remy, Frédéric Vandergheynst

**Affiliations:** 1 Internal Medicine, CHU Cochin, AP-HP, Paris, France; 2 French Vasculitis Association, Paris, France; 3 Internal Medicine, CHU Lyon, Lyon, France; 4 Internal Medicine, CHU Lille, Lille, France; 5 Nephrology, HEGP, AP-HP, Paris, France; 6 Internal Medicine, CHU Bichat, AP-HP, Paris, France; 7 Internal Medicine, CHU Toulouse, Toulouse, France; 8 Internal Medicine, CH Valenciennes, Valenciennes, France; 9 Internal Medicine, CHU Dijon, Dijon, France; 10 Pulmonology, CHU Bichat, AP-HP, Paris, France; 11 Nephrology, CHU Henri Mondor, AP-HP, Paris, France; 12 Internal Medicine, CHU Clermont-Ferrand, Paris, France; 13 Rheumatology, CHU Cochin, AP-HP, Paris, France; 14 Internal Medicine, CHU Pitié Salpêtrière, AP-HP, Paris, France; 15 Internal Medicine, CHU Saint Etienne, Paris, France; 16 Nephrology, CHU Toulouse, Paris, France; 17 Dermatology, CHU Henri Mondor, AP-HP, Paris, France; 18 Pharmacology, CHU Cochin, AP-HP, Paris, France; 19 Pulmonology, CHU Lyon, Paris, France; 20 Rheumatology, CHU Brest, Paris, France; 21 Nephrology, CHU Bichat, AP-HP, Paris, France; 22 Internal Medicine, CHU Tours, Paris, France; 23 Dermatology, CHU Cochin, AP-HP, Paris, France; 24 Internal Medicine, CHU Saint Antoine, AP-HP, Paris, France; 25 Nephrology, Rhône Durance Clinic, Avignon, Paris, France; 26 Internal Medicine, CHU Montpellier, Paris, France; 27 Internal Medicine, CHU Nantes, Paris, France; 28 Nephrology, CHU Necker, AP-HP, Paris, France; 29 Dermatology, CHU Saint Louis, AP-HP, Paris, France; 30 Pulmonology, CHU Rennes, Paris, France; 31 Nephrology, CHU Marseille, Paris, France; 32 Internal Medicine, CHU Poitiers, Paris, France; 33 Internal Medicine, CHU Lyon, Paris, France; 34 Rheumatology, CHU Kremlin-Bicêtre, AP-HP, Paris, France; 35 General Medicine, CHU Cochin, AP-HP, Paris, France; 36 Mount Sinai Hospital, Toronto, Canada; 37 Internal Medicine, CHU Brussels, Bruxelles, Belgium

## Abstract

Systemic necrotizing vasculitis comprises a group of diseases resembling polyarteritis nodosa and anti-neutrophil cytoplasmic antibody-associated vasculitis (ANCA): granulomatosis with polyangiitis, eosinophilic granulomatosis with polyangiitis, and microscopic polyangiitis. The definitive diagnosis is made in cooperation with a reference center for autoimmune diseases and rare systemic diseases or a competency center. The management goals are: to obtain remission and, in the long term, healing; to reduce the risk of relapses; to limit and reduce the sequelae linked to the disease; to limit the side effects and the sequelae linked to the treatments; to improve or at least maintain the best possible quality of life; and to maintain socio-professional integration and/or allow a rapid return to school and/or professional activity. Information and therapeutic education of the patients and those around them are an integral part of the care. All health professionals and patients should be informed of the existence of patient associations. The treatment of vasculitis is based on variable combinations of glucocorticoids and immunosuppressants, chosen and adapted according to the disease concerned, the severity and/or extent of the disease, and the underlying factors (age, kidney function, etc.). Follow-up clinical and paraclinical examinations must be carried out regularly to clarify the progression of the disease, detect and manage treatment failures and possible relapses early on, and limit sequelae and complications (early then late) related to the disease or treatment. A distinction is made between the induction therapy, lasting approximately 3–6 months and aimed at putting the disease into remission, and the maintenance treatment, lasting 12–48 months, or even longer. The role of the increase or testing positive again for ANCA as a predictor of a relapse, which has long been controversial, now seems to have greater consensus: Anti-myeloperoxidase ANCAs are less often associated with a relapse of vasculitis than anti-PR3 ANCA.

**Supplementary Information:**

The online version contains supplementary material available at 10.1186/s13023-020-01621-3.

## Summary of the PNDS intended for the general practitioner

This summary was developed from the National Diagnostic and Care Protocol (PNDS)—systemic necrotizing vasculitis available on the site www.vascularites.org.

Systemic necrotizing vasculitis (SNV) comprises a heterogeneous group of diseases that are all characterized by inflammation of the arterial and/or capillary and/or venous blood vessels, leading to a change in the vascular wall as a whole. Stenosis or occlusion of the vascular lumen by thrombosis or intimal proliferation is the result of endothelial damage, which is the cause of clinical manifestations.

The diseases covered by this PNDS are systemic necrotizing vasculitis, namely:

- Polyarteritis nodosa (PAN).
- Anti-neutrophil cytoplasmic antibody-associated vasculitis (ANCA): granulomatosis with polyangiitis (GPA) (Wegener’s granulomatosis), eosinophilic granulomatosis with polyangiitis (EGPA) (Churg–Strauss syndrome), and microscopic polyangiitis (MPA).

Other types of vasculitis are either the subject of a specific PNDS or have no PNDS determined at the present time.

### Initial assessment

SNVs are a group of heterogeneous diseases, the management of which requires a multidisciplinary assessment adapted to each patient and coordinated by a hospital doctor. The assessment is made by:

- Reference centers and/or accredited autoimmune diseases and rare systemic diseases competence centers (Additional file 1).
- Specialists involved according to the clinical locations.
- General practitioners.

The objectives of the initial assessment report are to:

- Identify the initial clinical signs suggestive of a diagnosis of SNV.
- Confirm the diagnosis.
- Eliminate differential diagnoses.
- Specify the severity of the disease.
- Provide the information necessary for the choice of treatments to be prescribed.

The diagnosis should be put forward as a result of the combination of several clinical signs and/or biological anomalies, some non-specific (arthralgia or arthritis, myalgia, fever, etc.), others more suggestive (multiple mononeuropathy, necrotic purpura, kidney failure with microscopic hematuria, atrophic rhinitis, pulmonary nodules, persistent asthma, etc.) as well as biological signs (inflammatory syndrome, hypereosinophilia, and presence of ANCA).

The definitive diagnosis is based on the detection of histological lesions of necrotizing vasculitis on the biopsy of an affected tissue. In some cases, when a biopsy is not reasonable and/or easily feasible, or when the biopsies performed are normal and/or non-contributory, the diagnosis can be made on the basis of the combination of clinical signs as well as biological and immunological signs, such as the detection of ANCA with specific anti-PR3 or anti-MPO (MPO) and/or radiological anomalies such as the detection of arterial microaneurysms on the angiography.

The definitive diagnosis is made in cooperation with an autoimmune diseases and rare systemic diseases reference or competence center (available centers are listed on the DGOS Web site - http://www.sante.gouv.fr, or FAI2R website - http://www.fai2r.org/les-centres-fai2r).)

### Therapeutic management

The care of a patient with SNV must be multidisciplinary and coordinated by a hospital doctor in conjunction with an autoimmune diseases and rare systemic diseases reference center or a competence center and their contacts from different specialties, with the help of various paramedical professionals.

The objectives are:

- To obtain remission and, in the long term, healing.
- To reduce the risk of relapses.
- To limit and reduce the sequelae linked to the disease.
- To limit the side effects and the sequelae linked to the treatments.
- To improve or at least maintain the best possible quality of life.
- To maintain socio-professional integration and/or allow a rapid return to school and/or professional activity.

Information and therapeutic education of the patients and those around them are an integral part of the care.

All health professionals and patients should be informed of the existence of patient associations.

#### Treatment of systemic necrotizing vasculitis

We differentiate between:

The treatment of PAN, whether or not it is linked to an infection from the hepatitis B virus (HBV), the combination with HBV having become rare.

The treatment of ANCA-associated vasculitides (GPA, EGPA, and MPA).

The treatment of these types of vasculitis is based on variable combinations of glucocorticoids and immunosuppressants or immunomodulators chosen and adapted according to:

- Disease in question.
- Severity and/or extension of the disease.
- Underlying factors (age, renal function, etc.).

A distinction is made between the induction treatment, lasting around 3–6 months and aimed at putting the disease into remission (disease control), and the maintenance treatment, lasting 12–48 months, or even longer, which helps to consolidate remission and prevent the risk of a relapse. Relapses can occur several years after the remission and discontinuation of treatment. Prolonged monitoring of the patients is necessary.

Other treatments are combined according to the impairment observed and the progression. These could involve:

Preventive treatments for certain complications linked to the treatments (in particular, corticosteroid-induced osteoporosis, infections, and cardiovascular diseases).

Plasma exchanges in the case of rapidly progressive extracapillary glomerulonephritis or a severe intra-alveolar hemorrhage, although their exact role has yet to be determined following the negative results of the PEXIVAS study (international prospective study evaluating the role of plasma exchanges in ANCA-associated vasculitides).

Extrarenal purification sessions.

Surgical interventions (e.g., in the case of gastrointestinal perforations).

#### Pregnancy

A pregnancy in women with SNV must be managed in a gynecology-obstetrics department specializing in monitoring “at-risk” pregnancies and performed in cooperation with an autoimmune diseases and rare systemic diseases reference center or a competence center.

#### Children

Children with SNV must be managed together with or directly within an autoimmune diseases and rare systemic diseases reference center or a competence center with pediatric expertise, with the possible involvement of a reference or competence center for adults.

### Follow-up

Follow-up clinical and paraclinical examinations should be performed regularly in order to:

- Specify the course of the disease (remission or, conversely, worsening/progression).
- Detect and manage treatment failures and possible relapses early on.
- Limit and manage sequelae and complications (early and late) related to the disease or treatments early on.
- Limit the negative psychological consequences of the disease and its family, school, and/or socio-professional repercussions.

This follow-up is multidisciplinary and coordinated by a hospital doctor, in conjunction with the general practitioner, a reference and/or competence center, hospital contacts from different specialties, and with the help of various paramedical and medico-social professionals.

In the interval between visits to the specialists, the general practitioner plays an important role in the treatment of intercurrent pathologies in relation to SNV and its treatments, in close cooperation with the doctor at the autoimmune diseases and rare systemic diseases reference center or the competence center.

The possibility of a relapse must be raised before the reappearance of clinical signs and/or biological abnormalities present at the time of the diagnosis or the appearance of other signs suggesting new damage. During ANCA-associated vasculitides, an increase in the titer and/or recurrence of ANCA is not sufficient to diagnose a relapse, but it requires closer monitoring due to the increased risk of a relapse, especially in patients with kidney impairment. The role of the increase in or testing positive again for ANCAs as a predictor of a relapse, which has long been controversial, now seems to have greater consensus: Anti-MPO ANCAs are less often associated with a relapse of vasculitis than anti-PR3 ANCAs. The persistence of ANCA when undergoing a maintenance treatment, or 12 months from the start of a maintenance treatment, is associated with an increase in the relapse rate, according to the studies.

## Objective

The objective of this National Diagnostic and Care Protocol (PNDS) is to inform health professionals of the optimal management and care of a patient in whom a diagnosis of systemic necrotizing vasculitis has been made, namely:

- Polyarteritis nodosa (PAN).
- Anti-neutrophil cytoplasmic antibody-associated vasculitis (ANCA): granulomatosis with polyangiitis (GPA) (Wegener’s granulomatosis), eosinophilic granulomatosis with polyangiitis (EGPA) (Churg–Strauss syndrome), and microscopic polyangiitis (MPA).

The other primary types of vasculitis are or will be the subject of one or more PNDSs (giant cell arteritis, Takayasu’s arteritis, and Behcet’s disease), as secondary vasculitis which can complicate rheumatoid arthritis (LTC 22), Sjögren’s syndrome (LTC 31), or other systemic (e.g., LTC 21 or 31) or hematological (e.g., LTC 30, chronic myelomonocytic leukemia) diseases.

This PNDS is a practical tool to which the doctor can refer for the management of these diseases, especially when establishing the treatment protocol, together with the health insurance company medical adviser and the patient.

The PNDS cannot, however, consider all of the specific cases, all the comorbidities and all the therapeutic particularities or hospital care protocols. On the other hand, it establishes the essential procedures for the management of a patient suffering from systemic necrotizing vasculitis.

## Systemic necrotizing vasculitis

### Definition and classifications

Systemic necrotizing vasculitis (SNV) comprises a heterogeneous group of diseases that are all characterized by inflammation of the arterial and/or capillary and/or venous blood vessels, leading to a change in the vascular wall as a whole, endothelium, media, and adventitia, with fibrinoid necrosis. Stenosis or occlusion of the vascular lumen by thrombosis or intimal proliferation is the result of endothelial damage, which is the cause of clinical manifestations.

Most classifications are based on clinical and histological criteria. In 1990, the American College of Rheumatology (ACR) established the classification criteria for the main types of systemic vasculitis based on clinical, biological, and histological criteria, which are useful for standardization of patients in clinical studies, but they should not, however, be used as diagnostic criteria.

In 1994, the Chapel Hill Nomenclature proposed a definition of vasculitis putting into perspective the histological criteria and pathogenic mechanisms, especially taking into account the type and size of the vessels affected and the histological characteristics of the vascular impairment. This nomenclature was revised in 2012 and gave rise to a new text making it possible to add new vasculitides into the classification and to better specify the respective definitions of each of the vasculitides.

A distinction is thus made according to the predominance of the impairment:

- Vasculitides of large-caliber vessels (aorta and its dividing branches), including Takayasu’s arteritis and giant cell arteritis (formerly Horton’s disease).
- Vasculitides of medium-caliber vessels (main visceral arteries and their dividing branches), including PAN and Kawasaki disease.
- Vasculitides of small-caliber vessels (arterioles, capillaries, and venules), including ANCA-associated vasculitides, anti-glomerular basement membrane disease (formerly known as Goodpasture syndrome), and vasculitides by immune complex deposition. The lattermost include cryoglobulinemic vasculitis, IgAV (formerly known as Henoch–Schonlein purpura) vasculitides, anti-C1q vasculitis (formerly known as hypocomplementemic urticarial vasculitis syndrome or McDuffie syndrome).

### Epidemiology

SNVs are rare diseases. The annual incident rate varies between 1 and 65 cases/year/million inhabitants, depending on the vasculitis: GPA from 2 to 12 cases/year/million inhabitants and 24–218 cases/year/million inhabitants, respectively; EGPA from 0.9 to 4 cases/year/million inhabitants and 7.3–18 cases/year/million inhabitants, respectively; and MPA from 16 cases/year/million inhabitants and 25–184 cases/year/million inhabitants, respectively. The impact of PAN is not well known in France, but its prevalence was estimated at 31 cases/million inhabitants in 2002. Currently, the impact of PAN seems much lower and the HBV + cases have become rare.

They can affect subjects of all ages, with 40–60 years of age more at risk, regardless of gender. However, patients with MPA are on average 10 years older than patients with GPA, at the time of the diagnosis.

### Etiological factors

#### Genetic predisposition

A genetic association study has shown a genetic component in the pathogenesis of ANCA-associated vasculitis. Notably, these associations were more linked to the ANCA targets antigens than to the clinical phenotype (GPA or MPA).

The results of a genome-wide association study carried out at the European level during the EGPA showed that there were 11 variants associated with EGPA, two being specifically associated with negative ANCA forms and one (*HLA-DQ*) with anti-MPO ANCA.

Finally, mutations of the *ADA2* (or *CECR1*) gene coding for adenosine deaminase 2 have been demonstrated in children with a disease close to PAN, however, with an increased frequency of strokes.

#### Enabling factors

With PAN, the association between HBV infections and the onset of vasculitis was largely documented starting in the 1970s. The best prevention of HBV infections resulting from the generalization of vaccinations explains why PAN linked to HBV has today become very rare, and most often it is not linked to HBV. Authentic cases of PAN have been associated with other chronic viral infections, such as hepatitis C virus or HIV, as well as in hematological diseases, in particular myelodysplastic syndromes and chronic myelomonocytic leukemia.

In the case of ANCA-associated vasculitides, predisposing factors such as exposure to silica or dust have been discussed. Moreover, in a prospective, randomized study, chronic nasal carriage of *Staphylococcus aureus* has been demonstrated as being a factor leading to relapses, explaining the prescription of cotrimoxazole to prevent relapses.

With EGPA, the specific etiological agents remain unknown. Some environmental factors that preceded the clinical manifestations have, however, been reported: infectious agents, medications, allergy desensitization. The attributability of anti-asthmatic drugs has also been controversial for a long time, in particular leukotriene receptor antagonists such as montelukast or zafirlukast, and more recently, monoclonal anti-IgE antibodies, omalizumab. These drugs allow a reduction in the use of glucocorticoids. It is possible (but not proven) that their use could have revealed a latent EGPA by allowing the reduction of glucocorticoids rather than causing it directly.

#### Pathophysiology

##### Pathophysiology of PAN

The immunopathogenic mechanisms leading to the vascular lesions observed in PAN are probably heterogeneous. The most widely implicated mechanism, based on animal models, is the development of lesions induced by immune complexes. In many cases, PAN is the result of an HBV infection, and there is evidence to suggest that it is an immune complex deposition disease triggered by the HBs surface antigen in the case of an HBV infection. According to this hypothesis, the immune complexes activate the complement cascade, whose active products attract and in turn activate the neutrophils. These models do not, however, provide a clear explanation for the primary forms, not linked to the HBV.

##### Physiopathology of ANCA-associated vasculitides

The ANCA-associated vasculitides involve B and T lymphocytes, as well as innate immunity cells, in particular neutrophils and the monocytes/macrophages during GPA and MPA, and the eosinophils during EGPA, in a predisposed genetic context listed above.

##### Mechanisms behind ANCA

Hypotheses have been put forward to understand the origins of ANCA, in particular the existence of a triggering environmental or infectious factor due to the early impairment of the upper airways, especially in GPA. The current hypothesis is that natural ANCA would preexist in patients and would become pathogenic following various events: exposure to exogenous antigens, ectopic or abnormal expression of the ANCA target autoantigens, in a context promoting their immunogenicity (by expression of modified antigens or by apoptotic bodies by neutrophil extracellular traps), dysfunction of the regulatory cells controlling tolerance toward ANCA antigens.

##### Pathogenic role of ANCA

The pathogenic role of ANCA has been established by the convergent results of clinical and experimental studies; the data are, however, valid only for anti-MPO ANCA. In vitro*,* active neutrophils expressing PR3 or MPO on the surface can be activated by ANCA. In vivo*,* the role of the anti-MPO ANCA pathogen has been demonstrated by the passive transfer of ANCA or anti-MPO splenocytes in wild-type mice in the first case or mutated for the *RAG2* gene in the second case. This adoptive transfer was combined with the onset of extracapillary glomerulonephritis a few days later. In addition, a case of maternal–fetal transmission of anti-MPO ANCA with development of vasculitis in the newborn has been described.

The pathogenic role of anti-PR3 ANCA is, on the other hand, less clear. It has been suggested in a murine model of humanized mice, with the onset in nearly a third of cases of glomerulonephritis and/or pulmonary capillaritis after the adoptive transfer of anti-PR3 ANCA coming from patients.

##### Role of granulomas during granulomatosis with polyangiitis

Granulomas involve antigen-presenting cells, T lymphocytes, and also B lymphocytes. The pathophysiological sequence leading to the onset of the disease during GPA could take place according to the following scenario:

- PR3 expression in the upper airways, in response to an external stimulus, with activation of the dendritic cells.
- Migration of dendritic cells into the lymph nodes where they present the antigen to T lymphocytes which differentiate into Th1 lymphocytes under the effect of interleukin-12 (IL-12) produced by the activated dendritic cells.
- Migration of Th1 lymphocytes into the lungs, where they secrete tumor necrosis factor alpha (TNF-α) and interferon gamma (IFN-γ) controlling the migration and maturation of macrophages and the formation of granulomas and tissue destruction.
- Activation by T lymphocytes stimulated by self-reactive B lymphocytes producing anti-PR3 ANCA.

##### Role of proteinase 3 during granulomatosis with polyangiitis

PR3 expressed on the surface of neutrophils, the quantity and/or function of which can be modified according to genetic polymorphisms, can disrupt the resolution of an inflammatory response after macrophages have performed their phagocytic function. In fact, the expression of PR3 on the surface of apoptotic cells is at the source of increased production of pro-inflammatory cytokines. In addition, the micro-environment created by the membrane expression of PR3 is at the source of Th2 and Th9 polarization of the adaptive immune response. The addition of anti-PR3 ANCA also directs the immune response toward Th17-type polarization, with increased production of IL-17. These recent data have shown that PR3 plays a role in the sustainability of the inflammatory response during GPA, by preventing a return to homeostasis.

##### Special case of eosinophilic granulomatosis with polyangiitis

Eosinophils probably play a decisive role in the pathophysiology of the disease. They are more numerous and activated during the phases of disease activity, in part due to increased production of Th2-type cytokines (IL-4, IL-13, and IL-5) by stimulated T lymphocytes. In addition, there is a close relationship between IL-5 serum levels and disease activity. This production of Th2-type cytokines is also believed to be at the source of activation of B lymphocytes, which could in some cases lead to the onset of pathogenic ANCA, most often of anti-MPO specificity. This central role of IL-5 is at the source of the development of targeted therapies.

### Progression and prognosis

#### Polyarteritis nodosa

In its systemic form, PAN is an acute disease that can be serious and life-threatening if it is not treated properly.

The study conducted by the GFEV on 348 patients with PAN showed that after an average follow-up of 68.3 months: 21.8% of the patients relapsed (28% of those with PAN not linked to HBV compared to 10.6% of those with PAN linked to HBV), 24.7% of the patients died (19.6% of those with PAN not linked to HBV compared to 34.1% of those with PAN linked to HBV), and survival without a relapse at 5 years was 59.4% (95% confidence interval (CI) 52.6–67.0) compared to 67.0% (95% CI 58.5–76.8) in those with PAN not linked to HBV compared to those linked to HBV, respectively.

Although treatment is now able to achieve favorable results in the majority of patients, some relapse or die from complications related to the disease or the treatment.

Factors for a poor prognosis at the time of the initial diagnosis are grouped together in the Five-Factor Score (FFS), established in 1996 and revised in 2009 (Box 1).

**Box 1:** Five-Factor Score (FFS)

The FFS prognostic score in its 1996 version studied the factors impacting overall survival during PAN and EGPA, but not of other necrotizing vasculitides. However, the distinction between PAN and MPA was not straightforward at that time, and the two diseases were sometimes confused.	
The items found to have a negative impact on survival are proteinuria > 1 g/24 h, serum creatinine > 140 µmol/L, specific cardiomyopathy, severe gastrointestinal impairment, and central nervous system impairment. The score is zero when all of these factors are absent. The FFS is 1 when only one of the prognostic criteria is present; the FFS is 2 when two criteria are present.	
The FFS prognostic score was revised in 2009 to include other SNVs, namely GPA and MPA. The items having a negative impact on survival are age > 65 years, serum creatinine > 150 μmol/L, specific cardiomyopathy, severe gastrointestinal impairment, and the absence of any ENT impairment (only for GPA and EGPA).	

##### Definitions

Severe gastrointestinal impairment: severe gastrointestinal damage, i.e., intestinal perforation, hemorrhage, and pancreatitis. Appendicitis and cholecystitis are not included in the score when they represent the only manifestation of vasculitis or are found incidentally in a histological sample.

Specific cardiomyopathy: presence of clinical symptoms (e.g., pulmonary edema), excluding biological parameters, such as B-type natriuretic peptide (BNP), not determined for the majority of patients, in the absence of clinical symptoms. Echocardiography or other complementary tests were not taken into account for the diagnosis of heart failure.

Specific central neurological damage: damage to the entire central nervous system (stroke, pachymeningitis, pituitary impairment, etc.) and impairment to the cranial pairs, but does not, by definition, include peripheral neuropathies. Psychiatric disorders or mental confusion related to proven organic damage (meningitis, abnormalities on the brain imaging, etc.) are also included.

ENT impairment: presence of clinical symptoms confirmed by an otolaryngologist (ORL) examination and computed tomography. When ENT symptoms were not present, specialized investigations were not routinely carried out.

The clinical symptoms included in the score are those directly attributable to active vasculitis. For example, when heart failure is combined with preexisting high blood pressure or when a gastrointestinal hemorrhage is induced by a drug, the symptoms are not taken into account to determine the score.

In addition to the information provided by the FFS concerning overall survival up to 5 years, its interest also and above all lies in the adaptation of the treatments administered according to the severity of the disease.

The editorial and proofreading group positioned itself for the use of the 1996 FFS during PAN and EGPA.

During GPA and MPA, in a desire to synchronize the management with international practices, the use of the concept of “severe illness” and “non-severe illness” was preferred to the FFS to adapt the therapeutic strategy. Indeed, the FFS defines the parameters impacting overall survival and not the functional prognosis of certain disorders, and these latter can sometimes justify the prescription of an immunosuppressant treatment.

Thus, “severe” manifestations group together (list not restricted to these manifestations alone and are to be adapted to the clinician’s opinion): specific cardiomyopathy, mesenteric ischemia, severe intra-alveolar hemorrhage with respiratory distress, rapidly progressive kidney failure, specific stroke, impairment of the cranial pair(s), and multiple mononeuropathy with severe motor impairment.

#### Granulomatosis with polyangiitis

GPA is a serious type of vasculitis whose progression, left untreated, always leads to death. The vital prognosis has improved considerably, with a 10-year survival rate of 75% in the latest studies available. The progression of GPA is marked by very frequent relapses, half of the patients relapsing within 5 years after the first onset of the disease. The frequency of these relapses justifies a heavy and prolonged treatment, which explains the significance of iatrogenic complications in the prognosis of the disease. Infections and severe flare-ups are the leading cause of death in the first year of treatment, while cardiovascular complications are the leading cause of death in the long term. The factors related to poor prognosis at the time of initial diagnosis, correlated with excess mortality as a surrogate, were integrated into the version of the Five-Factor Score revised in 2009 (Box 1).

The sequelae are defined as irreversible lesions which are not due to the activity of vasculitis or to minimally persistent activity.

Patients frequently have ENT sequelae with persistent nasal crusts and epistaxis, while vasculitis is otherwise controlled. Deafness is a debilitating sequela of the disease. The progression to terminal kidney failure is the dreaded complication of severe or repeated kidney impairment.

Finally, there is talk of minimally persistent activity (*grumbling disease*) when a patient, otherwise with perfectly controlled vasculitis, shows minor persistent symptoms, such as arthralgia, fatigue, or atrophic rhinitis at a minimum. It is often difficult to differentiate between the sequelae. In practice, this low level of activity usually does not require increasing the treatment except for a possible slight increase in immunosuppression or corticosteroid therapy.

#### Microscopic polyangiitis

The course of MPA can be marked by relapses, with around a third of patients relapsing within 5 years of achieving complete remission. Although it is not yet possible to clearly identify the subgroup of patients at risk of relapse, several studies have shown the protective role of renal impairment with serum creatinine > 200 µmol/L. The overall mortality of patients with MPA was around 30% in 5 years. This higher mortality compared to GPA is partly explained by the highest average age of 10 years at the diagnosis of MPA. Most deaths occur in patients with the most severe forms, with one or more poor prognosis factors according to the 1996 Five-Factor Score or in its version revised in 2009 (Box 1). Survival at 10 years reaches 85% in the most recent studies, which included patients with severity criteria and treated with cyclophosphamide in induction.

#### Eosinophilic granulomatosis with polyangiitis

The progression and prognosis of EGPA are above all linked to the weight of the treatments, in particular corticosteroid therapy, and of the cardiac impairment when it is present.

Clinical remission is obtained in approximately 90% of the cases, but relapses occur in 60% of the cases during the decrease in corticosteroid therapy.

We must distinguish, on the one hand, the exacerbations of asthma and/or ENT impairment, which are the most frequent and occur throughout the course of vasculitis even after its prolonged remission and, on the other hand, relapses linked to an authentic flare-up of vasculitis, which most often occur in the first years of development.

These exacerbations of asthma and/or ENT impairment and relapses of vasculitis are most often minor, occurring during the decrease in corticosteroid therapy at doses lower than 10 mg/day, justifying the maintenance of corticosteroids over the long term or the introduction of immunosuppressant treatments. The overall 10-year survival is around 85%, with deaths linked primarily to heart damage or complications from the treatment. In fact, around 80% of patients are treated with long-term low-dose corticosteroids.

As with other SNVs, the factors related to a poor prognosis at the time of the initial diagnosis were analyzed in the initial version of the Five-Factor Score in 1996 and in the one revised in 2009 (Box 1).

### Treatments

The adaptation of specific treatments depends on the type and severity of the disease, the risk of relapse, the tolerance of previous treatments, and other factors (age, kidney function, etc.).

The combined use of corticosteroids and immunosuppressants and/or immunomodulators (initially cyclophosphamide and more recently rituximab) radically transformed the prognosis of these diseases, whose mortality rate without treatment in the years 1960–1970 was around 100% at 2 years.

## Method

The first version of this PNDS was developed according to the specifications drawn up by the French National Health Authority (HAS) (www.has-sante.fr). After a critical analysis of the international literature, the PNDS was discussed by a multidisciplinary group of experts. The proposals of this group were submitted to an editing group which reviewed each of the stated proposals. The corrected document was discussed and validated by the multidisciplinary group of experts. Furthermore, the therapeutic proposals were re-read by the French Agency for the Health Safety of Health Products) (AFSSAPS).

This new version is limited to updating the previous PNDS following numerous studies published since then and is under the sole responsibility of the signers and reviewers of this document. Once it has been finalized, HAS will record it on its Web site without endorsing its content, which is placed under the authority of its authors and the French Vasculitis Study Group (GFEV).

## Initial assessment

### Objectives

The initial objectives when management SNV are to:

- Know how to identify the first signs of systemic necrotizing vasculitis.
- Confirm the diagnosis of systemic necrotizing vasculitis.
- Rule out differential diagnoses.
- Specify the severity of the disease, type of organs impaired, and respective degrees of impairment.
- Provide indications to guide the choice of treatments (assessment of comorbidities likely to influence the prognosis or tolerance of treatments, etc.).

All of these elements are essential for providing patients with the information they need to manage them.

### Professionals involved

The initial management of a patient with SNV is multidisciplinary and coordinated by a hospital doctor. It is done by the identified network and contacts of the autoimmune diseases and rare systemic diseases reference centers and competence centers:

- Doctors of various specialties may be involved, in particular internists, clinical immunologists, rheumatologists, nephrologists, pulmonologists, nephro- or rheumato-pediatricians but also, depending on the clinical situation, neurologists, hematologists, gastroenterologists, ophthalmologists, ENT doctors, dermatologists, cardiologists, geriatricians, radiologists, etc.
- General practitioners.
- Medical and paramedical professions involved in making the initial assessment (biologists, pathologists, radiologists, nurses, physical therapists, dieticians, pain unit doctors, etc.).
- Resuscitators and emergency physicians.

Doctors in charge of patients may include them in national registers and/or current treatment protocols (https://www.vascularites.org/).

### Diagnostic approach and assessment of severity

#### Clinical and paraclinical examinations

Through a physical examination and paraclinical examinations, the doctor searches for objective elements necessary for the diagnosis.

##### Diagnostic tests

- The diagnosis is clinically suspected.
- Ideally, it is confirmed by the results of a biopsy of an impaired organ.
- A search for ANCA is essential, because its specificity in the context of vasculitis is around 100% (Box 2).
- If PAN is suspected, HBV serology possibly supplemented by a search for the DNA of the virus (and also of HCV and HIV) is necessary to identify a type linked to this infection, knowing that performing these serologies is essential in the pre-therapeutic assessment. A gastrointestinal and renal arteriography is desirable in case of abdominal pain and/or before a possible kidney puncture biopsy (Box 3).

**Box 2:** Screening for ANCA

Anti-neutrophil cytoplasmic antibodies (ANCA) form a family of autoantibodies directed against antigens contained in the primary granules (or azurophils) of the cytoplasm of neutrophils (but also of monocytes), above all myeloperoxidase (MPO) and proteinase 3 (PR3). The other ANCA targets (BPI, elastase, cathepsin G, lactoferrin) are of no clinical relevance and should therefore not be sought.	
ANCA research was based on the combination of two techniques: indirect immunofluorescence (IIF) on the one hand, and a technique studying the specificity of ANCA compared to MPO and PR3 on the other. The specific tests for the detection of anti-MPO and PR3 vary from center to center but are based above all on ELISA, flow fluorimetry (Luminex® instrument), or the dot blot*.* International consensus proposed in 2017 that tests targeted on the antigen, both more sensitive and specific than IIF, should be used as a *first-line* screening method for patients suspected of ANCA-associated vasculitides, e.g., *without going through a preliminary screening on IIF*	
However, specific immunoassays can be used by default, with false negatives, and can be supplemented by the IIF or another *second-line* validation test in the event of a strong clinical suspicion	
In severe forms, the rapid dot blot detection technique makes it possible to obtain an anti-PR3 and anti-MPO result in a few hours	
The detection of ANCA with anti-PR3 or anti-MPO specificity may, in the absence of a histological confirmation, be sufficient in a suggestive clinical context for retaining the diagnosis of vasculitis combined with ANCA and rapidly starting the treatment

**Box 3:** Renal biopsy during vasculitides

When diagnosing systemic vasculitis, a renal puncture biopsy with optical microscopy and immunofluorescence examination is:	
• Recommended if there is proteinuria compatible with a glomerular origin (made up of > 60% albumin) and microscopic hematuria	
• Recommended if there is a deterioration in kidney function (increase in serum creatinine and/or decrease in estimated glomerular filtration rate, eGFR), in the absence of an identifiable cause	
• Discussed in the case of isolated hematuria, after ruling out a pathology of the urinary tract, or in the case of isolated proteinuria, in the absence of another identifiable cause	
Conversely, it is contraindicated in cases of suspected PAN (where the impairment is most often vascular and not glomerular), due to often severe high blood pressure and the risk of bleeding related to the frequent existence of renal microaneurysms, which must be detected by imaging	
The diagnostic value of the renal needle biopsy is most important in the first month after starting the treatment	
In the immediate aftermath of vasculitis with histologically confirmed renal impairment, a control biopsy may be proposed in the event of worsening of the kidney function under treatment not explained by the initial histology (sampling problem or refractory form)	
A while after the initial flare-up, it will be indicated in the event of renal signs which can suggest a relapse of renal vasculitis: reappearance of microscopic hematuria, rapid deterioration of kidney function (serum creatinine, eGFR) with an increase in proteinuria, in the absence of another identifiable cause and other documentation of the relapse	
On the other hand, the mere persistence of low abundance microscopic hematuria or proteinuria following a flare-up of renal vasculitis does not justify a repetition of the kidney biopsy	
The kidney biopsy confirms the diagnosis of vasculitis combined with ANCA, by showing necrotizing glomerulonephritis without deposits of immunoglobulins or a supplement (called pauci-immune extracapillary glomerulonephritis), and sometimes impairment to intrarenal arterioles, or even rarely granulomatous lesions in GPA. It makes it possible to rule out differential diagnoses in cases of glomerulopathy with the presence of ANCA (infectious endocarditis, vasculitis with anti-glomerular basement membrane antibodies, lupus glomerulonephritis, glomerulonephritis combined with inflammatory bowel disease, etc.) or to show the presence of renal lesions secondary to another pathology (diabetes, high blood pressure), unrelated to vasculitis. It is essential in the rare cases of systemic necrotizing vasculitis with a negative immunological assessment and, in particular, negativity of ANCA or in case of the presence of both ANCA and anti-glomerular basement membrane antibodies (anti-MBG)	
In addition, the kidney biopsy makes it possible to specify the renal prognosis, in particular, due to the classifications proposed by Berden et al. (distinguishing focal, crescent, mixed, or sclerotic forms) and Brix et al. (quantifying interstitial fibrosis and tubular atrophy and the percentage of normal glomeruli)	

Finally, it should be noted that certain infectious diseases, in particular infectious endocarditis or tuberculosis, as well as chronic inflammatory bowel diseases, may be combined with the presence of ANCA, often “atypical” but sometimes with a specificity that is essentially directed against PR3.

Also, the consumption of cocaine cut with levamisole can induce levamisole vasculitides, during which the positivity of ANCA is frequent, in particular with a double specificity of anti-PR3 and anti-MPO.

##### Test for lesion assessment and pre-therapeutic assessment

Certain tests are necessary, but waiting for their results should not delay the start of the treatment.

- A complete blood count, platelets, prothrombin time (PT)/activated partial thromboplastin time (APTT), C-reactive protein (CRP), and fibrinogen to search for an inflammatory syndrome or hypereosinophilia, and a pre-therapeutic assessment.
- Kidney assessment: blood and urine electrolytes, serum creatinine, eGFR (Modification of Diet in Renal Disease (MDRD) or Chronic Kidney Disease Epidemiology Collaboration (CKD-EPI) formula), urine strip, protein/creatinine ratio of urea sample (possibly supplemented by urine protein electrophoresis), search for microscopic hematuria, kidney biopsy.
- Lung assessment: chest x-ray (front); chest scan (without injection in case of kidney failure); respiratory functional examinations in the case of interstitial radiological syndrome (flow-volume loop, plethysmography, and measurement of diffusing capacity of the lungs for carbon monoxide, DLCO), and possibly supplemented by performing a 6-min walk test (search for desaturation during exercise). In the event of a suspected alveolar hemorrhage and/or pulmonary impairment on the imaging, a bronchial fibroscopy and bronchioloalveolar lavage (BAL) can be performed with bronchial biopsies (in case of a nodule or lung mass or endobronchial lesion) and microbiological samples (differential diagnosis). A BAL can macroscopically lead to suspecting an alveolar hemorrhage (red or pink liquid), but it must be necessarily confirmed (and quantified) by ascertaining the Golde score (positive if > 100).
- ENT assessment: specialized test and sinus scan for patients with or suspected of having GPA or EGPA.
- Cardiac assessment: clinical test for functional and physical signs of cardiac impairment, systematic electrocardiogram, and transthoracic cardiac ultrasound; examinations of the coronary arteries in case of a doubt about a coronary impairment. In case of EGPA and/or suspected cardiac impairment, other examinations are useful (troponin, BNP or NT-pro-BNP, even a cardiac MRI). The role of a cardiac MRI has first place during the EGPA. The interest of cardiac MRI could lie mainly in the prognostic assessment of cardiac impairment, by evaluating the progression of lesions after induction therapy compared to the initial lesions. The use of a cardiac MRI for the purpose of detecting asymptomatic cardiac impairment on first-line examinations could lead to excessive treatment of the patients concerned and a poor prognostic impact on survival for patients with clinically significant cardiomyopathy.
- Neurological evaluation: electromyogram to be performed in the event of clinical abnormalities suggestive of peripheral neuropathy; cerebral and/or spinal MRI or even lumbar puncture in the event of clinical abnormalities leading to central neurological impairment.
- Hepatic evaluation, in particular in the event of PAN combined with HBV (transaminases [AST and ALT], GGT, alkaline phosphatases, total bilirubin), glycemia, phosphocalcic balance, electrophoresis of plasma proteins, CPK, LDH, lipid balance (pre-therapeutic assessment).
- Hematological assessment: etiological assessment of hypereosinophilia in case of suspected EGPA, and search for a myelodysplastic syndrome mainly in case of suspected PAN.
- Bone assessment with bone densitometry as soon as corticosteroid therapy is planned at a dose of 7.5 mg/day or more for more than 3 months.
- Other tests may be indicated depending on the clinical situation (e.g., ophthalmological examination, gastrointestinal endoscopies in the event of a presenting symptom, etc.), or within the framework of research protocols (positron emission tomography (PET) scan).
- There is no validated indication for PET during SNV. This test should not be carried out routinely for the assessment of the spread of the disease, nor for its monitoring. On the other hand, it cannot be ruled out that in certain specific situations, PET will find a place in the future to distinguish active lesions from fibrous sequelae (cardiac damage during EGPA, orbital mass during GPA, etc.).

#### Confirmation of diagnosis

The first symptoms of vasculitides are often non-specific, and it is their combination that leads to suspecting the diagnosis (general signs, arthralgia, myalgia, weight loss, fever, then multiple mononeuropathy, purpura, microscopic hematuria, etc.).

The diagnostic confirmation is based primarily on the biopsy of an impaired organ or tissue, which remains the gold standard. Performing a biopsy should not, however, delay treatment in the event of a strong diagnostic suspicion, and it must take into account the ratio of ease of procedure/usefulness so as not to expose the patient to a significant risk.

However, in certain cases, a clinical context very suggestive of the diagnosis, combined with biological and/or radiological abnormalities, can be considered sufficient to retain a diagnosis of vasculitis in the absence of histological evidence, for example, the presence of ANCA anti-PR3 specificity in most GPA (60% of localized forms, 85% of systemic forms); anti-MPO ANCA in 60% of MPA and in a third of the EGPA; or renal, hepatic, or gastrointestinal microaneurysms on the arteriography during the PAN.

Finally, certain differential diagnoses should also be sought during the stages of the initial assessment and/or if the course of treatment is not quickly satisfactory.

#### Differential diagnosis of primary systemic necrotizing vasculitis

The main differential diagnoses of primary SNV are:

- Malignant cancers and hemopathies (in particular lymphomas and myelodysplastic syndromes).
- Hypereosinophilic, myeloid, or lymphoid syndromes (for EGPA).
- Systemic infections (including infectious endocarditis, Q fever, tuberculosis). Infectious endocarditis is an important differential diagnosis to be mentioned, like tuberculosis, because it can be combined with ANCA without any specificity or even anti-PR3 specificity.
- Vasculitis complicating another autoimmune disease (lupus, rheumatoid arthritis, Sjögren’s syndrome, systemic scleroderma, etc.).
- Toxic (cocaine cut with levamisole) or medication-induced vasculitis (Additional file 2). Levamisole-induced vasculitides are frequently combined with the presence of ANCA, often with dual anti-MPO and anti-PR3 specificity.
- Other diseases that can mimic vasculitis: anti-phospholipid syndrome, cholesterol crystal embolism syndrome, atrial myxoma, calciphylaxis, etc.
- Much more rarely, vasculitis combined with ANCA and a disease with anti-MBG antibodies can be observed, justifying the search for anti-MBG antibodies in the event of pulmonary and/or renal impairment.
- ADA2 deficiency in early childhood forms of PAN often with fever, livedo, stroke, and gangrene.

The following paraclinical tests may therefore be useful in eliminating certain differential diagnoses:

- Serologies: HBV serology (HBsAg; anti-HBs and anti-HBc antibodies—for the diagnosis of PAN linked to HBV and for the pre-therapeutic assessment), HIV serology (for the diagnosis of secondary forms and for the pre-therapeutic assessment), HCV serology (viral RNA in the case of a positive or dubious serology—for the diagnosis of secondary forms and for the pre-therapeutic assessment).
- Blood cultures in case of fever, even systematic, cardiac ultrasound in case of an unknown murmur, in order to eliminate subacute bacterial endocarditis.
- Complementary immunological assessment: anti-nuclear antibodies (if present: search for soluble nuclear anti-antigen antibodies and native anti-DNA); rheumatoid factor (if present: search for anti-CCP antibodies and cryoglobulin); cryoglobulin search; anti-glomerular basement membrane antibodies (in case of a pulmonary renal syndrome or rapidly progressive glomerulonephritis); assay of the complement (CH50, C3, and C4 fractions); lupus-type circulating anticoagulant, anti-cardiolipin, anti-beta2-GP1 (in the event of signs suggesting an anti-phospholipid syndrome, thrombosis, distal ischemia, etc.).
- Depending on the context, other viral serologies can be carried out, as well as other examinations in search of bacterial or fungal infections (e.g., *Coxiella burnetii*, rickettsial serologies in the south of France, if in rural housing, Q fever, PCR *Tropheryma whipplei*).
- The search for cocaine and/or levamisole can be done in the hair to rule out these particular forms of vasculitis, in particular in cases of ANCA with dual anti-MPO and anti-PR3 specificity and in case of skin necrosis, in particular in the extremities.

Other tests may be indicated depending on the clinical situation and the differential diagnoses mentioned (e.g., bone marrow biopsy in the event of suspected lymphoma, serum immunofixation, search for cryoglobulinemia, supplemental assay, search for the JAK2 mutation or the *FIP1L1–PDGFRA* fusion transcript in the event of a suspicion of a myeloid hypereosinophilic syndrome, T lymphocyte immunophenotyping, and search for T clonality in the event of a suspicion of a lymphoid hypereosinophilic syndrome).

#### Assessment of severity of the disease

Each of the patient’s impairments (extension assessment), the form and severity of the vasculitis must be characterized. For all of the SNV covered by this PNDS, namely PAN, MPA, GPA, and EGPA, there is a prognostic score proposed by the GFEV, the Five-Factor Score (FFS). The initial FFS published in 1996 concerned PAN and EGPA exclusively. The FFS revised in 2009 and published in 2011 includes all the SNV, with the addition of GPA in particular. This score distinguishes the types of good (FFS = 0) or bad (FFS ≥ 1) prognosis with the sole criterion of measuring mortality (Box 1).

Intra-alveolar hemorrhages can be serious and responsible for a life-threatening respiratory distress syndrome and sometimes justifying mechanical ventilation. However, they are not statistically associated with excess mortality in prognostic studies, explaining their absence within the FFS. In addition, they are frequently combined with rapidly progressive glomerulonephritis, and their prognostic impact is then “erased” by the predominance of the renal impairment.

An understanding of all of the patient’s characteristics (age, history, background, kidney function, patient compliance, etc.) is also an essential element for guiding the therapeutic choices.

## Therapeutic management

### Objectives

In order to obtain a rapid response and improve the vital and functional prognosis, treatment of the SNV must be started early.

The main objective is to choose the best validated treatment and adapt it to each patient in order to:

- Obtain remission and sometimes healing.
- Reduce the risk of a relapse (in the range of 15–30% at 5 years for PAN, and 50% at 5 years for GPA).
- Limit and reduce the sequelae linked to the disease.
- Limit the side effects and the sequelae linked to the treatments.
- Improve the quality of life parameters affected by the disease.
- Maintain socio-professional and/or school integration and/or allow rapid return to social and/or school and/or professional activities.

### Professionals involved

The therapeutic management is multidisciplinary and coordinated by a hospital doctor, in conjunction with the general practitioner, a reference and/or competence center (Additional file 1).

It is carried out by the same professionals as those involved in the initial assessment, to which are added other paramedical professions (dieticians, occupational therapists, psychologists, child psychologists, child psychiatrists, etc.) and social assistance (social workers, care-givers, etc.).

### Therapeutic patient education

Therapeutic patient education (TPE) is care that cannot be separated from the management of a chronic disease. TPE is a key element in the overall management of the patient. This approach, which must be multidisciplinary, has been defined by the WHO:

“TPE is designed to help patients acquire or maintain the skills they need to best manage their lives with a chronic disease.

It is an integral and permanent part of the patient’s care; it includes organized activities, including psychosocial support, designed to make patients aware and informed about their illness, care, hospital organization and procedures, and behaviors related to health and the disease. The purpose of it is to help them (and their families) understand their illness and their treatment, work together and take on their responsibilities for their own care in order to help them maintain and improve their quality of life.

Oral or written information and advice on prevention may be provided by a health professional on a variety of occasions, but they are not the same as therapeutic patient education.”

“The educational approach is participatory and centered on the person and not simply transmitting knowledge or skills.”

“This is a partnership relationship between the patient, their social circle and the health care team whose purpose is to help the patient to take care of him/herself.”

Thus, TPE gives patients the opportunity to register within an individualized and controlled health path between a therapeutic standard proposed by the healthcare team and that of the patients resulting from their ideas and projects.

#### Purposes of TPE

TPE contributes to improving the patient’s health and improving their quality of life and that of their family and friends. Its purpose is to make the patients the main actor in their health and care journey by the appropriation of knowledge and skills that change their behavior.

The specific purposes of TPE are:

- Acquisition and maintenance of care skills by the patient.
- Relieve symptoms.
- Take into account the results of self-monitoring, self-measurement.
- Adapt to doses of medications.
- Perform technical procedures and care.
- Implement lifestyle modifications (balanced diet, physical activity).
- Prevent avoidable complications.
- Cope with the problems caused by the disease.
- Involve those close to the patient in the management of the disease, the treatments, and the resulting repercussions.
- Mobilization or acquisition of adaptation skills.
- Know oneself, have confidence in oneself.
- Know how to manage emotions and manage stress.
- Develop creative reasoning and critical thinking.
- Develop communication and interpersonal relations skills.
- Make decisions and solve problems.
- Set goals to be achieved and make choices.
- Observe oneself, assess oneself, and strengthen oneself.

#### Information and education do not have the same objectives

The first is to provide information to a “passive” patient. It is one of the duties of any doctor and it is a patient’s right (Law of March 4, 2002).

- The information must cover:
- SNV: natural history, treatments, and prognosis of the disease, in particular.
- Prescribed treatments and their possible side effects.
- Planning of routine or screening tests for possible complications and their results.
- Signs of a recurrence of vasculitis.
- Possibilities of participating in ongoing clinical studies.

The “educational” dimension goes further, because receiving information about the disease does not mean learning to live with it. TPE is based on an “active” attitude of a patient who questions, reacts, expresses him/herself, and has exchanges with a healthcare professional and/or with peers. Its purpose is the appropriation of knowledge and therefore its transformation by the person to whom it is transmitted into skills implemented in everyday life. Each person is unique, each situation unique. This personalized and benevolent “support” helps patients make decisions about the care, sometimes weighty and complicated, so as to improve their quality of life and, a fortiori, that of their family and friends. It also helps them with choices concerning their life project, their orientation, their administrative files, etc.

- The therapeutic education will focus in particular on the following points:
- Knowledge of the disease, symptoms, and warning signs which should lead to a consultation with the general practitioner or a specialist (any modification or worsening of the symptomatology should be cause for a consultation).
- Precautions for women of childbearing age (risk of infertility or early menopause, need for contraception with certain immunosuppressant treatments, risks and possible contraindication to become pregnant and breastfeed) and for men (risk of sterility, teratogenic risks of the treatments).
- Vaccination recommendations (prevention of bacterial and viral infections).
- Education for corticosteroid treatment (compliance, precautions, healthy lifestyle, diet).
- Anticipation of problems with treatment compliance.
- Promote coordination with the attending physician and the other physicians and paramedics involved.
- Various means are made available to health professionals to help their therapeutic education projects. The reference and competence centers have, in particular, information missions, as well as the French Vasculitis Study Group through its Web site (https://www.vascularites.org/education-therapeutique/). Patient associations and Web sites can provide useful information (see List of useful links for health professionals and patients in Additional file 5).

#### TPE has four stages

The HAS (French National Health Authority) has issued recommendation guides to help the implementation of educational programs or procedures:

1. Development of an individualized educational diagnosis (shared educational interview) with the patient which allows him to define his needs, expectations, fears, beliefs, projects, etc.
2. Definition of a personalized TPE program which defines the self-care and adaptation skills that the patient can acquire and/or put to use.
3. Personalized planning and implementation of TPE sessions using highly codified content and learning methods.
4. Assessment of achievements at the end of the educational program (individual assessment of “skills”).

#### TPE in practice: three operational methods

We differentiate:

1. TPE programs with a medical approach; in accordance with national specifications, the French National Health Authority (HAS) has classified the methods of preparation and content of which are defined by a decree from the Minister of Health. These programs are implemented at the local level, after authorization by the regional health agencies (ARS). They take into account the daily life of the patient and the social, psychological, and environmental factors. They are built on scientifically based information (professional recommendations, relevant scientific literature, and professional consensus) and are augmented by feedback from patients, their relatives, and patient associations, in terms of content and educational resources. They are organized by a multidisciplinary healthcare team trained in TPE and peers (intervening patients, TPE experts, and members of patient associations).
2. Learning programs, aimed at the appropriation by patients of technical procedures allowing the use of a drug.
3. Support actions which aim to provide assistance and support to the patients, or those around them, in the management of the chronic disease (Table 1).

**Table 1:** TPE for patients with systemic vasculitis

Issues to discuss	Educational objectives (non-exhaustive list)
What is systemic vasculitis?	Be able to describe its clinical manifestations Understand the meaning of biological follow-up, know how to draw from its routine biological assessment the information necessary for following up on it Be able to explain in words the mechanism of the disease (chronic disease, autoimmunity, inflammation of the vessel wall, type of vessel impaired, etc.) Recognize the appearance of signs of clinical and biological activity of the disease and take appropriate action Introduction to the importance of regular monitoring
Treatments	Understand my treatment, be able to define the action and the side effects of my treatments, understand the side effects Know how to use my treatment on a daily basis, understand the need to take my treatment regularly Adaptation of the hygiene and diet rules when under corticosteroid therapy and/or an immunosuppressant treatment
Relapse of the disease	Know how to recognize a relapse of their illness Recognize the clinical and biological signs of a relapse Adapt their conduct and know how to call on the right resource/person Identify triggers and learn how to prevent them (especially the importance of good compliance with the treatment)
Hygiene and diet measures	Know and adapt their diet when under corticosteroid therapy (no excess salt or sugar), adopt a balanced diet Raise awareness of the risk of infection (reminder about vaccination, hygiene rules to avoid infectious contagion)
Living with it	Express their ideas and feelings about the disease Normalize the experience of fatigue and better use their energy Adopt measures centered on one’s well-being Develop personal adaptation strategies Develop the self-esteem damaged by illness (self-love, self-image, self-confidence) Discover and mobilize resources that can be used to combat the difficulties encountered on a daily basis (psychologist, social workers, investigation into an MDPH file, etc.) Express the impact of the disease on daily life and implement adaptation strategies

#### TPE for patients with systemic vasculitis

Patients who have already completed the TPE sessions can participate in reinforcement sessions.

#### One TPE for “family caregivers or close caregivers” who support patients with vasculitis

The role of caregivers is essential for supporting patients suffering from vasculitis. It is necessary to prevent, identify, direct, and take care of the needs and difficulties associated with this support. An individual and/or collective TPE dedicated to family caregivers can be offered to keep them from becoming exhausted. The TPE sessions for patients suffering from systemic vasculitis can be open to caregivers, who will be able to accompany the patient and participate with them in the TPE sessions.

### Role of patient associations

All health professionals and patients should be informed of the existence of patient associations by their doctor, reference and/or competence centers, institutional Web sites, and Orphanet (see List of useful links for health professionals and patients in Additional file 5).

These associations contribute to better overall management of the disease by promoting cooperation between patients, patient associations, caregivers, and medical, social, and administrative institutions.

The French Vasculitis Association, created in 2006, is a recognized not-for-profit association of general interest according to the law of 1901. It is made up of patients with vasculitis, their family and friends, and supporting members. It makes it possible to make connections between patients in order to break the isolation and to share experiences and information. It can contribute to improving the patient’s healthcare journey by relying on recognized care networks. The association distributes doctors’ information validated by its scientific council. It helps promote medical research and organizes charity events to support research.

Several actions are carried out to help patients live with the disease on a daily basis:

- Hotline 09 87 67 02 38 and email: association.vascularites@gmail.com.
- Organization of information meetings for patients with the assistance of specialists.
- Organization of meetings between patients.
- Laboratory seminars to understand the biological mechanism of vasculitis, understand the vocabulary used by doctors, know how to interpret the results of blood tests.
- SANOÏA tool, personalized follow-up sheet: https://www.sanoia-fiche-sante.com/vascularites-wegener (allows the patient to personalize his follow-up, alone or with the help of his doctor, to measure his health parameters with recognized scores, prepare for his next consultation by printing or downloading a follow-up report, etc.).
- Promoting TPE in France.
- Serious online patient education game in addition to TPE in person: https://vasco.online-virgo.com/. VASCO is a course allowing patients to view content on treatments, mechanisms, symptoms, vaccines, or other recommendations and prevention of osteoporosis, etc. Patients can self-assess through quizzes and interact with other users.
- Patient community for ComPaRe research: https://compare.aphp.fr. ComPaRe brings together patients with chronic diseases to advance research on these diseases, by answering questionnaires via the Internet.
- Production of videos and DVDs to review conferences of specialists.
- Production of animated films on the mechanisms of vasculitis (*Phil the Neutrophil*).
- Publication of leaflets/brochures providing information and recommendations on vasculitis accessible to patients.
- Institutional and administrative resources.

The contact details of the Association must be reported to the patient as soon as their inpatient care is set up:

French Vasculitis Association, 7 Rue de l’église 21,540 Blaisy-Bas, Phone: 09 87 67 02 38.

Email: association.vascularites@gmail.com.

Web site: http://www.association-vascularites.org

### Pharmacological treatments

For reasons of simplicity, the guides intended for doctors cite the therapeutic classes without detailing all the drugs indicated in the pathology concerned, nor all of their characteristics (see Summary of Product Characteristics, SPC).

However, each drug is only concerned within the precise framework of its marketing authorization (MA). In rare diseases, such as vasculitis, an off-label prescription may be written if it is based on recommendations from learned societies or groups of experts on the disease. However, these prescriptions must be evaluated in the context of prospective or retrospective cohorts. However, for any prescription of an off-label product or one without a recommendation, it is written under the sole responsibility of the prescriber and in a more appropriate manner after a multidisciplinary consultation meeting (RCP). The patient should be informed of the therapeutic decisions.

Treatment of SNV should start early. A prescription must often be written urgently, without waiting for the results of all the additional examinations that are not essential to the diagnosis and subsequent therapeutic choices, after a discussion of the indication with the reference and/or competence centers.

#### Drug treatment of systemic necrotizing vasculitis

A distinction is made between the treatment of PAN (linked or not to HBV) and that of ANCA-associated vasculitides. The treatment of exceptional cases of PAN linked to other viral infections requires rapid and specific management in a competence center and/or reference center.

The treatment of immediately life-threatening, uncontrolled, and/or refractory forms and/or of patients intolerant of a conventional treatment must be discussed with the doctors of a reference or competence center.

##### Treatment of systemic PAN

PAN not related to a viral infection

- Corticosteroid therapy.
- The initial treatment always includes corticosteroid therapy started at a dose of 1 mg/kg/day of a prednisone equivalent, with a maximum dose of 60 mg/day, except in specific cases.
- An intravenous (IV) bolus of methylprednisolone, usually at a dose of 7.5–15 mg/kg/day (not to exceed 1 g/bolus), depending on the severity and cardiovascular condition of the patient, may be administered for 1–3 consecutive days (before following up with oral corticosteroid therapy at a dose of 1 mg/kg/day of a prednisone equivalent). Methylprednisolone boluses should be reserved for clinical situations requiring a rapid therapeutic response. They are not useful in “cold” forms of the disease or when the clinical situation is progressive or not life-threatening or functionally threatening.
- After an initial treatment of 3 weeks at a dose of 1 mg/kg/day of a prednisone equivalent, the corticosteroids should be reduced. There is no internationally validated pattern of reduction. The total duration of corticosteroid therapy varies from 6 months (North American protocols) to 18–24 months (European protocols). It is proposed in France to follow, in the absence of a study available with sufficient follow-up, a regimen of reduction whose essential benchmarks are approximately 20 mg/day at 3 months, 10 mg/day at 6 months, and 5 mg/day at 1 year of a prednisone equivalent.
- Immunosuppressants.
- The therapeutic strategy during PAN is oriented depending on whether or not there are poor prognosis factors defined in the 1996 FFS (Box 1), the systemic forms with an FFS = 0 justifying corticosteroids alone, and those with an FFS ≥ 1 justifying a combination of corticosteroids and immunosuppressants.
- Non-severe forms without poor prognosis factor (FFS = 0)

Immunosuppressant therapy is not warranted as a first-line treatment in these forms. The immunosuppressant is only prescribed to patients whose PAN is not controlled by corticosteroids alone (failure to achieve remission or relapse of vasculitis), if it is necessary to spare the use of corticosteroids in the event of corticosteroid dependence on more than 7.5–10 mg/day of a prednisone equivalent (in order to reduce the risk of occurrence of side effects), or in case of intolerance to corticosteroids.

Recently, a prospective, randomized, placebo-controlled study evaluated the effectiveness of the systematic addition of azathioprine to first-line corticosteroid therapy in vasculitis with FFS = 0, with the aim of preventing a relapse, limiting sequelae, but also sparing the use of corticosteroids (CHUSPAN2 trial). This study did not demonstrate superiority of azathioprine in these indications compared to corticosteroid therapy alone. However, in the case of related comorbidities which could be aggravated by corticosteroid therapy, the addition of immunosuppressant therapy to corticosteroid therapy can be discussed on a case-by-case basis.

- In situations where immunosuppressant therapy is indicated as a second line:
- In the absence of poor prognosis factor (FFS = 0), the choice of an immunosuppressant will preferably be focused on azathioprine (orally at a dose of 2–3 mg/kg/day) (Box 4) or methotrexate (orally or subcutaneously at a dose of 0.3 mg/kg/week) (Box 5), for a period of 12–18 months, compared with the treatment of ANCA-associated vasculitides. The prescription of mycophenolate mofetil in PAN has not been evaluated and requires, on a case-by-case basis, the opinion of the reference center and/or a competence center.
- If poor prognosis factors appear (FFS ≥ 1), the choice of an immunosuppressant will preferably be cyclophosphamide, according to the same methods as the treatment of forms with a poor prognosis factor(s) at the initial diagnosis (described below).
- Forms with poor prognosis factor(s) (FFS ≥ 1).

**Box 4:** Methods of administering azathioprine

Azathioprine is administered orally at a dose of 2 mg/kg/day in one, two, or three doses daily, without exceeding 200 mg/day (based on published therapeutic trials) and rounded up to the multiple dose of 25 mg higher (e.g., for a 70 kg patient, the dose will be 150 mg/day). This dose may be increased to 3 mg/kg/day by the doctor if he deems it useful (in the event of a partial response to 2 mg/kg/day), in the absence of studies having proven better efficacy of the drug azathioprine at a dose of 3 mg/kg/day, however. The maximum dose should not exceed 200 mg/day, regardless of the patient’s weight. Conversely, the doctor may reduce the daily dose by 25–50 mg in the event of a minor side effect in order to improve the digestive or hematological tolerance of the treatment. If this is not enough and/or if the side effect observed is serious from the start, the treatment must be definitively stopped When deciding to introduce azathioprine, the doctor can now rely on recommendations from the National Pharmacogenetic Network (RNGx) published in 2017. A warning regarding the genetic deficit in TPMT (thiopurine methyltransferase) and the risk of rapid development of myelosuppression is present in the SPC for azathioprine. There are, however, no pharmacogenetic recommendations in the SPC, unlike the American SPC The Clinical Pharmacogenetics Implementation Consortium and the RNGx recommend the search for a TPMT deficiency based on the identification of the allelic variants TPMT*2, TPMT*3A, TPMT*3B, TPMT*3C or on the phenotyping of TPMT allowing classification of individuals based on their metabolic capacity and suggest dose adjustments based on the TPMT status However, there is no study showing that an adjustment of the doses based on the genotypic study made it possible to reduce the risk of hematological events, in particular during chronic inflammatory diseases of the intestine. Thus, carrying out this test does not rule out strict hematological monitoring, especially in the first weeks of treatment The concomitant prescription of a urate-lowering treatment with allopurinol or febuxostat is contraindicated (increase in spinal toxicity). If allopurinol or febuxostat cannot be interrupted, the choice should be made for another immunosuppressant Azathioprine is usually prescribed for 12–24 months (optimal duration not defined) In ANCA-associated vasculitides, the REMAIN study conducted by EUVAS recently demonstrated the superiority of a 4-year maintenance treatment compared to a 2-year treatment Biological monitoring will include a regular complete blood count, platelets, and transaminases (AST or ALT), every week for the first month, then every month for 3 months, then every 3 months until it is stopped

**Box 5:** Method of administering methotrexate

In vasculitis, methotrexate is usually prescribed at a dose of 0.3 mg/kg/week, orally or subcutaneously. If the clinical and biological tolerance is satisfactory, the dose may be increased to 20 and then 25 mg/week to reach this dose after 4–6 weeks; that dose will then be maintained until the end of treatment A supplement with folic acid (preferable to folinic acid, which is more expensive), at a dose of 10 mg/week, 48 h after taking methotrexate, is necessary to reduce its potential toxicity, in particular mucous and hepatic toxicity, and improve the therapeutic maintenance level The pre-therapeutic assessment, often already carried out as part of the diagnosis of vasculitis, must include complete blood count, platelet count, liver enzymes, creatinine clearance, and chest x-ray There is no consensus on the monitoring rate after the start of treatment, but biological monitoring every week for 1 month, then every month for 3 months, then every 3 months until stopping it is acceptable Methotrexate is excreted by the kidney, and its use is not recommended if the glomerular filtration rate is less than 30 ml/min (even if the patient is on dialysis, the drug is not eliminated by dialysis), and must be reduced together with a dose reduction (by 7.5–20 mg/week) if the glomerular filtration rate is between 30 and 60 ml/min. Dehydration, stimulated by fever or a diarrheal episode, can be a source of poisoning, especially in the elderly The combination of methotrexate and sulfamethoxazole/trimethoprim increases the risk of hematological toxicity. This combination is not recommended. If prescribed, it should be done with extreme caution and requires close monitoring. In this situation, it is better to offer aerosols of 300 mg of pentamidine every 21–28 days or even atovaquone (750 mg × 2/day) as a prevention of pneumocystosis rather than sulfamethoxazole/trimethoprim When the withdrawal phase of methotrexate is started, a decrease in methotrexate by 5 mg every month is possible at the end of treatment, before it is stopped In the event of an acute infectious episode, discontinuation of methotrexate is recommended temporarily after a discussion with the referring doctor	

An immunosuppressant therapy, preferably cyclophosphamide, is justified as a first-line treatment in these forms, in combination with corticosteroid therapy.

It is administered as an IV bolus:

- Every 2 weeks during the first month (days 1, 15, and 29), then every 3 weeks until remission is obtained, most often after six or even nine boluses.
- At a fixed dose of 500 mg in patients over 65 years old, and up to 600 mg/m^2^ for the first three boluses then 700 mg/m^2^ for the following three (maximum dose of 1200 mg) in other situations depending on age and kidney function (Box 6).

**Box 6:** Method of administering cyclophosphamide

**Precautions before administration**	
Fertility preservation should be ensured, or at least offered to patients, to women of childbearing age as well as to men	
Hydration prior to and during the infusion is essential. It is supplemented by the administration of mesna (off-label and without certainty of its value for doses of cyclophosphamide < 600 mg/m^2^ per bolus), administered during and after the cyclophosphamide infusion:	
One-third of the equivalent dose of cyclophosphamide (in milligrams) by IV bolus at hour 0	
Then two-thirds of the IV dose at the end of the infusion (90th minute)	
And two-thirds of the dose at hour 4, orally	
When cyclophosphamide is delivered orally, mesna can also be administered orally (equivalent daily dose in milligrams, orally—possible off-label use)	
Monitoring of the cyclophosphamide treatment is based on the complete blood count and platelet count, serum creatinine, and the search for hematuria as a minimum:	
Before each infusion	
Every 2 weeks for the first 3 months	
Then monthly if the oral treatment is continued	
If the neutrophils are < 1.5 × 109/L on the scheduled bolus date, the dose will be reduced by 25% or even postponed (trying not to postpone treatment for more than 2 weeks, in which case another therapy should be discussed)	
**Cyclophosphamide administration regimen**	
In patients with normal kidney function under 65 years of age, the recommended regimen is the following: IV bolus of cyclophosphamide prescribed at a dose of 0.6 g/m_2_ on days 1, 15, and 29; then 0.7 g/m^2^ every 21 days (total of six boluses)	
In patients with normal kidney function under 65 years of age, the recommended regimen is the following: IV bolus of cyclophosphamide prescribed at a dose of 0.5 g/m^2^ on days 1, 15, and 29; then 0.7 g/m^2^ every 21 days (total of six boluses)	
In patients over 65 years of age, regardless of kidney function, the recommended regimen is as follows: IV bolus of cyclophosphamide prescribed at a fixed dose of 0.5 g on days 1, 15, and 29, then every 21 days (total of six boluses). The benefit of this regimen has been demonstrated in the prospective CORTAGE trial, providing efficacy comparable to a conventional treatment but with better tolerance. These low doses of cyclophosphamide have not been evaluated compared to rituximab in this population	
In the event of incomplete remission, three additional boluses can be given	
The maximum dose of each bolus is limited to 1200 mg	
After induction therapy with cyclophosphamide, maintenance therapy should be started between 2 and 4 weeks after the last cyclophosphamide bolus, regardless of the maintenance therapy selected	

At the end of the induction treatment, a re-assessment of the vasculitis activity is essential, so as not to consider switching to a maintenance treatment while the vasculitis is still active.

After six boluses (3.5 months of treatment):

- If complete remission is obtained, a so-called maintenance immunosuppressant treatment, preferably azathioprine (orally at a dose of 2–3 mg/kg/day, without exceeding 200 mg/day) or methotrexate (at a dose 0.3 mg/kg/week orally or subcutaneously) will be prescribed as a follow-up and started between 2 and 4 weeks after the last bolus of cyclophosphamide, regardless of the maintenance treatment chosen, for a duration of 12–18 months, compared with the treatment of ANCA-associated vasculitides.
- If the remission is partial, three additional boluses of cyclophosphamide will be given, according to the same regimen (one bolus every 3 weeks; total of 6 + 3 boluses). A new assessment of the disease activity will be made after the ninth bolus.
- If complete remission is obtained, a maintenance treatment will be started (see above).
- If complete remission is not obtained, the oral form of cyclophosphamide may be prescribed until remission (at a dose of 2 mg/kg/day without exceeding 200 mg/day, with a reduction of the dose of 25% in patients over 60 and 50% in patients over 70).
- The use of other therapies, in particular targeted therapies or biotherapies, has not been evaluated on a sufficient number of patients to be able to make recommendations.

In case the disease is refractory to conventional treatments, the use of plasma exchanges to control the inflammatory flare-up and/or of targeted therapies or biotherapies must be discussed with a reference center or a competence center.

##### PAN due to viral hepatitis B infection

The treatment of these forms is based on the combination:

- Of an initial and brief corticosteroid therapy (< 15 days), at a dose of 0.5–1 mg/kg/day of a prednisone equivalent, allowing rapid control of the most severe initial manifestations linked to vasculitis. This oral corticosteroid therapy is possibly preceded by an IV bolus of methylprednisolone at a dose of 7.5–15 mg/kg/day for 1–3 consecutive days depending on the clinical severity, the clinician’s assessment, and the patient’s cardiovascular condition.
- Of specific antivirals: effective antivirals with little or no resistance will be preferred, alone or in combination (mainly entecavir, tenofovir), by analogy and with reference to the treatment of chronic forms of viral hepatitis B.
- Plasma exchanges, aimed at “purifying” the circulating pathogenic immune complexes [exchanges of 60 ml/kg, preferably by the peripheral venous route, 3–4 times per week for 3 weeks, then gradual withdrawal (e.g., three times per week for 1–2 weeks, then twice a week for 2 weeks)].

As a result of the current rarity of PAN linked to HBV, it is strongly recommended to seek the advice of the reference center.

It is only when this strategy fails that the prescription of an immunosuppressant should be considered. The opinion of the reference center is therefore absolutely necessary.

##### Treatment for GPA and MPA

According to the 2009 EULAR recommendations, a distinction is made between:

- Generalized/diffuse forms characterized by:
  - Clear deterioration of the general condition.
  - Kidney impairment.
  - Major, progressive alveolar hemorrhage.
  - And/or impairment to one or more other organs.
- The limited/localized forms, which exclusively concern GPA, defined by restricted impairment on the upper and/or lower respiratory tract (ENT and/or pulmonary involvement without alveolar hemorrhage), without renal or cutaneous involvement, without deterioration of the general condition, and which are not life-threatening (approximately 30% of GPA cases).

The transition from a localized/limited form to a generalized/diffuse form, and vice versa, is possible during the course of the disease.

##### Treatment of limited/localized forms of GPA

Treatment of pure, very limited ENT forms with cotrimoxazole (trimethoprim 160 mg + sulfamethoxazole 800 mg), at the rate of 2 tablets/day, may be considered initially. The duration of the prescription is several months or years. No criteria for stopping treatment have been established. The risk of serious side effects, especially on the skin, must be taken into account.

However, as a result of frequent progression of the disease, this treatment must be followed by immunosuppressant therapy in the majority of patients.

In the secondary progressive or localized forms but justifying a more “aggressive” treatment than cotrimoxazole, the treatment readily combines:

Corticosteroid therapy (Box 7), started in these forms at a dose of 0.5–1 mg/kg/day of a prednisone equivalent, without exceeding 60 mg/day. Bolus administration of methylprednisolone is exceptional in limited/localized forms. After a 3-week treatment of glucocorticoids, they are gradually reduced. There is no internationally validated pattern of reduction. It varies from 6 months (North American protocols) to 18–24 months (European protocols). It is proposed in France to follow, in the absence of a study available with sufficient follow-up, a regimen of reduction whose essential benchmarks are approximately 20 mg/day at 3 months, 10 mg/day at 6 months, and 5 mg/day at 1 year of a prednisone equivalent.

**Box 7:** Prescription procedures for corticosteroid therapy

Corticosteroid therapy is the subject of many discussions on the most suitable regimen The initial treatment usually includes corticosteroid therapy started at a dose of 1 mg/kg/day of a prednisone equivalent, capped, except in certain cases, at 60 mg/day, or even at lower doses of the order of 0.5 mg/kg/day in the event of discreet to moderate manifestations An IV bolus of methylprednisolone can be given for 1–3 consecutive days, usually at a dose of 7.5–15 mg/kg/day (not to exceed 1 g/bolus), depending on severity and the patient’s cardiovascular condition, before following up with oral corticosteroid therapy at a dose of 1 mg/kg/day of a prednisone equivalent. This therapeutic method is to be reserved for clinical situations requiring a rapid therapeutic response. They are not useful in “cold” forms of the disease or when the clinical condition is progressive or not life-threatening or functionally threatening After an initial treatment of 3 weeks at a dose of 1 mg/kg/day of a prednisone equivalent, the corticosteroids should be reduced. There is no internationally validated pattern of reduction. The total duration of corticosteroid therapy varies from 5 to 6 months (North American protocols) to 18–24 months (European protocols). It is proposed in France to follow, in the absence of a study available with sufficient follow-up, a regimen of reduction whose essential benchmarks are approximately 20 mg/day at 3 months, 10 mg/day at 6 months, and 5 mg/day at 1 year of a prednisone equivalent The PEXIVAS protocol offers, after boluses of methylprednisolone and in combination with an immunosuppressant, a regimen of glucocorticoids at a reduced dose, with a rapid reduction in the corticosteroid therapy initially but maintaining a low dose for at least 12 months (see below). This regimen seems particularly worthwhile, but the composite criterion for judging based on mortality and/or chronic terminal kidney disease represents a very “hard” criterion. The hindsight remains modest and we have no information on the rate of minor or major relapses after this reduced-dose regimen	

An immunosuppressant, preferably methotrexate (at a dose of 0.3 mg/kg/week by oral or subcutaneous route) (Box 5), but cyclophosphamide (Box 6) or rituximab (Box 8), can also be used if necessary according to the usual procedures. It is important to remember that azathioprine has never been shown to be effective as an induction treatment for ANCA-associated vasculitis.

**Box 8:** Methods of administering rituximab

**Precautions** Vaccination of patients against influenza and pneumococcus can be useful It is also useful for preventing pneumocystis systematically throughout the entire treatment and in the following months (practically speaking, until immunological reconstitution) **Premedication** Administer about 60 min prior to starting each infusion of rituximab: Methylprednisolone (Solu-Medrol®): 100 mg in a vial of 50 ml of glucose 5% solution to run in 10 min Paracetamol: 1 g by direct intravenous injection Dexchlorpheniramine (Polaramine®): 5 mg by direct intravenous injection **Induction treatment** Rituximab induction treatment is administered by infusion at a dose of 375 mg/m^2^ on days 1, 7, 14, and 21, after premedication performed prior to each infusion First infusion: it is recommended to start infusion at a rate of 50 mg/h; after the first 30 min, the infusion rate may be increased in steps of 50 mg/h every 30 min, until reaching a maximum rate of 400 mg/h Second infusion: initial rate should be 100 mg/h, then increased in steps of 100 mg/h every 30 min, until reaching a maximum rate of 400 mg/h **Maintenance treatment** Once remission has been achieved, infusion of 500 mg of rituximab is administered on days 1 and 15, then every 6 months	

Tracheobronchial forms, namely stenoses, are particularly refractory to conventional treatments and represent today one of the most difficult conditions to manage. The advice of a reference or competence center is highly recommended. When they appear in a context of developing vasculitis, the treatment is usually based on the combination of glucocorticoids according to the regimen described above, and immunosuppressants, preferring methotrexate or cyclophosphamide over rituximab (consensus of experts), and often with an endobronchial procedure to be performed by a team accustomed to this type of care (dilation, placement of stents, local injections).

The management of orbital masses has also been the subject of controversy, particularly regarding the efficacy of rituximab.

In 2013, the recommendations of the GFEV concerning the use of rituximab specified that rituximab could not be recommended as a first-line treatment in patients with granulomatous manifestations being most prominent, in particular orbital masses, threatening the vital or functional prognosis. A recent French retrospective study in 59 patients with an orbital mass reported a response rate to treatment of 52% with cyclophosphamide and 91% with rituximab. Barring a comparison between these therapeutic strategies, these data nevertheless suggest that it is difficult to recommend one strategy more than another in this situation in the absence of a specific prospective study (Table 2).

**Table 2:** Glucocorticoid dosing in the standard and reduced-dose groups of PEXIVAS

Week	Standard			Reduced dose		
	< 50 kg	50–75 kg	< 75 kg	< 50 kg	50–75 kg	< 75–kg
	Pulse	Pulse	Pulse	Pulse	Pulse	Pulse
1	50	60	75	50	60	75
2	50	60	75	25	30	40
3–4	40	50	60	20	25	30
5–6	30	40	50	15	20	25
7–8	25	30	40	12.5	15	20
9–10	20	25	30	10	12.5	15
11–12	15	20	25	7.5	10	12.5
13–14	12.5	15	20	6	7.5	10
15–16	10	10	15	5	5	7.5
17–18	10	10	15	5	5	7.5
19–20	7.5	7.5	10	5	5	5
21–22	7.5	7.5	7.5	5	5	5
23–52	5	5	5	5	5	5
> 52	Investigators’ local practice			Investigators’ local practice		

##### Prescription procedures for corticosteroid therapy

Treatment of mild forms of MPA

- The therapeutic strategy during MPA was oriented, at least in France, according to whether or not there are poor prognosis factors as defined in the 1996 FFS (Box 1).
- In this PNDS, the use of the notion of “severe illness” and “non-severe illness” was preferred to the FFS for adapting the therapeutic strategy. Thus, “severe” manifestations include (list not restricted to these manifestations alone and to be adapted to the clinician’s opinion) specific cardiomyopathy, mesenteric ischemia, severe intra-alveolar hemorrhage with respiratory distress, rapidly progressive kidney failure, specific stroke, impairment of cranial pair, and multiple mononeuropathy with severe motor impairment.
- Non-severe systemic forms warrant corticosteroids alone, without the addition of a first-line immunosuppressant.
- Corticosteroid therapy.
- The initial treatment always includes corticosteroid therapy started at a dose of 1 mg/kg/day of a prednisone equivalent, with a maximum dose of 60 mg/day, except in specific cases, possibly preceded by a bolus of methylprednisolone. The possible reduction patterns are also detailed in (Box 7).
- Immunosuppressants.
- A prospective, randomized, placebo-controlled study recently evaluated the effectiveness of adding azathioprine to the corticosteroid therapy, as of the diagnosis of vasculitis, with a view to sparing and preventing relapses of MPA but also of EGPA and PAN without poor prognosis factors (CHUSPAN2 trial). This study did not demonstrate superiority of azathioprine in these indications.
- Thus, treatment is based on corticosteroid therapy alone as a first-line treatment. An immunosuppressant or immunomodulatory treatment may be prescribed for patients whose MPA is not controlled by corticosteroids alone or if it is necessary to offer a corticosteroid-sparing treatment (to reduce the risk of occurrence of side effects) or in case of intolerance to corticosteroids.
- In situations where immunosuppressant therapy is indicated as a second line:
- In the absence of a severe manifestation, the choice of an immunosuppressant will preferably be focused on azathioprine (orally at a dose of 2–3 mg/kg/day) (Box 4) or methotrexate (orally or subcutaneously at a dose of 0.3 mg/kg/week) (Box 5), for a period of 12–18 months.
- If severe manifestations appear, the choice of immunosuppressant will preferably be cyclophosphamide or rituximab, according to the same methods as the treatment of forms with poor prognosis factors.

However, in many North American and European protocols, the routine combination of corticosteroids and an immunosuppressant or immunomodulator is prescribed in MPA, based on generalized/diffuse forms of GPA.

##### Treatment of generalized/diffuse forms of GPA and MPA

Remission induction treatment proposals in generalized/diffuse forms of GPA and MPA.

**Table:** 

Clinical forms	Kidney function	Immunosuppressant and/or immunomodulator treatment combined with corticosteroid therapy
Form not involving the functional or vital prognosis, short or medium term OR Form involving the functional prognosis, short or medium term (renal impairment)	Creatinine < 350 µmol/l or GFR > 15 ml/min	First line: Cyclophosphamide IV or Rituximab
Form involving short-term functional prognosis (renal impairment)	Creatinine > 350 µmol/l or GFR < 15 ml/min	First line: Cyclophosphamide IV Second line: Rituximab Even a combination of cyclophosphamide IV + rituximab (regiment according to the RITUXVAS protocol) to be discussed on a case-by-case basis Related treatments: Plasma exchanges may be offered on a case-by-case basis
Severe form involving a threat to life in the very short term (severe renal impairment and/or severe alveolar hemorrhage*)	Creatinine > 500 µmol/l or GFR < 10 ml/min	First line: Cyclophosphamide IV Second line: Rituximab Even a combination of cyclophosphamide IV + rituximab (scheme according to the RITUXVAS protocol) to be discussed on a case-by-case basis Related treatments: Plasma exchange could have a beneficial impact on renal survival and the speed of resolution of an alveolar hemorrhage and should be discussed on a case-by-case basis Mechanical ventilation, if necessary
A predominantly granulomatous form with a functional or vital prognosis involved (orbital mass compressing the optic nerve, symptomatic tracheal stenosis)	Regardless of kidney function	First line: Cyclophosphamide IV or Rituximab Second line: Combination of rituximab + methotrexate treatment (unless there is kidney failure, in the absence of scientific evidence) or Cyclophosphamide PO To be discussed with a reference or competence center

*A severe alveolar hemorrhage is defined by the existence of acute respiratory distress requiring high flow oxygen therapy and/or mechanical ventilation.

##### Remission induction therapy

Corticosteroid therapy.

The initial treatment always includes corticosteroid therapy started at a dose of 1 mg/kg/day of a prednisone equivalent, with a maximum dose of 60 mg/day, except in specific cases, possibly preceded by a bolus of methylprednisolone (Box 7).

Immunosuppressants.

The therapeutic strategy during MPA is oriented according to the presence or absence of severe manifestations, the systemic forms without severe manifestations justifying corticosteroids alone; and those with severe manifestations justifying a combination of corticosteroids and immunosuppressants.

On the other hand, during GPA, its significant risk of relapse justifies the prescription in all cases of an immunosuppressant or immunomodulator combined with the corticosteroid therapy, regardless of the severity of the disease. The immunosuppressants which can be used in combination with corticosteroid therapy are indicated in the table above and are:

Cyclophosphamide: the proof of which has been shown in numerous studies. Cyclophosphamide is administered as an IV bolus prescribed at a dose of 500 mg at a fixed dose of 0.6 g/m^2^ on days 1, 15, and 29; then 500 mg at a fixed dose of 0.7 g/m^2^ every 21 days (to total six boluses), depending on renal function and the patient’s age (Box 6). If the assessment carried out after the six boluses shows that the remission obtained is partial, three additional boluses can be given, according to the same regimen (one bolus every 3 weeks; total of 6 + 3 boluses), with a new assessment of disease activity after the ninth bolus.

Rituximab (Box 8): The pivotal RAVE trial published in 2010 obtained marketing authorization for rituximab as an induction therapy for remission during GPA and MPA. This is a study demonstrating the non-inferiority of rituximab compared to orally administered cyclophosphamide for a period of 3–6 months with follow-up with azathioprine. In contrast, rituximab has been shown to be superior to cyclophosphamide/azathioprine in achieving remission in patients with relapsed vasculitis. In this trial, there was no maintenance treatment performed after the four initial infusions. Some centers use the rituximab administration regimen used to treat rheumatoid arthritis (two infusions of 1 g, 15 days apart). Retrospective studies suggest effectiveness comparable to the regimen comprising four weekly injections. The regimen of two infusions has not been validated prospectively for the induction treatment of ANCA-associated vasculitis.

**Methotrexate** (off-label): prescribed orally at a dose of 20–25 mg/week, has shown efficacy comparable to cyclophosphamide, prescribed orally at a dose of 2 mg/kg/day, in the NORAM study published in 2005, as an induction treatment for non-severe forms of ANCA vasculitis, mainly GPA. However, upon discontinuation after 12 months, the relapse rate was higher in the methotrexate group. In this study, no maintenance treatment was offered after 12 months.

As regards granulomatosis with polyangiitis (GPA) and microscopic polyangiitis (MPA), with the goal of harmonizing management of the disease with international practices, the use of the concept of “severe disease” and “non-severe disease” has been preferred to the 2009 FFS for purposes of individualizing therapeutic strategies. Thus, “severe” manifestations are reclassified (list not restricted only to these manifestations and individualized according to the guidance of the clinician): gangrene, retinal bleeding, drop in auditory perception (sensory), mesenteric ischemia, intra-alveolar hemorrhage, red blood cell casts, increase in serum creatinine > 30% or decrease in creatinine clearance > 25%, specific meningitis, spinal lesions (myelitis), specific cerebrovascular accident, cranial nerve impairment, multiple mononeuropathy with severe motor damage. In all cases, a risk–benefit analysis should be assessed on an individual basis, taking into account, on the one hand, the age of the patient, and, on the other, his or her comorbidities.

- Intravenously administered cyclophosphamide is preferentially used in cases of:
- Presence of associated anti-glomerular basement membrane (anti-GBM) antibody.
- Severe alveolar hemorrhage requiring mechanical ventilation (patients excluded from the RAVE trial).
- Rapidly progressing renal insufficiency with a serum creatinine > 350 μmol/l (patients excluded from the RAVE trial).
- Situations of failure or incomplete response to rituximab.
- Forms with a granulomatous predominance (essentially tracheal and/or bronchial stenoses, functional or life-threatening impairments).
  Rituximab is preferentially used in cases of:
- Patients in relapse or who have already received at least one cycle of cyclophosphamide.
- Situations of failure or incomplete response to cyclophosphamide.
- Women of childbearing age.
- Children and adolescents.
- History of cancer or blood disorders.
- Methotrexate may be used basically in cases of localized rhinosinusitis involvement and/or milder asymptomatic subglottic stenosis of GPA but without renal involvement.
- In relapsing disease with severe renal insufficiency (serum creatinine > 350 µmol/l or GFR < 15 ml/min), a regimen combining rituximab at a dose of 375 mg/m^2^/week for 4 consecutive weeks, in combination with cyclophosphamide at a dose of 15 mg/kg during the first and third infusions of rituximab, may be considered in order to decrease the cumulative dose of cyclophosphamide, but its use as a first-line therapy is not advisable.

Among patients who have not responded sufficiently well to induction treatment with cyclophosphamide or rituximab, the RAVE study has shown that administering the drug not used as a first-line treatment has resulted in achieving remission in the majority of cases.

These files should be discussed at the referral centers or centers for specialized care, in particular in order to ensure the persistence of vasculitis activity and the absence of intercurrent complications.

A refractory form is defined, according to the recommendations of the European League Against Rheumatism, by:

- Acute vasculitis without a decrease in activity after 4 weeks of treatment, properly administered.
- Or the absence of response, as defined by the lack of reduction on the Birmingham Vasculitis Activity Score (BVAS, Additional file 3) of more than 50% at 6 weeks.
- Or the persistence of active disease in at least one vital organ or in three minor sites after more than 12 weeks of treatment, properly administered.

Finally, cyclophosphamide given by mouth is equally effective, at the usual dose of 2 mg/kg/day (maximum dosage of 200 mg/day), but is more toxic because the cumulative dosage quickly rises higher than when given intravenously. Oral administration is therefore reserved for situations of failure when cyclophosphamide is given intravenously and rituximab; in other words, as a third-line treatment.

Among the forms occurring in patients older than 65 years of age, certain precautions or individualized adjustments in dosage must be taken into account (Box 9).

**Box 9:** Elderly subjects

There is no existing age limit that has been agreed upon, to define the “elderly” population. As regards vasculitis, an age upwards of 65, however, very often defines “elderly subjects” In regard to induction treatment for vasculitis, no data in the literature allow us to favor rituximab over cyclophosphamide in this population. Nevertheless, whatever the treatment that may be chosen, the risk of infection is higher among subjects older than 65 years of age who are receiving high doses of corticosteroid therapy and a conventional immunosuppressant. Thus, rituximab or cyclophosphamide may be prescribed as induction treatment for remission after 65 years of age **Regimen for administering cyclophosphamide** Among elderly patients older than 65, whatever their renal function, the regimen recommended is as follows: cyclophosphamide given by intravenous bolus, prescribed at a dose of 0.5 g as a fixed dose on days 1, 15, and 29; then every 21 days (for a total of six boluses). The value of this regimen has been demonstrated in the CORTAGE prospective trial, which showed an effectiveness comparable to conventional treatment, but with improved tolerance **Regimen for administering rituximab (RTX)** Among elderly patients older than 65, the recommended regimen is the same as for patients who are younger than 65 RTX has not been specifically evaluated in prospective studies in this population, but retrospective studies show good tolerance	

Mycophenolate mofetil has been studied in two small controlled trials on induction treatment in comparison with cyclophosphamide, in a total of 75 patients suffering from MPA who have moderate renal compromise. Remission was achieved in three-quarters of patients, without showing a significant difference in the group treated with cyclophosphamide, but the small number of study subjects limits the extent to which it can be interpreted.

The MYCYC European trial is an open, randomized, multicenter study, initiated in 2006 and includes 140 patients with newly diagnosed anti-neutrophil cytoplasmic antibody (ANCA)-associated vasculitis. Patients were randomized between mycophenolate mofetil, at a dose of 2 g/day, with the possibility of increase to 3 g/day if necessary, and cyclophosphamide, administered in the form of an intravenous bolus. When remission was achieved between 3 and 6 months, they were switched to azathioprine in both study arms. Results showed that mycophenolate mofetil is not inferior to cyclophosphamide for inducing remission, but it is followed by a very significant relapse rate, particularly among patients with anti-PR3 ANCA at the time of induction treatment.

Mycophenolate mofetil is not indicated as a first-line induction treatment, or even as maintenance treatment, but it remains a possible alternative if any of the other treatments cannot be used under very specific situations, namely in cases of anti-MPO ANCA, upon guidance by a referral center or center for specialized care.

Elderly subjects.

##### Remission maintenance treatment

- At the completion of induction treatment, re-evaluation of vasculitis, with testing for signs of activity, is indispensable, to avoid moving to maintenance treatment while the vasculitis is still active.
- Once remission has been achieved, induction treatment (cyclophosphamide, rituximab, or methotrexate) should be switched to rituximab, which has been shown to be superior in comparison with azathioprine.
- Maintenance treatment is indicated *in all cases* of GPA, by virtue of the elevated risk of relapse. For cases of MPA, maintenance treatment relies either on corticosteroid therapy alone or on rituximab in systemic forms with “severe” manifestations.
- Some years ago, prior to the publication of the MAINRITSAN trial in 2014, azathioprine (2 mg/kg/day by mouth) or methotrexate (0.3 mg/kg/week), which were given by mouth or subcutaneously and started 15–21 days after the last bolus of cyclophosphamide, were the drugs of choice to prevent relapse, both these drugs showing identical effectiveness. The length of time usually recommended for this so-called maintenance immunosuppressant treatment was 18–24 months, but the REMAIN study conducted by the European Vasculitis Society (EUVAS) has recently shown that maintenance treatment of 4 years is superior to 2 years of treatment. Mycophenolate mofetil, in contrast, is less effective than azathioprine for preventing the occurrence of relapse, according to the data shown in the IMPROVE study.
- Since the results of the MAINRITSAN trial, rituximab has been shown to be the maintenance treatment of choice for relapse prevention. This study has clearly shown, after induction treatment with cyclophosphamide, the superiority of rituximab at a dose of 500 mg every 6 months for 18 months compared to azathioprine. After 28 months, the major relapse rate with rituximab was 5% versus 28% in the azathioprine arm.

Following the results of the MAINRITSAN trial, the US Food and Drug Administration (FDA) approved marketing authorization for rituximab on October 19, 2018, and the European Medicines Agency did the same on November 15, 2018, as “follow-up treatment for granulomatosis with polyangiitis and microscopic polyangiitis in remission,” using a regimen and at dosages recommended by the MAINRITSAN study.

- On the basis of the induction treatment chosen, the first infusion in the maintenance treatment with rituximab usually starts:
- In the month following the last bolus of cyclophosphamide administered as an induction treatment; 4–6 months after the first infusion of induction treatment for rituximab.

**Fig. 1:**
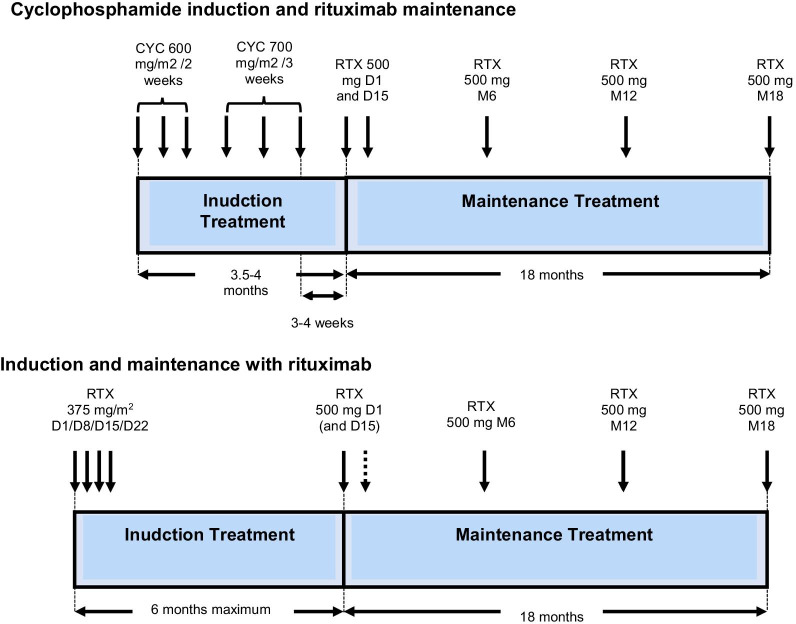
Maintenance treatment

After maintenance treatment has started, the validated regimen consists of administering five infusions over 18 months at a fixed dose of 500 mg of rituximab on days 1 and 15, and months 6, 12, and 18 (Fig. 1).

The frequency of administration of rituximab, as well as its dosage, has been established in an arbitrary way in the MAINRITSAN protocols. The results of the MAINRITSAN2 protocol, which evaluated a 6-month administration of rituximab versus individualized administration based on CD19 levels and/or anti-neutrophil cytoplasmic antibody (ANCA) titers, show identical relapse and tolerance rates in both arms. In contrast, the median number of infusions of rituximab in the “6-month administration” arm was five versus three in the “individualized administration” arm. In the absence of long-term follow-up data, individualized administration has not, at least at this point, been validated as a first-line maintenance therapy.

**Fig. 2:**
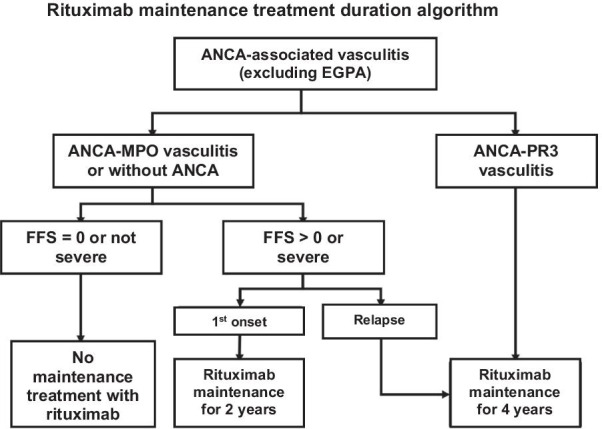
Rituximab maintenance treatment duration algorithm

Long-term follow-up data (60 months) from the MAINRITSAN trial have shown that the absence of a return to normal of ANCA under maintenance treatment was associated with an increased risk of relapse in the group of patients with anti-PR3 ANCA. These days, data from MAINRITSAN3 point in the direction of treatment for a total 4 years for PR3-ANCA-associated vasculitis and initially severe and relapsing MPO-ANCA-associated vasculitis, and 2 years for newly diagnosed severe MPO-ANCA-associated vasculitis (Fig. 2).

Among patients who have relapsed numerous times, the length of maintenance treatment may be continued beyond 2 or 4 years, perhaps in an even more extended way, after discussion with the referral center or center for specialized care, based on the risk–benefit balance (lymphocytopenia, hypogammaglobulinemia, etc.) and on ongoing studies.

Finally, among dialyzed patients following renal involvement of vasculitis, the benefit of maintenance treatment continues to be a subject of debate. A therapeutic trial is currently ongoing (MASTER-ANCA trial) to evaluate the risk–benefit ratio of maintenance treatment in this population.

##### Use of biosimilar agents of rituximab in induction or maintenance treatment

The National Drug and Health Products Safety Agency (ANSM) indicates that when the product of reference, already sold on the market, has been granted authorization for sale and marketing for several therapeutic indications, it is necessary that a biosimilar product show evidence of safety and effectiveness for all indications claimed, without the biosimilar agent being required to follow the complete stages of development of the original medication. In certain cases, extrapolation of therapeutic similarity in one indication to other indications of the product of reference is accepted if clinical experience, the publication of data, or, more generally, the mechanism of action of the drug allows it. ANCA-associated vasculitis fits this last definition.

Biosimilar agents of rituximab may also be prescribed according to the same regimen as the original drug for ANCA-associated vasculitis.

It is important to recall that there are information brochures intended for patients receiving biosimilar drugs that inform them about the risks of biosimilar agents of rituximab, particularly those linked to the risk of progressive multifocal leukoencephalopathy.

##### Treatment of eosinophilic granulomatosis with polyangiitis (EGPA) (formerly called Churg–Strauss syndrome)

It is recommended that induction treatment be adapted to the 1996 FFS prognosis score (Box 1).

###### Corticosteroid therapy

Initial treatment always includes a corticosteroid started at a dose of 1 mg/kg/day of prednisone equivalent, with a maximum dose of 60 mg/day, except in specific cases, possibly preceded by an intravenous bolus of methylprednisolone, depending on the severity and cardiovascular condition of the patient (modalities shown in Box 7).

After an initial treatment of 3 weeks at a dose of 1 mg/kg/day of prednisone equivalent, corticosteroids should be reduced. It has been proposed in France that doctors follow, in the absence of available studies, a tapering-off schedule between 12 and 18 months, of which the essential reference markers are around 20 mg/day at 3 months, 10 mg/day at 6 months, and 5 mg/day at 1 year of prednisone equivalent.

To the extent that patients frequently exhibit cortico-dependent asthma prior to the appearance of eosinophilic granulomatosis with polyangiitis (EGPA), asthma often reappears when corticosteroid therapy is reduced below a certain threshold, which varies from one patient to another, but generally falls between 5 and 10 mg/day of prednisone equivalent. The dosage threshold below which it becomes necessary to resume inhaled corticosteroid therapy is on average 8 mg/day, in the different series reported by the French Vasculitis Study Group. However, the emergence of therapies targeting anti-IL-5 should probably modify the dosage of maintenance corticosteroid therapy.

##### Immunosuppressants

The therapeutic strategy for EGPA is guided by the presence or absence of factors related to poor prognosis defined by the 1996 FFS. Systemic forms with FFS score = 0 justify the use of corticosteroids alone, and those with FFS score ≥ 1 justify a combination of corticosteroids and immunosuppressants. Adaptation of therapeutic protocols to the 1996 FFS thus has allowed patients with a severe form of EGPA to receive more intensive treatment, leading to a survival rate which becomes comparable to that of patients without poor prognosis factors.

Non-severe forms of EGPA (FFS = 0).

Immunosuppressant treatment is not justified as a first-line treatment for these forms. It is only prescribed for patients whose EGPA has not been controlled by corticosteroids alone (persistent disease or relapse) and if it is necessary to prescribe corticosteroid-sparing treatment in cases of corticosteroid dependence of more than 7.5–10 mg/day of prednisone equivalent (to reduce the risk of the appearance of adverse effects), or in cases of intolerance to corticosteroids.

For the record, CHUSPAN2, a randomized, placebo-controlled, prospective trial, recently showed that adding azathioprine, once a diagnosis of EGPA without poor prognosis factor has been raised, does not provide any benefit to patients in terms of achievement of remission, duration of remission, relapse rate, exacerbation of asthma or rhinosinusitis, nor corticosteroid-sparing effect.

In situations where immunosuppressant treatment is indicated as a second-line therapy:

- In the absence of signs of severity (FFS = 0), the choice of immunosuppressant preferably focuses on azathioprine (orally at a dose of 2–3 mg/kg/day) (Box 4) or methotrexate (orally or subcutaneously at a dose of 0.3 mg/kg/week) (Box 5), for a period of 12–18 months, by analogy with the treatment of ANCA-associated vasculitis. The prescription of mycophenolate mofetil for EGPA has not been evaluated and requires, on a case-by-case basis, guidance from the referral center and/or center for specialized care.
- If signs of severity appear (FFS ≥ 1), the choice of immunosuppressant preferably focuses on cyclophosphamide, according to the same modalities as those used for the treatment of forms with poor prognosis factors at the time of the initial diagnosis, as described below.

Forms with poor prognosis factor(s) (FFS ≥ 1).

Immunosuppressant treatment, preferably with cyclophosphamide, is justified as a first-line choice for these forms, in combination with corticosteroid therapy.

It is administered:

- Intravenously every 2 weeks during the first month (days 1, 15, and 29), then every 3 weeks until remission is achieved, more often after six boluses in total
- At a dose of 500 mg at a fixed dose among elderly patients older than 65, and to a dose of 600 mg/m^2^ then 700 mg/m^2^ (maximum dose of 1200 mg) depending on age and renal function (Box 6)

In cases of disease refractory to conventional treatments, the use of plasma exchange to control inflammatory flare-ups and/or other therapies should be the subject of a discussion with the referral center or the center for specialized care.

At the conclusion of induction treatment, re-evaluation of vasculitis, with testing for signs of activity, is indispensable, to avoid moving to maintenance treatment while the vasculitis is still active.

After six boluses (3.5 months of treatment):

- If complete remission is achieved, immunosuppressant treatment, or the so-called maintenance treatment, consisting preferably of azathioprine (orally at a dose of 2–3 mg/kg/day, but not exceeding 200 mg/day) or methotrexate (orally or subcutaneously at a dose of 0.3 mg/kg/week) will be prescribed as the next stage in treatment and started between 2 and 4 weeks after the last bolus of cyclophosphamide, regardless of the maintenance treatment chosen, for a period to run from 12 to 18 months, by analogy to the treatment for ANCA-associated vasculitis.
- If remission is partial, three supplementary boluses of cyclophosphamide will be given, according to the same regimen (one bolus every 3 weeks; a total of 6 + 3 boluses). A new medical evaluation of activity of the disease will be performed at the conclusion of the ninth bolus.
- If complete remission is achieved, maintenance treatment will be started (see above).
- If complete remission is not achieved, the oral form of cyclophosphamide may be prescribed until remission (at the dose of 2 mg/kg/day, without exceeding 200 mg/day).
- The use of other medications, in particular targeted therapies or biotherapies, has not been evaluated among a sufficient number of patients, so as to allow us to formulate recommendations.
- Rituximab is currently under study as an induction treatment for EGPA in the REOVAS study, a controlled prospective trial. The only current data which are available rest on retrospective studies which have suggested that rituximab may have value as an induction treatment for EGPA, particularly with forms showing positivity for ANCA.
- Rituximab is also under study as a maintenance treatment for EGPA in the MAINRITSEG controlled trial. The only current data which is available rests on short-term retrospective studies which suggest that rituximab may have value as a maintenance treatment for EGPA.

##### Asthma and minor cortico-dependent manifestations

Thanks to first-line treatments (see above), control of vasculitis is very often achieved, but in at least 50% of cases, residual asthma and some minor manifestations (ENT, joint pain, asthenia, moderate eosinophilia) persist, all of which could require long-term oral corticosteroids (very often between 5 and 10 mg/day of prednisone equivalent), as long as the vasculitis is no longer active.

Treatment of residual asthma likewise relies on pneumological management, verification of proper compliance using inhaled anti-asthmatic medications (β2-agonists with long-acting effect and inhaled corticosteroids, possibly combined with anticholinergic agents) and management of comorbidities (gastroesophageal reflux with proton-pump inhibitors, chronic rhinosinusitis with washing of nasal cavities, and corticosteroids administered intranasally, etc.). Prescription of leukotriene inhibitors, in cases of associated allergies, may be discussed within the framework of an Asthma Coordination Meeting (ACM).

The value of “conventional” immunosuppressants within this context is today being challenged.

In those cases where cortico-dependent asthma persists, despite inhaled triple therapy and monitoring of comorbidities, use of an anti-IL-5 should be considered after discussion in an Asthma Coordination Meeting.

Mepolizumab, humanized anti-IL-5 monoclonal antibodies, has been prescribed with success in EGPA within the framework of pilot studies. It has allowed control of the disease, above all for permitting the dosage of corticosteroids to be decreased in an acute phase, but with a purely suspensive effect. This biologic (which has been granted an authorization for sale and marketing for severe refractory eosinophilic asthma) has been the subject of a randomized, placebo-controlled, prospective study, directed at preventing relapse within the context of EGPA and maintenance of the disease in remission. Results from this study are positive for primary and secondary criteria, showing that patients on mepolizumab, given subcutaneously at a dose of 300 mg every 4 weeks (in other words, three times the dosage indicated for refractory asthma, which is 100 mg every 4 weeks), relapse less and that the corticosteroid dose may be reduced. New studies will allow us to define the precise place of mepolizumab (as to what the optimal dosage should be) and other anti-IL-5 medications in the treatment of EGPA. In contrast, there does not seem to be a place for omalizumab, regardless of the allergic condition, based on data drawn from observational retrospective studies.

#### Adjuvant treatments and alternative therapies

##### Therapeutic plasma exchanges

Plasma exchanges are indicated in combination with treatment with corticosteroids and immunosuppressants or immunomodulators in cases of polyarteritis nodosa (PAN) linked to HBV, according to the following regimen: exchange of 60 ml/kg of plasma during each session with substitution by albumin at 4% or 5%, 3–4 times per week for 3 weeks, then progressively tapering off (three times per week for 1–2 weeks, then two times per week for 2 weeks).

For ANCA-associated vasculitis, therapeutic plasma exchanges have shown their superiority in comparison with boluses of methylprednisolone in terms of renal survival at 12 months, but not for overall survival, for patients given a diagnosis of serum creatinine greater than 500 µmol/l in the MEPEX trial published in 2007. In this study, seven plasma exchanges (60 ml/kg) were done in 14 days.

The international PEXIVAS trial, whose results were recently published, aimed to discern the value of therapeutic plasma exchanges in a population of 704 patients with ANCA-associated vasculitis, presenting with renal insufficiency defined as eGFR < 50 ml/min/1.73 m^2^ and/or intra-alveolar hemorrhage. Patients were randomized, either in the plasma exchange arm (seven exchanges of 60 ml/kg in 14 days) or in the control arm without the exchanges, then received boluses of methylprednisolone (1.5–3 g). They were randomized once more, assigned either to a corticosteroid group at the standard dose or to a corticosteroid group at a dose reduced by about 60%. Of patients included, 98% exhibited renal insufficiency, while 27% presented alveolar hemorrhage, of which 8–9% were severe.

Results of this trial regarding the value of therapeutic plasma exchange showed the occurrence of death and/or terminal chronic renal insufficiency among 28% of patients in the therapeutic plasma exchange group versus 31% of patients without therapeutic plasma exchange (hazard ratio 0.86; 95% CI 0.65–1.13; *p* = 0.27). None of the subgroup analyses, or analyses differentiated by mortality or the occurrence of terminal chronic renal insufficiency found any significant benefit from therapeutic plasma exchange.

Analysis of patients with severe alveolar hemorrhage seemed, however, to signal a benefit with therapeutic plasma exchange, with a relative risk of reaching the primary composite benchmark (death and/or terminal chronic renal insufficiency) of 0.95 in the absence of hemorrhage, 0.65 in the presence of moderate hemorrhage, and 0.67 in the presence of severe hemorrhage (non-significant difference). Thus, among patients with severe alveolar hemorrhage, of whom the majority also suffered from renal compromise, mortality seemed to be lower in the therapeutic plasma exchange group [11/31 (35%) without therapeutic plasma exchange versus 6/30 (20%) in the group with therapeutic plasma exchange].

Certainly, results from the PEXIVAS trial demonstrated the absence of benefits of therapeutic plasma exchange on mortality and/or the occurrence of terminal chronic renal insufficiency for ANCA-associated vasculitis.

Among the different elements to be considered when interpreting these results, specifically those from the subgroups, it is important to note that:

- A randomized controlled trial was designed to respond to the primary objective of the study (which was in the ambitious PEXIVAS trial a reduction of 35% in the relative risk of the primary benchmark), and the strength of the evidence shown by the results emerging from the analysis of subgroups was always less than evidence for the primary objective.
- Unlike the MEPEX trial, the entire group of patients received boluses of methylprednisolone, and the average overview in relation to therapeutic intervention was over many years, which perhaps could explain certain differences with MEPEX.
- At the present time, we have no information concerning the mode of presentation of renal involvement (acute versus progressive) and the type of histological lesions (the presence of crescents, granulomatous forms, fibrous sequelae upon admission, degree of acute tubular necrosis, etc.), variables which could greatly modify the expected response from therapeutic plasma exchange.
- We cannot exclude the effect of a possible inclusion bias on the results of this trial, given that the researchers were hesitant to randomize a patient whose vital prognosis was at stake, taking into account the prior results of the MEPEX trial.
- There are no data regarding certain severe clinical forms (cerebral vasculitis, myocarditis, etc.).

Thus, even if the use of therapeutic plasma exchange from now on should be reduced, we cannot exclude its usefulness among certain patients, after a case-by-case discussion with the referral center and/or center for specialized care, specifically for:

- Patients who suffer from severe alveolar hemorrhage.
- Patients who have a persistent aggravation of their renal insufficiency, despite conventional treatment with corticosteroids, in combination with cyclophosphamide or rituximab.
- Patients who exhibit rapidly deteriorating glomerulonephritis and/or alveolar hemorrhage without a definitive diagnosis, at least until results of the anti-MBG antibody test and/or a definitive diagnosis are received (with a possible stopping once the diagnosis of ANCA-associated vasculitis has been made).

With regard to certain modalities of performing therapeutic plasma exchange, the substitution of plasma by starches is contraindicated. The substitution by frozen fresh plasma is indicated within 48–72 h around a possible biopsy and/or in cases of alveolar hemorrhage, so as to limit coagulation problems induced by plasma exchange and/or among elderly patients who exhibit weak recovery of their coagulation factors between sessions.

Immunoadsorption, which is another technique of apheresis that allows for adsorption of immunoglobulins on a colony of staphylococcal protein A, may likewise be proposed for this indication. The volume of plasma treated is usually 100 ml/kg per session, without the need for a substitute solute. Immunoadsorption is under investigation for ANCA-associated vasculitis (CINEVAS trial).

##### Intravenous immunoglobulins

Immunoglobulins given intravenously (IVIG) at immunomodulatory doses (2 g/kg/cure) may on rare occasions be prescribed in combination with other specific treatments for patients suffering from refractory ANCA-associated vasculitis (including forms without ANCA). They sometimes provide improvement during an infectious complication, in combination with other specific treatments, for patients exhibiting active vasculitis.

However, there is currently a shortage of IVIG in France and a number of countries, leading doctors to reserve this treatment for patients who have a validated indication and priority needs. The National Drug and Health Products Agency (ANSM) has published prescription recommendations for IVIG in situations of supply limitations, with a hierarchization of indications. Within this context, ANCA-associated vasculitis arising from relapse or resistance or intolerance to corticosteroids and immunosuppressants is not considered to be a priority, and it is necessary that its use (off-label) be discussed with the referral center and/or center for specialized care.

The use of immunoglobulins as immunological substitution, at a dose of 0.4–0.5 g/kg/cure, may be contemplated in cases of ANCA-associated vasculitis with a secondary symptomatic immunological deficit satisfying the following criteria:

- Deficiency in the production of antibodies with immunoglobulin quantitation of IgG < 4 g/l.
- Associated with repeated infections requiring hospitalization.
- After failure of prophylactic antibody therapy with amoxicillin or cotrimoxazole.
- After validation in a multidisciplinary coordination meeting.

In cases involving indications for immunoglobulins at substitutive doses, they may be administered intravenously or subcutaneously.

##### Monoclonal antibodies and other biotherapies

Monoclonal antibodies and/or other biotherapies targeting TNF-α (basically infliximab) or CTLA-4 (basically abatacept) have been evaluated in a limited number of cases.

Infliximab was the subject of a pilot prospective study, the RATTRAP trial. It may be prescribed after discussion with the referral center and/or center for specialized care for patients who have not responded to validated treatments, such as cyclophosphamide, rituximab, and methotrexate (refractory vasculitis). Another drug targeting TNF-α, etanercept, in contrast, did not show effectiveness in a randomized, placebo-controlled trial conducted in the USA (WGET trial) with an unfavorable tolerance profile.

Abatacept was the subject of a non-controlled pilot study with patients who were refractory to conventional treatments, suggesting a beneficial effect. A randomized and controlled prospective study is ongoing.

These biologics are not currently validated for systemic necrotizing vasculitis (SNV). It is necessary for the prescribing doctor to discuss their prescription with the referral center and/or center for specialized care.

#### Treatment of relapse and refractory forms

Patients who present with a relapse after conclusion of first-line treatment of vasculitis should be treated, except in cases of contraindication, either according to the same therapeutic regimen used as a first-line treatment or with rituximab 375 mg/m^2^/week for 4 consecutive weeks. In the RAVE study, rituximab was found to be more effective than cyclophosphamide for inducing remission among patients treated because of relapse of the disease. Thus, a patient receiving cyclophosphamide in induction treatment preferably should receive rituximab to treat instances of relapse.

Refractory forms are defined according to the recommendations of the European League against Rheumatism:

- Active and progressing disease which does not respond after 4 weeks of conventional treatment.
- Absence of response, defined as a reduction of ≤ 50% on the vasculitis activity scale (BVAS), after 6 weeks of treatment.
- Persistent chronic disease, defined as the presence of at least one major element or three minor elements on the list of items on the activity scale of the disease (e.g., BVAS or BVAS/WG) after at least 12 weeks of treatment.

Guidance by the referral center and/or center for specialized care is required in cases of refractory forms, as well as in cases of:

- Relapse occurring during the course of, or rapidly arising, after first-line treatment.
- Contraindication for immunosuppressants classically used as first-line drugs (cyclophosphamide in particular).
- Multiple instances of relapse.
- Discussion of therapeutic alternatives with polyvalent immunoglobulins, therapeutic plasma exchanges, monoclonal antibodies, or other biologics.

#### Treatments combining other medications

##### Treatments combining extended corticosteroid therapy

Classic measures used in combination with the prescription of an extended corticosteroid therapy should be applied (hygiene and dietary rules, supplementation with potassium, prevention of corticosteroid-induced osteoporosis, possible prescription of proton-pump inhibitors in cases of strong doses of corticosteroids and/or history of ulcers, etc.), according to recommendations currently in effect (Box 10).

**Box 10:** Nutritional regimen with corticosteroids

A salt-free and/or sugar-free diet has not been scientifically shown to be of value in the fight against the secondary effects of corticosteroid therapy **What has been shown** Need for a sufficient intake of vitamin D and calcium: 1 g of calcium and 800 IU of vitamin D per day Limitation of overall calorie intake if one wishes to avoid or limit corticosteroid-induced weight gain; *it is important to advise patients to avoid snacking outside regular meals* **This is often done with a weak level of evidence** A low-salt diet has been periodically recommended. The purpose is to limit an increase in arterial pressure. The majority of physicians only agree that a salt-free diet may turn out to be more harmful than beneficial, specifically among the elderly or for doses lower than 15–20 mg/day of prednisone equivalent Potassium supplement: at high doses (bolus, prednisone ≥ 1 mg/kg/day), and in cases of potassium-reducing medications given in combination (guidance by a nephrologist if GFR < 30 ml/min), a supplement may be required **The following does not rely on any evidence** A low-sugar diet with an elevated glycemic index in order to limit the risk of corticosteroid-induced diabetes; other risk factors (dosage, family history, obesity, age) are more significant A protein-rich diet to limit corticosteroid myopathy; above all, it is necessary to recommend physical exercise All in all, balanced nutrition and sufficient physical exercise seem to be the best prescription	

Nutritional regimen with corticosteroids.

The prevention of osteoporosis should be systematically implemented when beginning corticosteroid therapy for more than 3 months, regardless of the dosage: recommendations by learned societies, such as the French Societies of Rheumatology and Internal Medicine, may be followed.

General measures are as follows:

- Do not use high doses of corticosteroids over an excessively long time.
- Test for, prevent, and/or treat:
- Other risk factors for osteoporosis.
- Risk of falling.
- Insufficient calcium intake or intake of vitamin D. Calcium deficiencies may be treated by individualizing nutrition and/or drug supplementation; intake of vitamin D should be 800 IU/day or an ampule of 100,000 IU, to be administered every 2–3 months.

Bone mineral density (BMD) measurements are indicated, as well as measurement on the FRAX score (if the patient is older than 50 years of age).

Indications for anti-osteoporosis treatment are as follows (one of these situations is sufficient):

- History of fracture after 50 years of age.
- T-score ≤ − 2.5 of the spine or femur.
- Age ≥ 70 years old.
- Prolonged corticosteroid therapy and/or high doses (≥ 7.5 mg/day of prednisone equivalent for > 3 months).
- Risk of facture evaluated on the FRAX score higher than the risk expected due to age.

In cases of severe osteoporosis with at least two vertebral fractures, treatment with teriparatide (PTH 1–34), a logical choice by virtue of the decreased formation of bone linked to corticosteroid therapy) is recommended and reimbursed for a period of 18 months.

Treatment with bisphosphonates is systematically recommended for purposes of prevention in cases of doses ≥ 7.5 mg/day of prednisone equivalent, anticipated for more than 3 months or ongoing after more than 3 months in menopausal women and men older than 50 years of age.

Risedronate and zoledronic acid have received authorizations for sale and marketing and are reimbursed for this indication. Doses should be adjusted in cases of reduced GFR and, upon the advice of a nephrology specialist, should be requested if GFR < 30 to 40 ml/min/1.73 m^2^ (bisphosphonates are contraindicated in cases of renal insufficiency with GFR < 30 ml/min/1.73 m^2^ for oral forms and 35 ml/min/1.73 m^2^ for zoledronic acid). Zoledronic acid finds a place of choice here, when taking into account the length of time its effect lasts (up to 5 years). A single intravenous infusion, which may be done at home, will thus cover the entire length of corticosteroid therapy and will avoid problems frequently observed with oral bisphosphonates, above all among these patients who are often polymedicated. It is necessary to safeguard the patient’s dental condition and to prevent patients from experiencing the occurrence of pseudo-influenza symptoms 48 h after infusion among 30% of patients.

Prescription of bisphosphonates is contraindicated for pregnant women and treatment with risedronate should be given priority for women of childbearing age (by virtue of its effect, which remains for less than 1 year).

If there is no indication for treatment with bisphosphonates, another follow-up bone densitometry test may be done after 2 years of monitoring, in order to re-evaluate this indication in cases where bone mass has fallen.

##### Prevention of infections

Prevention of infections relies, on the one hand, on the implementation of drug prophylactic measures and on bringing vaccinations up to date (see below), and, on the other, periodic clinical and biological monitoring (and the adjustment of immunosuppressant doses, if necessary).

In regard to the prevention of infections by *Pneumocystis jiroveci*, there is no international consensus for these autoimmune diseases. This should be discussed according to the predisposing factors, lymphocyte count, and type of treatment administered. However, prophylactic treatment may be recommended in cases of:

- Treatment with cyclophosphamide (induction treatment).
- Treatment with rituximab (induction and maintenance treatment).
- Treatment, whatever the immunosuppressant prescribed, if CD4^+^ T lymphocytes are < 300/mm^3^.

This relies on a prescription of:

- Trimethoprim 80 mg/day + sulfamethoxazole 400 mg/day (or trimethoprim 160 mg + sulfamethoxazole 800 mg three times a week, for which tolerance is slightly less good); the dosage should be adjusted in the event that there are changes in renal function.
- In cases of allergies to sulfonamides, or if the patient is taking methotrexate: pentamidine aerosols (300 mg/dose) every 3–4 weeks or atovaquone by mouth (1500 mg/day in two doses) (off-label).

Trimethoprim + sulfamethoxazole as preventive treatment may be given until lymphocyte reconstitution (e.g., on the order of 6 months after the last infusion of maintenance rituximab).

It should be noted that a recent retrospective study has shown that prophylaxis by cotrimoxazole likewise reduces the risk of severe infections overall during treatment with rituximab in regard to ANCA-associated vasculitis.

Among patients with a history of untreated or spontaneously cured tuberculosis, or who have had recent contact with someone with tuberculosis, or with proven latent tuberculosis (primary infection), treatment for latent tuberculosis infection may be proposed by virtue of an infectious investigation to be done (tuberculin test, chest x-ray, immunological test to detect the production of IFN-γ). This relies on a dual therapy (rifampicin 10 mg/kg/day + isoniazid 4–5 mg/kg/day, taken in a daily dose on an empty stomach for 3 months; or rifampicin 300 mg + isoniazid 150 mg, 2 capsules/day taken in a single daily dose on an empty stomach for 3 months). Isoniazid by itself, at a dose of 4–5 mg/kg/day over 9 months, is an alternative in cases of contraindication or toxicity due to rifampicin, or among patients with cirrhosis. Doses of corticosteroids may be increased by 20–30% in cases of concomitant prescription of rifampicin (liver enzyme induction).

Among patients who have had prior contact with HBV (a population characterized by a positive test for anti-HBc antibodies) and even more so in cases of chronic hepatitis B, the risk of viral reactivation exists with corticosteroids and/or immunosuppressants. Monitoring of the virus and of liver symptoms at 1 month after treatment has been started, then every 3 months, is required, as well as guidance by a specialist in hepatology, in order to discuss implementation of preemptive treatment.

In cases of immunosuppressant-induced hypogammaglobulinemia, the question of whether to implement substitution by immunoglobulins intravenously or subcutaneously is a significant issue. It is important to recall that no study has demonstrated that a drop in immunoglobulins is a risk factor for infection among patients suffering from ANCA-associated vasculitis, specifically treated with rituximab.

However, there is currently a shortage of IVIG in France and a number of countries, leading doctors to reserve this treatment for patients who have a validated indication and priority needs. The National Drug and Health Products Safety Agency (ANSM) has published recommendations regarding prescription of IVIG in situations of supply limitations, with hierarchization of indications (http://ansm.sante.fr/Dossiers/Medicaments-derives-du-sang/Recommandations-d-utilisation-des-MDS-en-situation-de-tension-d-approvisionnement/(offset)/1).

Within this context, the use of IVIG at a substitutive dose is only contemplated in cases of systemic vasculitis accompanied by secondary symptomatic immunological deficit, which satisfies the following criteria:

- Deficiency in the production of antibodies with immunoglobulin quantitation of IgG < 4 g/L.
- Association with repeat infections requiring hospitalization.
- After failure of prophylactic antibiotic therapy with amoxicillin or cotrimoxazole.
- After validation in a multidisciplinary coordination meeting.

In cases involving indication of immunoglobulins at substitutive doses, they may be administered intravenously or subcutaneously.

##### Vaccinations

It is recommended that vaccinations be brought up to date as quickly as possible after an autoimmune disease is diagnosed and at least 15 days before starting immunosuppressant treatment for attenuated live vaccines. Vaccine immunogenicity appears, however, less effective on rituximab and methotrexate in particular. It is recommended to bring the vaccine card up to date, to have a seasonal flu vaccine and anti-pneumococcal vaccine as soon as possible, starting with the induction phase and, if possible, at least 15 days before the introduction of immunosuppressants.

The risk of reactivation of an autoimmune or inflammatory disease after vaccination is a risk which remains theoretical and which must be balanced against the real risk of infection or reactivation.

Attenuated live vaccines are contraindicated among persons receiving immunosuppressant treatment, biotherapy, and/or corticosteroid therapy at a dose > 10 mg/day of prednisone equivalent or in the form of a bolus.

After treatment is stopped, the minimum waiting period for vaccination is 3 months (6 months for rituximab).

Annual anti-influenza and anti-pneumococcal vaccination is highly recommended. The High Public Health Council recommended, in effect, as of 2012, a schedule of so-called prime-booster shots, in combination with vaccination, using 13-valent conjugate virus vaccine (Prevenar®), to be followed at least 8 weeks later by a 23-valent polysaccharide vaccine (Pneumovax®).

Evaluation of innovative anti-pneumococcal vaccine strategies in order to obtain better immunogenicity when taking rituximab is currently under study within a protocol framework (PNEUMOVAS trial).

Finally, indications for vaccination continue to be a subject of dispute among patients suffering from EGPA. This has been contraindicated for a long time by virtue of flare-ups occurring after vaccination or desensitization. Nevertheless, the risk of serious infectious diseases in immunosuppressed patients argues in favor of vaccination. We are therefore recommending this for these types of patients, but, as a precaution, it is desirable to avoid administering vaccinations to patients who are experiencing flare-ups.

##### Prevention of sterility and risk of teratogenicity

Certain immunosuppressants, in particular cyclophosphamide, present risks for inducing sterility (gonad toxicity) or teratogenicity, requiring observance of the precautions for use from the Summary of Product Characteristics (SPC). An effective method of contraception is necessary, in particular with chlormadinone (1 capsule/day continuously) or luteinizing hormone-releasing hormone (LHRH) analogues (triptorelin, Decapeptyl), which could be proposed to preserve female fertility (off-label).

The effectiveness of LHRH analogues has been confirmed by two randomized, placebo-controlled trials and one meta-analysis, which includes 12 trials. However, another study has shown that the protective effect of gonadotropin-releasing hormone (GnRH) analogues on ovarian reserve may be limited to 1–2 years after chemotherapy and that it does not exist beyond 5–7 years after treatment is stopped. These data must be taken into account in order to preserve the female fertility of very young patients.

The risk to fertility in the latter phases of immunosuppressant treatment, in particular from cyclophosphamide, depends on the ovarian reserve of the patient and on her age and must be evaluated by the level of the anti-Müllerian hormone (AMH), which is a good quantitative marker.

Cyclophosphamide permanently alters the ovarian reserve depending on the dose, length of treatment, and age of the patient. While young prepubescent girls appear to be relatively protected, the risk of irreversible amenorrhea appears in adolescence and increases with age: 12% in cases of treatment before 25 years of age; 27% from 26 to 30 years; 62%, starting at 31 years of age. The cumulative dose responsible for premature ovarian insufficiency among 50% of females decreases with age: 20 g at 20 years old, 9 g at 30 years old, and 5 g at 40 years old. Amenorrhea appears about 4 months after the start of treatment.

Thus, in cases where cyclophosphamide is used, it is advisable to perform cryopreservation of sperm for men. For women, it is advisable to contact a fertility preservation center in order to consider the most effective approach, depending on the time available: cryopreservation of eggs/oocytes or embryos, treatment with LHRH agonists.

The best prevention against the risk of sterility also rests today on the prescription of rituximab instead of cyclophosphamide for women in their period of sexual activity and among men who wish to have children and for whom cryopreservation of sperm cannot be done or has failed. For patients who must take cyclophosphamide, reducing the length of treatment and the total dose administered is the recommended approach.

##### Risk of cancer

The prolonged prescription of immunosuppressants is associated with an increase in the risk of certain cancers, specifically cancer of the bladder, with cyclophosphamide, but also certain cancers of the skin and/or certain malignant blood cancers, in particular with the use of azathioprine. Prevention relies in particular on regular and extended clinical monitoring of patients, periodic evaluation to adjust prescribed treatments, mesna prescription (in the absence of allergies or contraindication) during use of cyclophosphamide, quitting tobacco use, and screening for skin and gynecological cancers.

It is possible that the longer use of rituximab in comparison with cyclophosphamide leads to a decrease in the future risk of cancer.

##### Prevention of cardiovascular risk

After the first year of monitoring, cardiovascular complications represent the first cause of death for ANCA-associated vasculitis. Moreover, there is an increased risk of subclinical atherosclerosis among patients suffering from systemic necrotizing vasculitis (SNV), in comparison with a control group selected for classic cardiovascular risk factors. Thus, the risk of major cardiovascular events is increased by a factor of 3 among patients suffering from SNV.

Patients who have SNV should be the subject of in-depth and regular screening of cardiovascular risk factors, in order to modify the management of their therapy, consistent with recommendations for the usual good practices.

It is likewise important to recall that the primary risk factor for major cardiovascular events among these patients is long-term corticosteroid use at high doses, justifying the reduction or even the stoppage of corticosteroids as quickly as possible.

Among patients without a formal indication for the prevention of cardiovascular risk due to lipid-reducing drugs, the value of treatment with statins as primary prevention against cardiovascular complications is currently under study within a protocol framework (STATVAS trial).

While waiting for results from that trial, the recommendations by French National Health Authority (HAS) to be applied are:

- As primary prevention between 40 and 65 years of age, the European SCORE tool is used (allowing evaluation of the risk of death due to cardiovascular origin at 10 years, which takes into account sex, age, tobacco use, cholesterol level, and arterial blood pressure). The SCORE tool cannot be applied prior to 40 years of age or after 65 years of age.
- Lifestyle advice: encourage quitting the use of tobacco, prevent second-hand exposure to tobacco, “strongly” discourage the consumption of alcohol, encourage an individualized nutrition plan to allow participation aimed at reducing cardiovascular risk and improving lipid profile, recommend physical activity in the fight against a sedentary lifestyle, with the goal of attaining a total of 30 min of exercise several days per week and at least 150 min per week of moderate physical activity.
- Among elderly subjects, it is recommended that the existence of risk factors, comorbidities, potential adverse effects, the expected benefits of treatment, the presence of fragility, and choices of the patient all be taken into account.

#### Specific cases

##### Chronic renal insufficiency

In cases of decreased glomerular filtration rate:

- Doses of calcium intake and the prescription of vitamin D_3_ should be adjusted according to KDIGO recommendations (www.kidney.org/professionals/kdoqi/guidelines).
- Bisphosphonates should be prescribed with caution and are contraindicated when GFR < 30 ml/min/1.73 m^2^.
- Doses of cyclophosphamide should be adapted to the GFR (Box 6).
- The use of methotrexate is not recommended if GFR < 30 ml/min, and care should be taken if the GFR falls between 30 and 60 ml/min (Box 5).
- Nephroprotective treatment should be added to the immunosuppressants (angiotensin-converting enzyme (ACE) inhibitors), angiotensin receptor II antagonists (ARA2), together with an individualized nutritional diet and management of metabolic consequences of chronic renal insufficiency (see National Diagnostic and Care Protocol (PNDS) Adult Chronic Renal Disease).

##### Pregnancy

The effects of pregnancy on the progression of vasculitis have not been fully established. Management of therapy in cases of the appearance of vasculitis among pregnant women, or in cases of pregnancy occurring while under treatment or toward the end of vasculitis treatment, requires recourse to a center for specialized care or referral center to discuss the best therapeutic approach. The reader may also refer to the Web site of the referral center on teratogenic agents (www.lecrat.fr).

##### Children

Management of therapy in children suffering from SNV requires recourse to a referral center or center for specialized care for pediatric guidance (Additional file 2) for a discussion about the best therapeutic approach, which may differ from that of an adult.

#### Other non-specific drug treatments used in combination

Depending on the individual cases, other drug treatments may/should be used in combination to control manifestations of the disease and/or those linked to treatments.

Classes I, II, and/or III analgesics for treatment of pain and pain attacks, which might require the use of class III analgesics.

Platelet antiaggregation therapy with predisposition of associated cardiovascular risk (elderly subjects, overweight and/or obese individuals, cardiovascular history, organ ischemia, etc.) or by way of symptoms (distal ischemia, Raynaud’s syndrome, etc.) (off-label).

Anti-allergic agents (antihistamines) if it involves allergy and/or asthma with general and/or local means of administration (eye drops, ointments, etc.).

Anti-asthma agents if necessary (EGPA).

Antibiotic therapy targeted in cases of intercurrent infection or as prevention for certain opportunistic infections.

Anticoagulants in cases of arterial or venous thrombosis (curative treatment) or as prevention against situations of risk, in particular during flare-ups of ANCA-associated vasculitis, among patients at risk (nephrotic syndrome, bedridden patients, etc.).

Antidepressants in cases of mood disorders.

Antiemetics, antidiarrheal agents, medications for constipation in cases of digestive disorders induced and/or aggravated by the disease and/or the treatments.

Antihypertensive agent(s) in cases of arterial hypertension.

Oral antidiabetic agents and/or insulin therapy in cases of corticosteroid-induced diabetes.

Antiepileptic agents in cases of epilepsy (by avoiding enzyme-inducing drugs such as carbamazepine).

Antiepileptic agents and/or antidepressants (serotonin and noradrenaline reuptake inhibitors or tricyclics) in cases of neurogenic pain, dysesthesia, cenesthesia, and/or paresthesia.

Eye drops for moistening and washing of the eye.

Local corticosteroids given intranasally among patients suffering from GPA or EGPA with ENT complaints.

Topical corticosteroids, emollients, and wound-healing agents in cases of skin or mucosal lesions.

Immunoglobulins at immuno-substitutive doses in cases of systemic vasculitis accompanied by secondary symptomatic immunological deficit, which satisfies the following criteria:

- Deficiency in the production of antibodies with immunoglobulin quantitation of IgG < 4 g/l.
- Associated with repeat infections requiring hospitalization.
- After validation in a multidisciplinary coordination meeting.

Immunoglobulins at immunomodulating doses are not recommended, except for situations after discussion with a referral center and/or center for specialized care.

ACE inhibitors and/or ARA2 aimed at kidney protection in cases of arterial hypertension and/or proteinuria.

Physiological solution for washing and abundant cleaning of nasal cavities among patients suffering from GPA or EGPA with ENT complaints.

Sleeping pills/hypnotics in cases of sleep disorders linked to pain or if taking corticosteroids.

Statins in cases of dyslipidemia uncontrolled by diet or underlying dyslipidemia or other cardiovascular risk factors which could increase the risk of early atherosclerosis and/or corticosteroid-induced cardiovascular disease. The value of statins as a primary prevention of cardiovascular complications arising from ANCA-associated vasculitis is under evaluation (STATVAS trial).

Blood transfusions and supplementation with iron, folates, and vitamin B_12_ in cases of anemia related to the disease and/or treatments. Correction of anemia, in particular among elderly subjects and/or patients with renal insufficiency, may also make use of erythropoietin (EPO).

Anti-influenza and anti-pneumococcal vaccination by prime boost (13-valent conjugate vaccine, to be followed at least 8 weeks later by a 23-valent polysaccharide vaccine) for patients with SNV. Vaccination safety remains controversial during the active phase of EGPA. (Guidance is needed from a referral center and/or center for specialized care.) Assessment of innovative anti-pneumococcal vaccination strategies in order to achieve better immunogenicity is currently under study (PNEUMOVAS trial). One booster injection with 23-valent polysaccharide vaccine is indicated at 5 years.

Vitamins B_1_, B_6_, and PP may possibly be prescribed in cases of peripheral neuropathy (off-label). Evidence of their effectiveness has not been shown.

### Non-drug treatments and paramedical management done in combination

Surgery if necessary (e.g., in cases of intestinal perforations, amputation in cases of ischemia in the extremities, surgery for tracheal and/or bronchial stenoses, placement of intracavitary pacemakers in cases of conduction disorders, etc.). Nasal plastic reconstruction surgery may be contemplated once a durable remission has been achieved and a sufficient period of time has passed, the minimum period of which is yet to be defined.

#### Therapy education

Extrarenal clearance in the acute phase in cases of acute renal insufficiency with anuria and/or with criteria for emergency hemodialysis.

Chronic extrarenal clearance to be followed by renal transplantation center, if necessary.

Physical therapy, physical medicine and rehabilitation (PMR), to be started early in cases of motor deficit disorders.

Oxygen therapy and assisted ventilation in cases of acute or chronic respiratory insufficiency and/or cardiac failure.

Management by a dietitian (if needed and by virtue of the treatments prescribed and their possible and/or observed consequences).

Management by a psychologist may be needed within the framework of follow-up care normally proposed for all chronic diseases.

Interventional vascular radiology in cases of ruptured aneurysms, stenoses, or in cases of complications arising from invasive techniques (biopsies, placement of catheters, etc.).

Non-invasive mechanical ventilation or after orotracheal intubation in cases of respiratory distress linked to alveolar hemorrhage or pneumopathy, for instance.

Under circumstances linked to the disease or treatment:

- Hearing correction.
- Vision correction.
- Corticosteroid joint infiltration.
- Orthotics or joint braces, in particular foot lifters.
- Care and/or surgery for corticosteroid-induced cataracts.
- Vision therapy (rehabilitation for oculomotor dysfunction).
- Speech therapy (speech and/or cognitive disorder rehabilitation).
- Dental care (infections, cavities, and enamel or gum pathologies, induced by disease or treatments; corticosteroid therapy and/or immunosuppressants).

In cases of progression toward multiple disabilities, it may be necessary to plan for individualization of the layout of daily life (home, vehicle) and the prescription of medical devices (crutches, day and/or night braces, orthopedic shoes, walkers, manual or electric wheelchairs, anti-bedsore mattresses, hospital beds, home oxygen therapy, implantable catheters, needed for infusion through peripheral or central venous means, whether bedside or portable, etc.), as an aid to adapted structures (Department Center for the Disabled (MDPH), specialized centers, etc.), and requests for assistance adaptations:

- Qualification and first request with the MDPH.
- Request for handicapped worker status (RQTH).
- Request for priority-seating and disability parking card (CMI).
- Request for Adult Disability Allowance (AAH) (for patients older than 20 years old), Educational Allowance for Disabled Children (AEEH) (for patients younger than 20 years old).
- Education (test planning, guidance, etc.).
- Disability compensation benefits (PCH), personal assistance (housekeeper).
- Professional retraining.
- Occupational therapy.

For parents of children suffering from SNV, parental leave of absence may be requested.

Patient associations and social assistance may provide guidance, advice, and help for patients as they perform their daily tasks.

## Follow-up care

### Objectives

The primary objectives of follow-up care are:

- To screen and provide early treatment for complications linked to the disease or treatments during the initial phase.
- To screen and manage treatment failures and/or potential relapse early on and in an individualized way.
- To limit, if necessary, to screen, and to manage sequelae linked to the disease (or treatments) early and in an individualized way.
- To limit, if necessary, to screen, and to treat late-onset complications linked to treatments (or to the disease) (atherosclerosis, malignant diseases, risk of infections, etc.) early on.
- To evaluate potential factors involving poor therapy compliance and to correct them.
- To evaluate psychological, familial, and educational consequences and/or social and professional repercussions of the disease and to limit their negative consequences.

For patients undergoing induction treatment, the objectives of this treatment will also be:

- To determine progression of the disease (remission or, on the contrary, aggravation/worsening).
- To limit treatment-related risks.

For patients undergoing maintenance treatment or whose treatment is nearly completed, the objectives will also be:

- To determine progression of the disease (maintenance of remission) and to tailor treatment accordingly (dosage and length of time).
- To screen and treat relapses early on.
- To screen and manage future adverse effects of treatment in the medium and then long term.
- To ensure optimal management of sequelae linked to the disease and/or treatments.

### Healthcare professionals involved

Follow-up of patients suffering from SNV is multidisciplinary and coordinated by a hospital physician collaborating with the treating physician and the referral center and/or center for specialized care.

It may involve, based on the clinical picture, different professionals, in particular:

- Hospital physicians (and possibly private doctors): internists, clinical immunologists, rheumatologists, nephrologists, pulmonologists, pediatricians, neurologists, ENT specialists, hematologists, gastroenterologists, ophthalmologists, geriatric specialists, dermatologists, etc.
- Other specialists (hospital physicians or private doctors) may be required to intervene, generally at the request of the physicians cited above: gynecologists, obstetricians, surgeons, cardiologists, rehabilitation specialists, radiologists, biologists, physicians at pain management centers, where appropriate.
- Generalist physicians.
- Paramedical professionals: nurses, dietitians, speech therapists, orthoptists, physical therapists, psychologists, child psychologists, child psychiatrists, etc.
- Social work professionals.
- Occupational physicians.
- School doctors, if necessary.

Follow-up may also involve patient associations.

### Pace and content of follow-up care

Consultations and systematic examinations are necessary to provide follow-up care of patients. In the event that the disease worsens or there are complications or treatment-related adverse effects, consultations with hospital physicians, private doctors, and/or the treating physician, and/or supplemental examinations may become necessary.

#### Induction treatment

##### Clinical examinations

Follow-up clinical examinations are identical to the initial evaluation and are performed by a hospital physician of the center responsible for managing the patient’s care. An examination is performed during each infusion in cases of treatment with cyclophosphamide or rituximab administered by intravenous means on days 0 and 30 and then every 3 months at least until remission.

##### Paraclinical examinations

These are done systematically until remission is achieved:

- Blood count, including platelet levels, measurement of blood ions (electrolytes), serum creatinine, and eGFR, blood glucose, CRP, urine dipstick, and measurement of protein and creatinine in the urine sample in cases of renal dysfunction: prior to each infusion in cases involving treatment with cyclophosphamide or rituximab, administered intravenously, or every month in cases of other types of treatment.
- Measurement of albumin in cases of renal dysfunction or malnutrition, serum calcium, liver enzyme profile (AST, ALT, γGT, ALP) every month.
- Cytobacteriological urine examination (CBEU), measurement of protein/creatinine ratio (in g/g equivalent to 24-h protein in urine), to be done monthly in cases of renal dysfunction.
- Monitoring ANCA with an antigen-specific test in cases of ANCA-associated vasculitis, at the beginning of maintenance treatment.
- Electrophoresis of serum proteins and immunoglobulin quantitation (IgG, IgA, IgM) every 6 months.
- Lymphocyte subpopulations: lymphocyte count of T CD4^+^ (in cases of treatment with cytotoxic immunosuppressant medications) and B CD19^+^ (in cases of treatment with rituximab), every 6 months.

In addition to these examinations, other studies may be indicated during laboratory testing to ensure that remission has been achieved;

- Monitoring ANCA with an antigen-specific test in cases of ANCA-associated vasculitis.
- Electrocardiogram and radiological examinations, individualized on the basis of initial locations.
- Specialized consultations (ENT, ophthalmology, etc.) by virtue of initial locations.
- Imaging studies (chest and sinus scans, in particular), according to initial complaints, to obtain imaging of reference, for purposes of remission, and to be able to diagnose a possible future relapse.
- Functional studies (PFT, EMG, etc.), based on initial locations.

Disease activity score (BVAS, version 3) may help to clarify the activity of the disease if the patient is included in a therapeutic trial.

Frequency of these examinations, as well as prescription of other supplemental examinations, should be individualized during the induction treatment period according to the:

- Clinical condition of the patient.
- Severity and progression of the disease under treatment.
- Treatments given (monitoring, tolerance, adverse effects).

#### Maintenance treatment and long-term follow-up

##### Clinical exam

The follow-up clinical examination during the maintenance treatment period is performed by a hospital physician at the center managing the patient’s care, in collaboration with the generalist physician or organ specialist physician (depending on the clinical manifestations involved).

In a general way, clinical examinations are indicated:

- For patients under treatment.
  - Every 3–6 months.
  - For each administration of treatment with rituximab or during changes in dose.
- For patients who have been weaned from treatment.
  - Every 6 months, for a minimum of 2 years.
  - Then every year, for a minimum of 5–10 years, because the occurrence of late-onset relapses urges caution, in particular in cases of initial renal involvement.

Appraisal of sequelae linked to disease and treatment may begin as of the third month of treatment pursuant to the Vasculitis Damage Index (VDI) scale for patients included in a research protocol (Additional file 4).

**Parclinical examinations**

The following examinations are recommended during the monitoring period of patients under maintenance treatment:

- Complete blood count, including platelet levels, measurement of blood ions (electrolytes), serum creatinine, and eGFR, blood glucose, CRP, urine dipstick, and measurement of protein and creatinine in the urine sample, in cases of renal dysfunction, every 3 months
- Serum albumin in cases of renal dysfunction or malnutrition, serum calcium, liver enzyme profile (AST, ALT, γGT, ALP) every 3 months
- Cytobacteriological urine test (CBEU), measurement of protein/creatinine ratio (in g/g equivalent to 24-h protein in urine) every 3 months in cases of renal dysfunction
- Monitoring ANCA with an antigen-specific test in cases of ANCA-associated vasculitis, every 3–6 months, depending on the context
- Electrophoresis of serum proteins and immunoglobulin quantitation (IgG, IgA, IgM) every 6 months
- Lymphocyte subpopulations: lymphocyte count of T CD4^+^ (in cases of treatment with cytotoxic immunosuppressant medications) and B CD19^+^ (in cases of treatment with rituximab), every 6 months
- Radiological studies (chest and sinus scans, in particular), physiological studies (pulmonary function testing, electromyograms, electrocardiograms, etc.), and specialized consultations (ENT, ophthalmology, etc.) based on initial locations and future complications linked to the disease and/or treatments: every 6 months, then every year

Paraclinical examinations are indicated:

- For patients under treatment.
  - Every 3–6 months
- Among patients who have been weaned from treatment.
  - Every 6 months, for a minimum of 2 years.
  - Then every year, for a minimum of 5–10 years, because the occurrence of late-onset relapses urges caution, in particular in cases of initial renal involvement.

In addition, the frequency of these examinations, as well as the prescription of future supplemental examinations, should be individualized during maintenance treatment and toward the end of treatment, as a function of:

- Clinical condition of the patient.
- Progression of the disease under treatment, then after treatment has ended (risk of relapse).
- Future sequelae linked to the disease and/or treatments, according to a frequency dependent on the type of sequelae observed (pulmonary fibrosis, cardiac insufficiency, renal insufficiency, etc.).
- Treatments given (monitoring, tolerance, adverse effects).

#### Screening for late-onset adverse effects of treatment

The occurrence, sometimes late, of certain adverse effects of treatment administered (up to 20 years after diagnosis and treatment of the disease) requires a need for regular and extended clinical monitoring, in other words, lifelong, along with regular paraclinical examinations and/or focused tests based on the appearance of clinical signs.

This involves, in particular, the risk of bladder cancer associated with the use of cyclophosphamide, myelodysplasia, and malignant blood diseases associated with prolonged use of immunosuppressants, but also, for example, cardiovascular risk, partially linked to prolonged used of corticosteroid therapy.

##### Bladder cancer screening

Patients in question are those receiving cyclophosphamide, in particular by mouth. Prevention of this risk is of paramount importance (good hydration, complete emptying of the bladder, prescription of mesna, etc.) (Box 3). These patients should undergo regular simple monitoring for life and should have laboratory tests in cases of macroscopic hematuria, to test for urothelial cancer and bladder cancer in particular. With the use of rituximab and a reduction in the cumulative doses of cyclophosphamide, this complication should only rarely be seen in the coming decades.

##### Screening for cervical cancer in women

Patients having received immunosuppressant treatment, in particularly cyclophosphamide, should have an annual smear test. The human papillomavirus (HPV) vaccine could be proposed to patients (upon advice from a referral center and/or center for specialized care).

##### Screening for skin cancers

Annual skin checkup is also necessary to test for possible immunosuppressant-induced skin cancers, upon recommendation based on a dermatological examination with a skin and mucosal test once a year. Reminders about photoprotection measures during medical visits are equally important.

#### Socio-professional and school aspects and renewal of disabled status with long-term conditions

Socio-professional repercussions of the disease may be significant (only 40% of patients suffering from granulomatosis with polyangiitis (GPA) have continued or resumed their professional activities 3 years after diagnosis). Professional reclassification or qualification for disabled status may therefore be necessary. Stopping work is frequently indispensable for the first 6 months of treatment and may, if necessary, be followed by part-time work while undergoing therapy.

For children in elementary school or preschool, it is strongly recommended that an IEP (Individualized Educational Program) be drawn up in collaboration with the director of the educational establishment.

By virtue of the length of the initial conventional treatment (24 months at a minimum on average) and the prolonged risk of relapse which requires long-term monitoring (for 10 years at a minimum), qualification of disabled status with a long-term condition may be sought for renewable periods of 5 years.

Biology

**Table:** 

Tests	Specific situations
Proof of ANCA (IF and ELISA, see immunocapture)	Contribute to the diagnosis and determine the type of systemic necrotizing vasculitis (SNV) during the initial evaluation. Used in follow-up
Complete blood count (CBC), including platelets	Initial evaluation, management of therapy, and follow-up, and, as needed, or in cases of intercurrent events
PTT, PT, fibrinogen	Initial evaluation, management of therapy, and follow-up, and as needed, or in cases of intercurrent events
C-reactive protein (CRP)	Initial evaluation, management of therapy, and follow-up, and as needed, or in cases of intercurrent events
Total proteins Electrophoresis of serum proteins	Initial evaluation, management of therapy, and follow-up, and as needed, or in cases of intercurrent events
Immunofixation of serum proteins	In cases of hyper- or hypogammaglobulinemia detected by electrophoresis of serum proteins
Liver enzyme profile (AST, ALT, γGT, alkaline phosphatase, total bilirubin)	Initial evaluation, management of therapy, and follow-up, and as needed, or in cases of intercurrent events
Measurement of blood ions (electrolytes), serum creatinine, estimate of glomerular filtration rate by MDRD or CKD-EPI, blood glucose, serum calcium, serum phosphorus	Initial evaluation, management of therapy, and follow-up, and as needed, or in cases of intercurrent events
Determination of CPK, LDH	Pre-therapy laboratory testing
Urinary tests: protein in urine, hematuria, leukocytes in urine, nitrites	For all patients: initial evaluation and follow-up using urine dipsticks (services for which reimbursement is not provided by law)
Cytobacteriological urine test (CBEU), measurement of protein/creatinine ratio or protein in 24-h urine)	For all patients: initial evaluation and follow-up using urine dipsticks
Determination of folates, ferritin, and vitamin B_12_	In cases of abnormalities triggered by the CBC during the initial evaluation, management of therapy, and follow-up, and as needed, or in cases of intercurrent events
Antinuclear antibodies, antibodies directed against soluble nuclear antigen, anti-DNA antibodies, rheumatoid factors, anti-CCP antibodies, anti-glomerular basement membrane antibodies, cryoglobulinemia, CH50, C3, C4	Confirmation of diagnosis, according to clinical warning signs, differential diagnosis
Troponin I	Pre-therapy laboratory testing and as needed for patients with cardiac signs
B-type natriuretic peptide (BNP)	Pre-therapy laboratory testing and as needed for patients with cardiac signs
HIV serology (PCR if positive), HBV (HBs antigens; anti-HBe antibodies and viral DNA if positive or suspected serology), HCV (viral RNA if positive or suspected serology)	Diagnosis and pre-therapy laboratory testing Depending on the context, other viral serology testing may be requested, as well as other bacteriological or fungal studies
Biological monitoring of vasculitis treatment, with respect to authorizations for sale and marketing	Corticosteroids: serum potassium, serum calcium, serum phosphorus, fasting blood glucose, etc. (refer to Long-term Conditions 8 diabetes, if necessary), testing for dyslipidemia Immunosuppressants: CBC, including platelets, anticoagulants
Biological laboratory testing individualized in cases of cardiovascular risk factors	Refer to Long-term Conditions 3: obliterating arteriopathy of the lower extremities, Chapter, “Control of Cardiovascular Risk Factors”
Analysis of cerebrospinal fluid	In cases of neuromeningeal manifestations
Testing for *S. aureus* by nasal swab	Pre-therapy laboratory testing in cases of granulomatosis with polyangiitis (GPA) and follow-up care

## Supplementary Information

**Additional file 1.** Appendix 1—List of referral centers and centers for specialized care of the organization FAI^2^R for systemic autoimmune and autoinflammatory diseases.

**Additional file 2.** Appendix 2—Medications which may be associated with the occurrence of vasculitis.

**Additional file 3.** Vasculitis activity score—Birmingham vasculitis activity score version 2003.

**Additional file 4.** Appendix 4—Sequelae score—Vasculitis damage index.

**Additional file 5.** Appendix 5—List of procedures and services needed for follow-up care and treatment of long-term conditions 21—Systemic necrotizing vasculitis.

## Data Availability

Not applicable.

## Abbreviations

ACE: Angiotensin-converting enzyme
AFSSAPS: French Agency for the Heath Safety of Health Products
ALT: Alanine aminotransferase (aka SGPT)
ARA2: Angiotensin receptor II antagonists
ALP: Alkaline phosphatase
ANCA: Anti-neutrophil cytoplasmic antibodies
AOMI: Obliterative arterial disease of the lower limbs
APTT: Activated partial thromboplastin time
ARBs: Angiotensin II receptor blockers
AST: Aspartate aminotransferase (aka SGOT)
AV: ANCA vasculitis
BAL: Bronchoalveolar lavage
BVAS: Birmingham Vasculitis Activity Score
BVAS-WG: Birmingham Vasculitis Activity Score—Wegener’s granulomatosis
CBUE: Urine cytobacteriological examination
CEDIT: Technological Innovations Evaluation and Dissemination Committee
CKD-EPI: Chronic Kidney Disease Epidemiology Collaboration
CNAMTS: National Health Insurance Fund for Employed Workers
CPAMTS: Primary Health Insurance Fund for Salaried Workers
CPK: Creatine phosphokinase
CRP: C-reactive protein
CT: Computer tomography (scanner)
CYC: Cyclophosphamide
DGOS: General Management of the Care Service
DLCO: Diffusing capacity of the lungs for carbon monoxide
ECG: Electrocardiogram
EEG: Electroencephalogram
EFR: Respiratory functional explorations
EGPA: Eosinophilic granulomatosis with polyangiitis
ELISA: Enzyme-linked immunosorbent assay
EMG: Electromyogram
EUVAS: European Vasculitis Study Group
FDA: US Food and Drug Administration
FFS: Five-Factor Score
GFEV: French Vasculitis Study Group
GFR: Glomerular filtration rate
GPA: Granulomatosis with polyangiitis (previously known as Wegener’s)
HAS: French National Health Authority
HBP: High blood pressure
HBV: Hepatitis B virus
HIV: Human immunodeficiency virus
IF: Immunofluorescence
ILs: Interleukins
IV: Intravenous
IVIG: Intravenous immunoglobulin
LAS: List of acts and services
LDH: Lactate dehydrogenase
LTA: Long-term affection
MA: Marketing authorization
MDRD: Modification of Diet in Renal Disease
MPA: Microscopic polyangiitis
MPO: Myeloperoxidase
MRI: Magnetic resonance imaging
MSR: Medical Service Rendered
NAAHE: National Agency for Accreditation and Health Evaluation
ORL: Otolaryngologist
PAN: Polyarteritis nodosa
PET: Positron emission tomography
PFT: Pulmonary function test
PNDS: National Diagnostic and Care Protocol
PRTN3: Proteinase 3
PT: Prothrombin time
PTT: Temporary therapeutic protocols
RBCs: Red blood cells
RTX: Rituximab
SNV: Systemic necrotizing vasculitis
SPC: Summary of Product Characteristics
SPEP: Serum protein electrophoresis
TNF: Tumor necrosis factor
VCRC: Vasculitis Clinical Research Consortium
VDI: Vasculitis Damage Index
WBCs: White blood cells
