# Correction to: French recommendations for the management of systemic necrotizing vasculitides (polyarteritis nodosa and ANCA-associated vasculitides)

**DOI:** 10.1186/s13023-021-01787-4

**Published:** 2021-04-06

**Authors:** Benjamin Terrier, Raphaël Darbon, Cécile-Audrey Durel, Eric Hachulla, Alexandre Karras, Hélène Maillard, Thomas Papo, Xavier Puechal, Grégory Pugnet, Thomas Quemeneur, Maxime Samson, Camille Taille, Loïc Guillevin, Collaborators, Vincent Audard, Olivier Aumaitre, Karine Briot, Patrice Cacoub, Pascal Cathebras, Dominique Chauveau, Olivier Chosidow, Laurent Chouchana, Vincent Cottin, Divi Cornec, Eric Daugas, Elisabeth Diot, Nicolas Dupin, Khalil El Karoui, Olivier Fain, Pierre Gobert, Philippe Guilpain, Mohamed Hamidou, Aurélie Hummel, Marie Jachiet, Stéphane Jouneau, Noémie Jourde Chiche, Cédric Landron, Claire Le Jeunne, Jean-Christophe Lega, Xavier Mariette, Nathalie Morel, Christian Pagnoux, Philippe Remy, Frédéric Vandergheynst

**Affiliations:** 1 Internal Medicine, CHU Cochin, AP-HP, Paris, France; 2 French Vasculitis Association, Paris, France; 3 Internal Medicine, CHU Lyon, Lyon, France; 4 Internal Medicine, CHU Lille, Lille, France; 5 Nephrology, HEGP, AP-HP, Paris, France; 6 Internal Medicine, CHU Bichat, AP-HP, Paris, France; 7 Internal Medicine, CHU Toulouse, Toulouse, France; 8 Internal Medicine, CH Valenciennes, Valenciennes, France; 9 Internal Medicine, CHU Dijon, Dijon, France; 10 Pulmonology, CHU Bichat, AP-HP, Paris, France; 11 Nephrology, CHU Henri Mondor, AP-HP, Paris, France; 12 Internal Medicine, CHU Clermont-Ferrand, Paris, France; 13 Rheumatology, CHU Cochin, AP-HP, Paris, France; 14 Internal Medicine, CHU Pitié Salpêtrière, AP-HP, Paris, France; 15 Internal Medicine, CHU Saint Etienne, Paris, France; 16 Nephrology, CHU Toulouse, Paris, France; 17 Dermatology, CHU Henri Mondor, AP-HP, Paris, France; 18 Pharmacology, CHU Cochin, AP-HP, Paris, France; 19 Pulmonology, CHU Lyon, Paris, France; 20 Rheumatology, CHU Brest, Paris, France; 21 Nephrology, CHU Bichat, AP-HP, Paris, France; 22 Internal Medicine, CHU Tours, Paris, France; 23 Dermatology, CHU Cochin, AP-HP, Paris, France; 24 Internal Medicine, CHU Saint Antoine, AP-HP, Paris, France; 25 Nephrology, Rhône Durance Clinic, Avignon, Paris, France; 26 Internal Medicine, CHU Montpellier, Paris, France; 27 Internal Medicine, CHU Nantes, Paris, France; 28 Nephrology, CHU Necker, AP-HP, Paris, France; 29 Dermatology, CHU Saint Louis, AP-HP, Paris, France; 30 Pulmonology, CHU Rennes, Paris, France; 31 Nephrology, CHU Marseille, Paris, France; 32 Internal Medicine, CHU Poitiers, Paris, France; 33 Internal Medicine, CHU Lyon, Paris, France; 34 Rheumatology, CHU Kremlin-Bicêtre, AP-HP, Paris, France; 35 General Medicine, CHU Cochin, AP-HP, Paris, France; 36 Mount Sinai Hospital, Toronto, Canada; 37 Internal Medicine, CHU Brussels, Bruxelles, Belgium

## Correction to: Orphanet J Rare Dis 2020, 15(Suppl 2):351 https://doi.org/10.1186/s13023-020-01621-3

Following the publication of the original article [1], the authors requested to change the corresponding author from Eric Hachulla to Benjamin Terrier.

The new corresponding author Benjamin Terrier is shown in the authorship of this Correction as well as in that of the original article, that has now been updated.
