# Supplementary material for: French recommendations for the management of systemic necrotizing vasculitides (polyarteritis nodosa and ANCA-associated vasculitides)
Source: Orphanet J Rare Dis. 2020 Dec 29;15(Suppl 2):351. doi: 10.1186/s13023-020-01621-3 (PMC7771069; doi:10.1186/s13023-020-01621-3)
Supplement: Supplementary file 1 — Additional file 1. Appendix 1—List of referral centers and centers for specialized care of the organization FAI2R for systemic autoimmune and autoinflammatory diseases. [file 13023_2020_1621_MOESM1_ESM.pdf]

APPENDIX 1 – LIST OF REFERRAL CENTERS AND CENTERS FOR SPECIALIZED CARE OF THE ORGANIZATION FAI²R FOR SYSTEMIC AUTOIMMUNE AND AUTOINFLAMMATORY DISEASES

Referral Centers – coordinating sites and sites for ADULTS			
Referral center for rare and systemic autoimmune diseases of the North and Northwest (coordinating site)	Lille, CHU	HACHULLA	Éric
Referral center for rare and systemic autoimmune diseases of the North and Northwest (constitutive site)	Brest, CHU	DEVAUCHELLE-PENSEC	Valérie
Referral center for lupus, antiphospholipid syndrome and other rare autoimmune diseases (coordinating site)	Paris, AP-HP	AMOURA	Zahir
Referral center for lupus, antiphospholipid syndrome and other rare autoimmune diseases (constitutive site)	Martinique, CHU	DELIGNY	Christophe
Referral center for rare and systemic autoimmune diseases of Ile-de-France (coordinating site)	Paris, AP-HP	MOUTHON	Luc
Referral center for rare and systemic autoimmune diseases of Ile-de-France (constitutive site)	Le Kremlin-Bicêtre, AP-HP	MARIETTE	Xavier
Referral center for rare and systemic autoimmune diseases of Ile-de-France (constitutive site)	Paris, AP-HP	CACOUB	Patrice
Referral center for rare and systemic autoimmune diseases of Ile-de-France (constitutive site)	Paris, AP-HP	FARGE-BANCEL	Dominique
Referral center for rare and systemic autoimmune diseases of the East and Southwest (coordinating site)	Strasbourg, CHRU	MARTIN	Thierry
Referral center for rare and systemic autoimmune diseases of the East and Southwest (constitutive site)	Bordeaux, CHU	RICHEZ	Christophe
CEREMAIA referral center for autoinflammatory diseases and inflammatory amyloidosis (constitutive site)	Paris, AP-HP	GRATEAU	Gilles
CEREMAIA referral center for autoinflammatory diseases and inflammatory amyloidosis (constitutive site)	Paris, AP-HP	SAADOUN	David

Referral Centers – PEDIATRIC coordinating sites and constitutive sites			
CEREMAIA referral center for autoinflammatory diseases and inflammatory amyloidosis (coordinating site)	Le Kremlin Bicêtre	KONÉ-PAUT	Isabelle
CEREMAIA referral center for autoinflammatory diseases and inflammatory amyloidosis (constitutive site)	Montpellier, CHU	TOUITOU	Isabelle
CEREMAIA referral center for autoinflammatory diseases and inflammatory amyloidosis (constitutive site)	Versailles, CH	HENTGEN	Véronique
RAISE referral center for inflammatory rheumatism and rare and systemic autoimmune diseases of children (coordinating site)	Paris, AP-HP	QUARTIER-DIT-MAIRE	Pierre
RAISE referral center for inflammatory rheumatism and rare and systemic autoimmune diseases of children (constitutive site)	Lyon, CHU	BELOT	Alexandre
RAISE referral center for inflammatory rheumatism and rare and systemic autoimmune diseases of children (constitutive site)	Paris, AP-HP	MEINZER	Ulrich
**Centers for specialized care for ADULTS for rare and systemic autoimmune diseases**			
Center for specialized care for adults for rare and systemic autoimmune diseases	Angers, CHU	LAVIGNE	Christian
Center for specialized care for adults for rare and systemic autoimmune diseases	Amiens, CHU	DUHAUT	Pierre
Center for specialized care for adults for rare and systemic autoimmune diseases	Annecy-Genevois, CH	BEREZNE	Alice
Center for specialized care for adults for rare and systemic autoimmune diseases	Besançon, CHRU	MAGY-BERTRAND	Nadine
Center for specialized care for adults for rare and systemic autoimmune diseases	Boulogne-sur-Mer, CHU	BATAILLE	Pierre
Center for specialized care for adults for rare and systemic autoimmune diseases	Caen, CHU	AOUBA	Achille
Center for specialized care for adults for rare and systemic autoimmune diseases	Clermont-Ferrand, CHU	AUMAITRE	Olivier
Center for specialized care for adults for rare and systemic autoimmune diseases	Colmar, CH	KIEFFER	Pierre
Center for specialized care for adults for rare and systemic autoimmune diseases	Créteil, AP-HP	GODEAU	Bertrand
Center for specialized care for adults for rare and systemic autoimmune diseases	Dijon, CHU	BONNOTTE	Bernard
Center for specialized care for adults for rare and systemic autoimmune diseases	Tours, CHU	DIOT	Élisabeth

Center for specialized care for adults for rare and systemic autoimmune diseases	Grenoble, CHU	BOUILLET	Laurence
Center for specialized care for adults for rare and systemic autoimmune diseases	Guadeloupe, CHU	CORDEL	Nadège
Center for specialized care for adults for rare and systemic autoimmune diseases	Ile de la Réunion, CHU	RAFFRAY	Loïc
Center for specialized care for adults for rare and systemic autoimmune diseases	Limoges, CHU	FAUCHAIS	Anne-Laure
Center for specialized care for adults for rare and systemic autoimmune diseases	Lyon, CHU	HOT	Arnaud
Center for specialized care for adults for rare and systemic autoimmune diseases	Marseille, AP-HM	HARLE	Jean-Robert
Center for specialized care for adults for rare and systemic autoimmune diseases	Montpellier, CHU	MOREL	Jacques
Center for specialized care for adults for rare and systemic autoimmune diseases	Nancy, CHRU	WAHL	Denis
Center for specialized care for adults for rare and systemic autoimmune diseases	Nantes, CHU	HAMIDOU	Mohamed
Center for specialized care for adults for rare and systemic autoimmune diseases	Nice, CHU	FUZIBET	Jean-Gabriel
Center for specialized care for adults for rare and systemic autoimmune diseases	Paris, AP-HP	UZUNHAN	Yurdagul
Center for specialized care for adults for rare and systemic autoimmune diseases	Paris, AP-HP	PAPO	Thomas
Center for specialized care for adults for rare and systemic autoimmune diseases	Paris, GHDCSS	ZIZA	Jean-Marc
Center for specialized care for adults for rare and systemic autoimmune diseases	Paris, AP-HP	KARRAS	Georges-Alexandre
Center for specialized care for adults for rare and systemic autoimmune diseases	Paris, AP-HP	FAIN	Olivier
Center for specialized care for adults for rare and systemic autoimmune diseases	Saint-Denis, CH	LHOTE	François
Center for specialized care for adults for rare and systemic autoimmune diseases	Poitiers, CHU	ROBLOT	Pascal
Center for specialized care for adults for rare and systemic autoimmune diseases	Reims, CHU	PENNAFORTE	Jean-Loup
Center for specialized care for adults for rare and systemic autoimmune diseases	Rennes, CHU	JEGO	Patrick
Center for specialized care for adults for rare and systemic autoimmune diseases	Rouen, CHU	LEVESQUE	Herve
Center for specialized care for adults for rare and systemic autoimmune diseases	Saint-Étienne, CHU	CATHEBRAS	Pascal
Center for specialized care for adults for rare and systemic autoimmune diseases	Toulouse, CHU	CHAUVEAU	Dominique
Center for specialized care for adults for rare and systemic autoimmune diseases	Valenciennes, CH	QUEMENEUR	Thomas

Centers of specialized PEDIATRIC care			
RAISE center for specialized care of the referral center for inflammatory rheumatism and rare and systemic autoimmune diseases in children	Amiens	DJEDDI	Djamal
RAISE center for specialized care of the referral center for inflammatory rheumatism and rare and systemic autoimmune diseases in children	Angers	PELLIER	Isabelle
RAISE center for specialized care of the referral center for inflammatory rheumatism and rare and systemic autoimmune diseases in children	Belfort	LOHSE	Anne
RAISE center for specialized care of the referral center for inflammatory rheumatism and rare and systemic autoimmune diseases in children	Besançon	BALLOT-SCHMIT	Claire
RAISE center for specialized care of the referral center for inflammatory rheumatism and rare and systemic autoimmune diseases in children	Brest	DEVAUCHELLE	Valérie
RAISE center for specialized care of the referral center for inflammatory rheumatism and rare and systemic autoimmune diseases in children	Grenoble	BARBIER	Catherine
RAISE center for specialized care of the referral center for inflammatory rheumatism and rare and systemic autoimmune diseases in children	Marne-la-Vallée	AGBO-KPATI	Placide
RAISE center for specialized care of the referral center for inflammatory rheumatism and rare and systemic autoimmune diseases in children	Marseille	JURQUET	Anne-Laure
RAISE center for specialized care of the referral center for inflammatory rheumatism and rare and systemic autoimmune diseases in children	Montpellier	JEZIORSKI	Éric
RAISE center for specialized care of the referral center for inflammatory rheumatism and rare and systemic autoimmune diseases in children	Nantes	LACROIX	Sylvie
RAISE center for specialized care of the referral center for inflammatory rheumatism and rare and systemic autoimmune diseases in children	Orléans	TALMUD	Déborah
RAISE center for specialized care of the referral center for inflammatory rheumatism and rare and systemic autoimmune diseases in children	Poitiers	SOLAU-GERVAIS	Élisabeth

RAISE center for specialized care of the referral center for inflammatory rheumatism and rare and systemic autoimmune diseases in children	Rouen	GRALL-LEROSEY	Martine
RAISE center for specialized care of the referral center for inflammatory rheumatism and rare and systemic autoimmune diseases in children	Toulouse	PAJOT	Christine
RAISE center for specialized care of the referral center for inflammatory rheumatism and rare and systemic autoimmune diseases in children	Villefranche-sur-Saône	REMY-PICCOLO	Vanessa
RAISE center for specialized care of the referral center for inflammatory rheumatism and rare and systemic autoimmune diseases in children	Martinique	HATCHUEL	Yves
RAISE center of specialized case of the referral center for inflammatory rheumatism and rare and systemic autoimmune diseases in children	Paris, AP-HP	WIPFF	Julien
Center of pediatric specialized care of CEREMAIA referral center for autoinflammatory diseases and inflammatory amyloidosis	Grenoble	PAGNIER	Anne
Center of pediatric specialized care of CEREMAIA referral center for autoinflammatory diseases and inflammatory amyloidosis	Lyon, CHU	BELOT	Alexandre
Center of pediatric specialized care of CEREMAIA referral center for autoinflammatory diseases and inflammatory amyloidosis	Marseille	RETORNAZ	Karine
Center of pediatric specialized care of CEREMAIA referral center for autoinflammatory diseases and inflammatory amyloidosis	Toulouse	DECRAMER	Stéphane
Center of pediatric specialized care of CEREMAIA and RAISE referral centers for autoinflammatory disease, inflammatory amyloidosis, inflammatory rheumatism, and rare and systemic autoimmune diseases in children	Bordeaux	PILLET	Pascal
Center of pediatric specialized care of CEREMAIA and RAISE referral centers for autoinflammatory disease, inflammatory amyloidosis, inflammatory rheumatism, and rare and systemic autoimmune diseases in children	Caen	DESDOITS	Alexandra

Center of pediatric specialized care of CEREMAIA and RAISE referral centers for autoinflammatory disease, inflammatory amyloidosis, inflammatory rheumatism, and rare and systemic autoimmune diseases in children	Clermont-Ferrand	MERLIN	Étienne
Center of pediatric specialized care of CEREMAIA and RAISE referral centers for autoinflammatory disease, inflammatory amyloidosis, inflammatory rheumatism, and rare and systemic autoimmune diseases in children	Dijon	BOTTOLLIER	Élodie
Center of pediatric specialized care of CEREMAIA and RAISE referral centers for autoinflammatory disease, inflammatory amyloidosis, inflammatory rheumatism, and rare and systemic autoimmune diseases in children	Lille	REUMAUX	Héloïse
Center of pediatric specialized care of CEREMAIA and RAISE referral centers for autoinflammatory disease, inflammatory amyloidosis, inflammatory rheumatism, and rare and systemic autoimmune diseases in children	Nancy	LEMELLE	Irène
Center of pediatric specialized care of CEREMAIA and RAISE referral centers for autoinflammatory disease, inflammatory amyloidosis, inflammatory rheumatism, and rare and systemic autoimmune diseases in children	Nîmes	TRAN	Tu-Anh
Center of pediatric specialized care of CEREMAIA and RAISE referral centers for autoinflammatory disease, inflammatory amyloidosis, inflammatory rheumatism, and rare and systemic autoimmune diseases in children	Reims	PIETREMENT	Christine
Center of pediatric specialized care of CEREMAIA and RAISE referral centers for autoinflammatory disease, inflammatory amyloidosis, inflammatory rheumatism, and rare and systemic autoimmune diseases in children	Rennes	DESPERT	Véronique
Center of pediatric specialized care of CEREMAIA and RAISE referral centers for autoinflammatory disease, inflammatory amyloidosis, inflammatory rheumatism, and rare and systemic autoimmune diseases in children	Saint-Étienne	STEPHAN	Jean-Louis

Center of pediatric specialized care of CEREMAIA and RAISE referral centers for autoinflammatory disease, inflammatory amyloidosis, inflammatory rheumatism, and rare and systemic autoimmune diseases in children	Strasbourg	TERZIC	Joëlle
Center of pediatric specialized care of CEREMAIA and RAISE referral centers for autoinflammatory disease, inflammatory amyloidosis, inflammatory rheumatism, and rare and systemic autoimmune diseases in children	Tours	HOARAU	Cyrille
