# Supplementary material for: French recommendations for the management of systemic necrotizing vasculitides (polyarteritis nodosa and ANCA-associated vasculitides)
Source: Orphanet J Rare Dis. 2020 Dec 29;15(Suppl 2):351. doi: 10.1186/s13023-020-01621-3 (PMC7771069; doi:10.1186/s13023-020-01621-3)
Supplement: Supplementary file 2 — Additional file 2. Appendix 2—Medications which may be associated with the occurrence of vasculitis. [file 13023_2020_1621_MOESM2_ESM.pdf]

APPENDIX 2 – MEDICATIONS WHICH MAY BE ASSOCIATED WITH THE OCCURRENCE OF VASCULITIS

Medications often acting as the source of vasculitis
Allopurinol
Alpha-methyldopa
NSAIDS
Anti-TNF-alpha
Azathioprine
Beta-Lactam antibiotics
Cimetidine
Clozapine
D-Penicillamine
Hydantoin
Hydralazine
Ketoconazole
Levamisole
Minocycline
Phenothiazine
Propylthiouracil
Quinidine
Quinolones
Gold salts
Sulfonamides
Thiazides

Medications often implicated as the source of ANCA-associated vasculitis
**Propylthiouracil +++**
**Hydralazine +++**
Allopurinol
Anti-TNF-alpha
Ciprofloxacin
Clozapine
D-Penicillamine
Leukotriene inhibitors
Levamisole
Minocycline
Phenytoin
Sulfasalazine
Thioridazine
