# Supplementary material for: French recommendations for the management of systemic necrotizing vasculitides (polyarteritis nodosa and ANCA-associated vasculitides)
Source: Orphanet J Rare Dis. 2020 Dec 29;15(Suppl 2):351. doi: 10.1186/s13023-020-01621-3 (PMC7771069; doi:10.1186/s13023-020-01621-3)
Supplement: Supplementary file 3 — Additional file 3. Vasculitis activity score—Birmingham vasculitis activity score version 2003. [file 13023_2020_1621_MOESM3_ESM.pdf]

APPENDIX 3 – VASCULITIS ACTIVITY SCORE – BIRMINGHAM VASCULITIS ACTIVITY SCORE VERSION 2003

Appendix 1 – BVAS 2003 VASCULITIS ACTIVITY SCORE

TOTAL [ ]

Check **only** the manifestations indicating an active disease (sequelae lasting for more than 3 months are assessed by the VDI). ***If all the manifestations indicate a chronic active activity, although weakly (smoldering / grumbling disease) and there is no recent new manifestation or clear aggravation, check the box in the bottom right corner. The scores indicated are those for a recently active disease / weakly active disease, "grumbling" (bottom box checked). Add up only one of the columns..***

	**Yes**		**Yes**
**1. General Signs**	**(maximum 3 / 2)**	**6. Cardiac Signs**	**(maximum 6 / 3)**
Myalgia	1 / 1	Disappearance of a pulse	4 / 1
Arthralgias or arthritis	1 / 1	Valvular impairment	4 / 2
Fever 38°C	2 / 2	Pericarditis	3 / 1
Weight loss of 2 kg	2 / 2	Angina	4 / 2
		Cardiomyopathy	6 / 3
**2. Skin signs**	**(maximum 6 / 3)**	Congestive heart failure	6 / 3

Necrosis	2 / 1
Purpura	2 / 1
Ulceration	4 / 1
Gangrene	6 / 2
Other vasculitis related lesions	2 / 1
**3. Mucosal and ocular impairment |**	**(maximum 6 / 3)**
Mouth ulceration / granuloma	2 / 1
Genital ulceration	1 / 1
Lacrimal or salivary inflammation	4 / 2
Exophthalmos	4 / 2
Episcleritis	2 / 1
Conjunctivitis / blepharitis / keratitis	1 / 1
Gradual decrease in visual acuity/blurred vision	3 / 2
Sudden decrease in visual acuity / blindness	6 / -
Uveitis	6 / 2
Retinal vasculitis	6 / 2
Thrombosis / hemorrhage / retinal exudates	
**4. ENT Signs**	**(maximum 6 / 3)**
Epistaxis / nasal crusting	
Nasal ulceration or granuloma	6 / 3
Sinusitis	2 / 1
Subglottic stenosis	6 / 3
Reduced transmission hearing (conduction)	3 / 1
Decreased perception hearing (sensory)	6 / 2
**5. Lung Signs**	**(maximum 6 / 3)**
Wheezing / sibilants	2 / 1
Nodule(s) / Excavated Nodule(s)	3 / -
Pleural effusion	4 / 2
Radiological pulmonary infiltrate	4 / 2
Endobronchial Stenosis	4 / 2
Intra-alveolar hemorrhage	6 / 4
Respiratory distress	6 / 4

**Digestive manifestations**	**(maximum 12 / 6)**
Peritonitis	9 / 3
Bloody diarrhea	9 / 3
Abdominal pain (intestinal angina)	2 / 6
**Kidney signs**	**(maximum 12 / 6)**
HTA	4 / 1
Proteinuria > 1 +	4 / 2
Hematuria > 10 GR / field	6 / 3
Creatinine 125-249 pmol/l	4 / 2
Creatinine 250-499 pmol/l	6 / 3
Creatinine > 500 pmol/l	8 / 4
Increase in creatinine> 30% or decrease in creatinine clearance > 25%	6 / -
**Neurological impairment**	**(maximum 9 / 6)**
Headaches	1 / 1
Meningitis	3 / 1
Confusion, disorder of consciousness	3 / 1
Convulsions (not related to high blood pressure)	9 / 3
Spinal cord impairment (myelitis)	3 / 1
Stroke	9 / 3
Cranial pair impairment	9 / 3
Peripheral sensory neuropathy	6 / 3
Peripheral motor neuropathy	9 / 3

10. Other specific impairment

Specify: ………………………………………………………………………………
………………………………………………………………………………….
………………………………………………………………………………….
………………………………………………………………………………….

CHECK THIS BOX IF **ALL** OF THE REPORTED IMPAIRMENTS ARE OLD AND PERSISTENT, and not recent or worsened [ ]
