# Supplementary material for: French recommendations for the management of systemic necrotizing vasculitides (polyarteritis nodosa and ANCA-associated vasculitides)
Source: Orphanet J Rare Dis. 2020 Dec 29;15(Suppl 2):351. doi: 10.1186/s13023-020-01621-3 (PMC7771069; doi:10.1186/s13023-020-01621-3)
Supplement: Supplementary file 4 — Additional file 4. Appendix 4—Sequelae score—Vasculitis damage index. [file 13023_2020_1621_MOESM4_ESM.pdf]

APPENDIX 4 – SEQUELAE SCORE – VASCULITIS DAMAGE INDEX

MUSCULO-ARTICULAR SIGNS
- Atrophy or weakness ☐
- Erosive arthritis ☐
- Osteoporotic fracture ☐
- Aseptic osteonecrosis ☐
- Osteomyelitis ☐

CUTANEOUS-MUCOUS SIGNS
- Alopecia ☐
- Skin ulcer(s) ☐
- Oral ulceration(s) ☐

OPHTHALMOLOGICAL SIGNS
- Cataract ☐
- Retinal disease or atrophy ☐
- Decreased visual acuity / diplopia ☐
- Monocular blindness ☐
- Binocular blindness ☐
- Orbital destruction ☐

ORL SIGNS
- Loss of hearing ☐
- Obstruction, crusts, runny nose ☐
- Collapse / perforation of the nasal septum ☐
- Chronic sinusitis ☐
- Osseous destruction ☐
- Subglottic stenosis, not operated upon ☐

PULMONARY SIGNS
- PAH [Pulmonary-Arterial Hypertension] ☐
- Pulmonary fibrosis/excavations ☐
- Pulmonary infarct ☐
- Pleural fibrosis ☐
- Chronic asthma ☐
- Chronic respiratory failure ☐
- RFT [Respiratory Function Test] anomalies ☐

CARDIOVASCULAR SIGNS
- Angina or bypass ☐
- Myocardial infarct ☐
- Cardiomyopathy ☐
- Cardiac insufficiency ☐
- Valvular involvement ☐
- Pericarditis-pericardiectomy ☐
- UTA -- Diastolic BP > 95 mmHg and/or treated ☐

PERIPHERAL VASCULAR SIGNS
- Pulse abolition ☐
- Stenosis of a large vessel ☐
- Arterial claudication ☐
- Complicated phlebitis ☐

DIGESTIVE SIGNS
- Infarct / intestinal resection ☐
- Digestive-mesenteric claudication ☐
- Pancreatitis > 3 months ☐
- Chronic peritonitis ☐
- Esophageal stenosis ☐

KIDNEYS
- Decrease in clearance > 50% ☐
- Proteinuria > 0.5 g / day ☐
- Chronic renal failure ☐
- Dialysis ☐

NERVOUS SYSTEM
- Major cognitive impairment or psychosis ☐
- Epilepsy ☐
- Stroke ☐
- Cranial nerve damage ☐
- Peripheral neuropathy ☐
- Transverse myelitis ☐

OTHER SEQUALAE
- Menopause ☐
- Cancer ☐
- Bladder cystitis / neoplasia linked with cyclophosph. ☐
- Describe ☐

TOTAL = ☐ (= numbers of boxes checked)
