# Supplementary material for: French recommendations for the management of systemic necrotizing vasculitides (polyarteritis nodosa and ANCA-associated vasculitides)
Source: Orphanet J Rare Dis. 2020 Dec 29;15(Suppl 2):351. doi: 10.1186/s13023-020-01621-3 (PMC7771069; doi:10.1186/s13023-020-01621-3)
Supplement: Supplementary file 5 — Additional file 5. Appendix 5—List of procedures and services needed for follow-up care and treatment of long-term conditions 21—Systemic necrotizing vasculitis. [file 13023_2020_1621_MOESM5_ESM.pdf]

APPENDIX 5 – LIST OF PROCEDURES AND SERVICES NEEDED FOR FOLLOW-UP CARE AND TREATMENT OF LONG-TERM CONDITIONS 21 – SYSTEMIC NECROTIZING VASCULITIS

Regulatory Basis

Law No. 2004-810 of August 13, 2004 pertaining to health insurance, has created the High Public Health Authority and has established its mission, specifically within the area of long-term disorders (as per Article R. 161-71 of the Social Security Code).

Article 6 amends Article L.322-3 of the Social Security Code, which defines the circumstances whereby the insured is released from the payment of the patient's contribution, and Article L.324-1 of that same code, which establishes obligations arising in cases of long-term conditions, specifically those specifying a protocol for care provided in collaboration between the treating physician and the Social Security consulting physician. This protocol is signed by the patient or his/her legal representative.

Pursuant to its mission, established in Decree No. 2004-1139 of October 26, 2004, the High Public Health Authority has formulated recommendations for procedures and services needed in the treatment of conditions listed in Article L.324-1 for which contribution by the insured party may be limited or eliminated, in application of No. 3 in Article L.322-3.

These recommendations pertain, if applicable, to the conditions for which procedures and services must be performed. The list of procedures and services which are provided to target systemic necrotizing vasculitis (SNV), as well as all services which appear to be justified for proper management of a disease considered as a long-term condition during outpatient follow-up care. This should be based on care protocols for patients with long-term conditions, with the knowledge that certain specific situations involving complications leading to hospitalization may be the reason for procedures and care not listed here.

In cases when the disease worsens, or if there are complications or adverse effects linked to treatment, consultations with hospital physicians, private doctors, and/or treating physicians, and/or supplemental exams, may, in fact, be necessary.

Medical and paramedical procedures

Healthcare Professionals	Specific situations
**System Resources**	
**Physicians at the referral center and/or center for specialized care, Group I – rare and systemic and autoimmune diseases – necrotizing vasculitis: internists, rheumatologists, pulmonologists, pediatricians, nephrologists**	Initial evaluation, management of therapy, and follow-up
**Hospital physicians at an unclassified center, a collaborating institution with the referral center, and/or a center for specialized care, Group I – rare and systemic and autoimmune diseases – necrotizing vasculitis: internists, rheumatologists, pulmonologists, pediatricians, nephrologists.**	Initial evaluation, management of therapy, and follow-up
**Treating Physician**	Initial evaluation, management of therapy, and follow-up
**Radiologist**	Initial evaluation, management of therapy, and follow-up
**Biologist**	Initial evaluation, management of therapy, and follow-up
**Nurses**	Initial evaluation, management of therapy, and follow-up
**Nursing Assistants**	Management of therapy, and follow-up (services for which reimbursement is not provided by law except within the framework of lump-sum payments for home nursing care services)
**Resources in the event of need**	
**All other specialists for disease- and/or treatment related complications**	In cases of a specific complaint (for example, ENT, ophthalmology, cardiology, neurology, geriatrics, dermatology…) – Initial evaluation, management of therapy, and follow-up
**Anatomical Pathologist**	In cases of organ or tissue biopsy
**Dentist, oral surgeon**	Specific complaints (gingivitis) and complications from treatments
**Gynecologist**	Prevention of risks linked to treatment and follow-up for high-risk pregnancies
**Physician specializing in physical medicine and rehabilitation**	Rehabilitation if necessary
**Dietitian**	Management of therapy, and follow-up (services for which reimbursement is not provided by law)
**Physical therapist**	Initial evaluation (testing), management of therapy, and follow-up
**Orthoptist, Speech therapist**	Rehabilitation if necessary
**Occupational therapist**	Management of therapy, and follow-up (services for which reimbursement is not provided by law)
**Psychologist**	Initial evaluation, management of therapy, and follow-up (services for which reimbursement is not provided by law)
**Healthcare professionals involved in individualized lab testing in cases of cardiovascular risk factors**	Refer to Long-term Conditions 3, obliterating arteriopathy of the lower extremities, Chapter on "Control of Cardiovascular Risk Factors".

Patient information and therapeutic education

Information and education regarding therapy represent an important dimension in the activities of various healthcare professionals. They must monitor the involvement of patients (and his or her loved ones): information about the disease and its treatments, adjustment in lifestyle…

This information should entail:

- Vasculitis, its symptoms, specifying the warning signs of a possible flare-up, which could lead to a medical consultation.
- Prescribed treatments, possible adverse effects of treatment.
- Scheduling of routine exams or screening tests for future complications of the disease and/or treatments and the results of such exams.

These actions aimed at education require the cooperation of various healthcare professionals, who may intervene with individual procedures with patients or in an educational group. These actions can exist today, but such coordinated management is not always available. An integrated contractual understanding about the intervention of various healthcare professionals is preferable to the juxtaposition of uncoordinated procedures.

Healthcare professionals and their patients must be informed about the existence of patient associations. These associations contribute to overall improved management of the disease, by promoting cooperation and the diffusion of information among patients, as well as medical-social administration, health insurance funds, and healthcare providers.

Biology

Tests	Specific Situations
**Proof of ANCA (IF and ELISA, see immunocapture)**	Contribute to the diagnosis and determine the type of systemic necrotizing vasculitis (SNV) during the initial evaluation. Used in follow-up.
**Complete blood count (CBC), including platelets**	Initial evaluation, management of therapy, and follow-up, and, as needed, or in cases of intercurrent events.
**PTT, PT, fibrinogen**	Initial evaluation, management of therapy, and follow-up, and as needed, or in cases of intercurrent events.
**C reactive protein (CRP)**	Initial evaluation, management of therapy, and follow-up, and as needed, or in cases of intercurrent events.
**Total proteins**<br>**Electrophoresis of serum proteins**	Initial evaluation, management of therapy, and follow-up, and as needed, or in cases of intercurrent events.
**Immunofixation of serum proteins**	In cases of hyper- or hypogammaglobulinemia detected by electrophoresis of serum proteins.
**Liver enzyme profile (SGOT, SGPT, γGT, alkaline phosphatase, total bilirubin)**	Initial evaluation, management of therapy, and follow-up, and as needed, or in cases of intercurrent events.
**Measurement of blood ions (electrolytes), serum creatinine, estimate of glomerular filtration rate by MDRD or CKD-Epi, blood glucose, serum calcium, serum phosphorus**	Initial evaluation, management of therapy, and follow-up, and as needed, or in cases of intercurrent events.

**Determination of CPK, LDH**	Pre-therapy lab testing.
**Urinary tests: protein in urine, hematuria, leukocytes in urine, nitrites**	For all patients: Initial evaluation and follow-up using urine dipsticks (services for which reimbursement is not provided by law).
**Cytobacteriological urine test (CBEU), measurement of protein/creatinine ratio or protein in 24-hour urine)**	For all patients: Initial evaluation and follow-up using urine dipsticks.
**Determination of folates, ferritin, and vitamin B12**	In cases of abnormalities triggered by the complete blood count during the initial evaluation, management of therapy, and follow-up, and as needed, or in cases of intercurrent events.
**Antinuclear antibodies, antibodies directed against soluble nuclear antigen, anti-DNA antibodies, rheumatoid factors, anti-CCP antibodies, anti-glomerular basement membrane antibodies, cryoglobulinemia, CH50, C3, C4**	Confirmation of diagnosis, according to clinical warning signs, differential diagnosis.
**Troponin I**	Pretherapy lab testing and as needed for patients with cardiac signs.
**B-type natriuretic peptide (BNP)**	Pretherapy lab testing and as needed for patients with cardiac signs.
**HIV serology (PCR if positive), HBV (HBs antigens; anti-HBe antibodies and viral DNA if positive or suspected serology), HCV (viral RNA if positive or suspected serology)**	Diagnosis and pretherapy lab testing<br>Depending on the context, other viral serology testing may be requested, as well as other bacteriological or fungal studies.
**Biological monitoring of vasculitis treatment, with respect to authorizations for sale and marketing**	Corticosteroids: serum potassium, serum calcium, serum phosphorus, fasting blood glucose… (refer to Long-term Conditions 8 diabetes, if necessary), testing for dyslipidemia.<br>Immunosuppressants: Complete blood count (CBC), including platelets,<br>Anticoagulants
**Biological lab testing individualized in cases of cardiovascular risk factors**	Refer to Long-term Conditions 3: obliterating arteriopathy of the lower extremities, Chapter, “Control of Cardiovascular Risk Factors”.
**Analysis of cerebrospinal fluid**	In cases of neuromeningeal manifestations
**Testing for *Staphylococcus aureus* by nasal swab**	Pre-therapy lab testing in cases of granulomatosis with polyangiitis (GPA) and follow-up care.

Technical Procedures

Procedures	Specific Situations
**Biopsy of an affected organ or tissue**	Definitive diagnosis during initial evaluation, then during treatment and follow-up in cases of doubt regarding potential relapse.
**Chest x-ray**	Initial evaluation, management of therapy, and follow-up, and in cases of intercurrent events, or if there are clinical warning signs.
**CT scan of chest**	In cases of clinical signs or abnormalities in the chest x-ray, as needed, or in cases of intercurrent events.
**CT scan of ENT (sinuses)**	In cases of clinical signs or abnormalities in the x-ray or in the chest CT scan, and in cases of intercurrent events.
**CT scan of abdomen-pelvis**	In cases of clinical signs or abnormalities in the intestinal ultrasound, and in cases of intercurrent events.
**Digestive endoscopy**	In cases of clinical warning signs, intended for diagnostic purposes.
**Celiomesenteric and/or renal angiography**	In cases of digestive and/or renal clinical signs, in particular, if a polyarteritis nodosa (PAN) is suspected, and in cases of intercurrent events.

**Audiogram**	For patients showing ENT signs.
**Bone densitometry**	For patients on corticosteroids.
**CT scan and/or MRI of the brain and/or spine (with vascular study)**	For patients showing neurological signs.
**Electroencephalogram**	For patients showing neurological signs.
**Brain angiography**	For patients showing neurological signs, as a supplement to other neurological studies.
**EMG**	Initial evaluation, management of therapy, and follow-up care for patients exhibiting symptoms suggestive of peripheral neuropathy.
**Resting EKG**	Initial evaluation, management of therapy, and follow-up care, as needed, or in cases of intercurrent events.
**Echocardiography with Doppler**	Initial evaluation, management of therapy, and follow-up care, as needed, or in cases of intercurrent events, in particular for patients exhibiting cardiac or pulmonary signs.
**Vascular ultrasound with Doppler**	Initial evaluation, management of therapy, and follow-up care, and in cases of intercurrent events, or as needed for patients showing cardiac or neurovascular signs.<br>Follow-up for potential complications arising from prolonged corticosteroid therapy (early atherosclerosis).
**Cardiac MRI**	Pre-therapy lab testing and follow-up, as needed for patients showing cardiac signs.
**Thallium scintigraphy**	Pre-therapy lab testing and follow-up, as needed for patients showing cardiac signs.
**Pulmonary function tests**	Initial evaluation, management of therapy, and follow-up care, as needed, or in cases of intercurrent events, in particular for patients showing cardiac or pulmonary signs.
**Bronchial fibroscopy and bronchial-alveolar washing**	Initial evaluation and as needed.<br>Bronchial-alveolar washing to detect and measure an alveolar hemorrhage and for diagnosis of infections.
**Plasma Exchanges**	Depending on the indication in combination with corticosteroids and/or immunosuppressants.
**Capillaroscopy**	In cases of Raynaud's syndrome or vasomotor dysfunction of the extremities.

Treatments

Drug Treatments	Specific Situations
**Corticosteroid injectables (methylprednisolone for intravenous bolus), oral prednisone**	Induction and maintenance treatment.
**Immunosuppressants: cyclophosphamide, azathioprine, methotrexate, mycophenolate mofetil**	Induction and maintenance treatment.<br>Cyclophosphamide: Authorization for sale and marketing for granulomatosis with polyangiitis (GPA).<br>Azathioprine: Authorization for sale and marketing for systemic necrotizing vasculitis (SNV) (with the exception of viral diseases).<br>Methotrexate: off-label<br>Mycophenolate mofetil: off-label
**Rituximab**	Induction and maintenance treatment<br>Authorization for sale and marketing for treatment to induce remission and maintenance treatment for adult patients suffering from granulomatosis with polyangiitis (GPA) and microscopic polyangiitis (MPA)

**Other immunosuppressants or biologics: infliximab, abatacept, polyvalent immunoglobulins**	Off-label, in cases of disease refractory to conventional treatments with infliximab, abatacept and intravenous immunoglobulins
**Other specific immunosuppressant treatments (cyclosporine, leflunomide)**	Special treatment for certain forms of the disease, after urgent authorization from a referral center and/or center for specialized care and/or within the framework of a research protocol.
**Antiviral: interferon, pegylated interferon, entecavir, tenofovir, lamivudine…**	Treatment of forms of polyarteritis nodosa (PAN), linked to hepatitis B viral infection (see chronic hepatitis B on the List of Procedures and Services for Long-term Conditions 6).<br>For preemptive treatment of viral B reactivation after guidance from a liver specialist.
**Sulfamethoxazole/trimethoprim (cotrimoxazole)**	Prevention of infections due to *Pneumocystis jiroveci* for patients on cyclophosphamide and prevention from relapse in cases of granulomatosis with polyangiitis (GPA).
**Atovaquone**	For prevention of infection due to *Pneumocystis jiroveci* (in cases of allergy or intolerance to sulfamethoxazole/trimethoprim and the impossibility or contraindication for pentamidine aerosols) (off-label).
**Pentamidine Aerosols**	For prevention of infection due to *Pneumocystis jiroveci* (in cases of allergy or intolerance to sulfamethoxazole/trimethoprim).
**Tuberculin test (5 units) or QuantiFERON**	In cases of tuberculosis contagion and pre-therapy lab testing.
**Rifampicin, isoniazid**	For prevention of tuberculosis in patients with a history of tuberculosis or recent contact with someone infected with tuberculosis.
**Mupirocin ointment**	Discussed in cases of an ENT complaint during granulomatosis with polyangiitis (GPA) (as a carrier of *Staphylococcus aureus*, documented at least once).
**Physiologic solution**	For abundant washing of the nasal cavities, if necessary (service for which reimbursement is not provided by law).
**Eye drops for moistening and washing of the eye**	For application to the eye as necessary (service for which reimbursement is not provided by law)
**Analgesics, Level I, II, III**	In the event of pain
**Benzodiazepines, neuroleptic agents (chlorpromazine), antidepressants, certain antiepileptic drugs**	In cases of mood disorders linked to the disease and/or treatments.<br>In cases of neurogenic pain, cenesthesia, paresthesia, and/or dysesthesia (off-label for benzodiazepines and neuroleptic agents).
**Hypnotic drugs**	In cases of sleep disorders linked to pain or use of corticosteroids
**Antiepileptic drugs**	In cases of epilepsy.
**Anti-asthmatic drugs**	For patients who have or have had eosinophilic granulomatosis with polyangiitis (EGPA).<br>Refer to the List of Procedures and Services for Long-Term Conditions 14, severe chronic respiratory insufficiency due to asthma.
**Anticoagulants**	In cases of arterial and/or venous thrombosis (curative treatment) and preventive treatment in situations of risk, in particular, during flare-ups of granulomatosis with polyangiitis (GPA) and ANCA-associated vasculitis.
**Prevention and treatment of associated cardiovascular risk factors: treatments**<br>- **Antihypertensive drug(s)**<br>- **Antidiabetic drug(s)**<br>- **Lipid-reducing drugs(s)**<br>- **Platelet antiaggregation drug(s)**	Refer to the List of Procedures and Services, "Control of Cardiovascular Risk Factors" (on the list for Long-term Conditions 3: Obliterating arteriopathy of the lower extremities and on the List of Procedures and Services for Long-term Conditions 8: diabetes.
**Vasodilator(s)**	Distal ischemia and/or Raynaud's syndrome.<br>- Only naftidrofuryl has been evaluated as qualifying for a Medical Service Provided (SMR)[1] (qualified as weak) for obliterating arteriopathy of the lower extremities (insufficient for other

	indications).
**Platelet antiaggregation drugs**	Distal ischemia or cardiovascular disease.
**Prevention and treatment of adverse effects of drugs prescribed for vasculitis**	Corticosteroid therapy.<br>▪ Supplementation with calcium and vitamin D, bisphosphonates for purposes of prevention and treatment of osteoporosis.<br>▪ Supplementation with potassium.<br>▪ Proton-pump inhibitors, gastric protection not taken in proximity to corticosteroid therapy (off-label as primary prevention).<br>▪ Hypnotic agents/sleeping pills/antidepressants, anti-anxiety drugs.<br>▪ Treatment of glaucoma.<br>Cyclophosphamide: Mesna for prevention of bladder toxicity.<br>Immunosuppressants: prevention of sterility, contraception, prevention of teratogenic risk, induced by certain immunosuppressants (prescription of estrogen-progestin, LHRH analogues (off-label)).
**Proton-pump inhibitors, gastric protection**	Treatment of gastric ulcerations, gastritis and/or gastroesophageal reflux linked to disease; prevention of stress ulcers (off-label).
**Anti-pneumococcal and anti-influenza vaccination**	All patients.<br>Indications for vaccination remain in dispute for patients suffering from eosinophilic granulomatosis with polyangiitis (EGPA). This has been contraindicated for a long time by virtue of the flare-ups occurring after vaccination or desensitization. In any case, the risk of serious infectious diseases in immunosuppressed patients argues in favor of vaccination.
**Vitamins B1, B6, PP**	In cases of peripheral neuropathy (off-label).
**Antihistamines**	In cases of allergy and/or asthma,<br>By general and/or local means of administration (eye drops, ointment…)
**Folic or folinic acid**	In cases of low levels and/or anemia.<br>In cases of prescription of methotrexate (10 to 15 mg/week).
**Supplementation with iron, folates, and other vitamins**	In cases of low levels and/or anemia (vitamins: services for which reimbursement is not provided by law, except for vitamin B12 and folates).
**Antiemetic agents, antidiarrheal drugs, laxatives**	In cases of digestive disorders induced and/or aggravated by the disease and/or treatments.
**Topical corticosteroids, creams and hydrating preparations, and healing agents**	In cases of skin or mucosal lesions (services for which reimbursement is not provided by law).
**Red cell transfusion and/or erythropoietin**	In cases of anemia linked to the disease and/or treatments.
**IVIG at immune-substitutive doses**	In cases of secondary symptomatic immunological deficit satisfying the following criteria:<br>- Deficiency in the production of antibodies with immunoglobulin quantitation of IgG < 4 g/l;<br>- Associated with repeated infections requiring hospitalization;<br>After validation in a multidisciplinary coordination meeting.

Other treatments (non-pharmacological)

Devices and/or surgery	Specific situations
**Surgery**	If necessary by virtue of conditions linked to the disease (intestinal perforations, tracheal stenoses, valve and/or vascular surgery, kidney transplantation, nose reconstructive surgery, urological surgery,…) and/or treatments (corticosteroid-induced cataracts, osteonecrosis, fractures, …).
**Interventional vascular radiology**	In cases of stenosis or aneurysm already ruptured and/or threatening to rupture, accessible to intravascular techniques, complications from invasive techniques.
**Hearing correction (prosthesis)**	Hearing disorders in relation to the condition.
**Vision correction**	Vision disorders in relation to the condition and its treatment.
**Oxygen therapy and assisted ventilation**	Chronic respiratory insufficiency.
**Non-invasive ventilation or endotracheal dilator prosthesis**	Respiratory insufficiency (essentially acute) and/or stenosis of the upper airways in relation to the condition.
**Enteral nutrition and/or hypercaloric supplements**	In cases of severe changes in the patient's general condition or weight loss.
**Cryopreservation of eggs (ovarian strips), gametes or embryos**	In cases of prescription of immunosuppressive treatment potentially acting as a source of irreversible sterility, reduced fertility and/or teratogenicity (Cryopreservation of eggs outside the nomenclature).
**Kidney dialysis (hemodialysis and/or peritoneal dialysis)**	In cases of severe acute and/or chronic renal insufficiency.
**Pacemakers/intracavity cardiac stimulator (and/or, very rarely, implantable defibrillators)**	In cases of conduction and/or rhythm disorders.
**All other devices tailored to the handicap or disability: Crutches, body braces, day and/or night braces, orthotics, orthopedic shoes, walkers, manual or electric wheelchairs, anti-bedsore mattresses and cushions, toilet seat risers, hospital beds, nasal-gastric or gastrostomy tubes.**	If necessary, by virtue of the handicap or disability. (toilet seat risers: outside the List of Procedures and Services)

List of useful links for health professionals and patients

Information intended for health professionals

GFEV – French Vasculitis Study Group, www.vascularites.org
FAI²R – Health System for Rare Autoimmune and Auto-Inflammatory Diseases, www.fai2r.org
Orphanet, www.orpha.net
Respifil – Health Sector for Rare Respiratory Diseases, www.respifil.fr
SNFMI – French National Society for Internal Medicine, www.snfmi.org
SFR – French Rheumatology Society, sfr.larhumatologie.fr
SFD – French Society of Dermatology, www.sfdermato.org
SPLF – Society of Pneumology in the French Language, splf.fr
SFNDT – French Society of Nephrology, Dialysis and Transplantion, www.sfndt.org

Reference Center for Rare Lung Diseases, www.maladies-pulmonaires-rares.fr
SOFREMIP – Francophone Society for Rheumatology and Pediatric Inflammatory Diseases, www.sofremip.sfpediatrie.com
CRI –Rhumatisms and Inflammation Club, www.cri-net.com
EUVAS – European Vasculitis Study Group, www.vasculitis.org
VCRC – Vasculitis Clinical Research Consortium, www.rarediseasesnetwork.org/cms/vcrc
CanVasc – Website for the Canadian Research Group for Vasculitis, www.canvasc.ca
Reference Center for Teratogens (www.lecrat.fr)

Information intended for patients

GFEV – French Vasculitis Study Group, www.vascularites.org
French Association of Vasculitis, www.association-vascularites.org
Rare Diseases Alliance, www.alliance-maladies-rares.org
EURORDIS – Federation of Associations of Patients and Inviduals Active in the Field of Rare Diseases, www.eurordis.org
FAI²R – Health System for Rare Autoimmune and Auto-Inflammatory Diseases www.fai2r.org
FMO – Federation of Negleted Diseases, www.maladies-orphelines.fr
Rares Disease Info Services, www.maladiesraresinfo.org
Annual TPE (Therapeutic Patient Education) https://etpmaladiesrares.com/
Cortisone Info http://www.cortisone-info.fr/
Rare Diseases Info Service : http://www.maladiesraresinfo.org/
Health Info and Rights Hotline 01 53 62 40 30
AFVD Francophone Association for overcoming pain, www.association-afvd.com
